# Investigation of the causal association between Parkinson’s disease and autoimmune disorders: a bidirectional Mendelian randomization study

**DOI:** 10.3389/fimmu.2024.1370831

**Published:** 2024-05-07

**Authors:** Junyi Yang, Weiran Lin, Yumei Ma, Hui Song, Changqing Mu, Qian Wu, Chen Han, Jian Zhang, Xu Liu

**Affiliations:** ^1^ Department of Neurology, First Affiliated Hospital of China Medical University, Shenyang, Liaoning, China; ^2^ Department of Cell Biology, Key Laboratory of Cell Biology, National Health Commission of the People's Republic of China, China Medical University, Shenyang, Liaoning, China; ^3^ Key Laboratory of Medical Cell Biology, Ministry of Education of the People's Republic of China, China Medical University, Shenyang, Liaoning, China; ^4^ Department of Laboratory Medicine, General Hospital of Northern Theater Command, Shenyang, Liaoning, China

**Keywords:** Mendelian randomization, Parkinson’s disease, autoimmune diseases, causal effect, irritable bowel syndrome, type 1 diabetes

## Abstract

**Background:**

To date, an increasing number of epidemiological evidence has pointed to potential relationships between Parkinson’s disease (PD) and various autoimmune diseases (AIDs), however, no definitive conclusions has been drawn about whether PD is causally related to AIDs risk.

**Methods:**

By employing summary statistics from the latest and most extensive genome-wide association studies (GWAS), we performed a bidirectional two-sample Mendelian randomization (MR) analysis to investigate the causal associations between PD and a variety of 17 AIDs, encompassing multiple sclerosis, neuromyelitis optica spectrum disorder, myasthenia gravis, asthma, inflammatory bowel disease, Crohn’s disease, ulcerative colitis, irritable bowel syndrome, celiac disease, primary biliary cirrhosis, primary sclerosing cholangitis, type 1 diabetes, ankylosing spondylitis, rheumatoid arthritis, systemic lupus erythematosus, psoriasis and vitiligo. Inverse-variance weighted (IVW) was adopted as the main statistical approach to obtain the causal estimates of PD on different AIDs, supplemented by a series of complementary analyses (weighted median, MR Egger regression, and MR-PRESSO) for further strengthening the robustness of results.

**Results:**

Our MR findings suggested that genetically predicted higher liability to PD was causally associated with a decreased risk of irritable bowel syndrome (OR = 0.98; 95% CI: 0.96-0.99; *P* = 0.032). On the contrary, IVW analysis showed a potential positive correlation between genetically determined PD and the incidence of type 1 diabetes (OR = 1.10; 95%CI: 1.02-1.19; *P* = 0.010). Subsequent MR tests ended up in similar results, confirming our findings were reliable. Additionally, in the reverse MR analyses, we did not identify any evidence to support the causal relationship of genetic predisposition to AIDs with PD susceptibility.

**Conclusion:**

In general, a bifunctional role that PD exerted on the risk of developing AIDs was detected in our studies, both protecting against irritable bowel syndrome occurrence and raising the incidence of type 1 diabetes. Future studies, including population-based observational studies and molecular experiments *in vitro* and *in vivo*, are warranted to validate the results of our MR analyses and refine the underlying pathological mechanisms involved in PD-AIDs associations.

## 1 Introduction

Parkinson’s disease (PD) is widely perceived as the second most common neurodegenerative disease and the most prevalent movement disorder throughout the globe, characterized by a diverse array of clinical features including rigidity, bradykinesia, resting tremor, and postural instability (1). The number of patients suffering from PD worldwide has continued to rise substantially from 4.1 million in 2005 to 8.7 million by the year 2030 (2). Although the exact etiology remains elusive, a generally accepted hypothesis suggests that PD is a complicated multifactorial disease with interactions between toxic environmental factors and genetic determinants (3). Moreover, many recent studies indicated that in PD animal models as well as PD patients, a continuous inflammatory response was invariably observed at both the central nervous system (CNS) and peripheral levels (4–8). In the toxin-based PD rat models, CNS inherent immune cells, microglial cells, in the nigral tissue, could be over-activated to release substantial pro-inflammatory cytokines, such as tumor necrosis factor (TNF) and interleukin-6 (IL-6) (4). Besides, these centrally produced cytokines then flowed into the peripheral circulation through subarachnoid granules and/or lymphatic drainage, thereby regulating the inflammatory state of circulating myeloid cells (9). Reciprocally, a wide range of pro-inflammatory mediators generated by leukocytes in the peripheral blood were able to cross the blood-brain barrier (BBB) and activate microglia and astrocytes, which could alter the CNS neuroimmune environment (9). Thus, due to the central-peripheral immune crosstalk, it is particularly worth exploring whether PD is related to autoimmune diseases (AIDs) risk and vice versa.

Currently, an increasing number of observational studies reported the presence of comorbidities between PD and various autoimmune diseases. However, the epidemiological evidence on the possible relationships between PD and AIDs was seemingly inconsistent. A case-control study recruiting 3,276 participants from Mayo Clinic Biobank illustrated that individuals who self-reported a family history of Parkinson’s disease among their first-degree relatives were 30% less likely to develop rheumatoid arthritis (RA) (10). Other large-scale retrospective cohort research held a similar view that people with RA and systemic lupus erythematosus (SLE) exhibited a significantly reduced susceptibility to PD when compared to healthy individuals (11, 12). Conversely, a meta-analysis involving 833,004 patients and 10,175,890 controls demonstrated that ankylosing spondylitis (AS) and other AIDs were associated with a higher incidence of PD (13). Owing to uncontrolled confounding factors, reverse causality and limited sample size, these epidemiological investigations were prone to bias hindering the accurate inference of causal associations between PD and AIDs.

Mendelian randomization (MR) is a powerful technique that seeks to establish the causative link between exposure and outcome by utilizing genetic variations as instrumental variables (IVs) (14). In terms of overcoming the traditional bias present in observational studies, MR exhibited prominent advantages in eliminating reverse causation and avoiding the risk of confounding factors. Therefore, we conducted this bidirectional two-sample MR analysis to reveal the causal correlations between Parkinson’s disease and a broad spectrum of 17 autoimmune disorders, including multiple sclerosis (MS), neuromyelitis optica spectrum disorder (NMOSD), myasthenia gravis (MG), asthma, inflammatory bowel disease (IBD), Crohn’s disease (CD), ulcerative colitis (UC), irritable bowel syndrome (IBS), celiac disease (CeD), primary biliary cirrhosis (PBC), primary sclerosing cholangitis (PSC), type 1 diabetes (T1D), AS, RA, SLE, psoriasis and vitiligo.

## 2 Methods

### 2.1 Study design, data sources and instrument selection

According to the core principles and main assumptions of MR, single-nucleotide polymorphisms (SNPs) used as the instrumental variables (IVs) need to satisfy three criteria: (1) relevance assumption: SNPs should be associated with the exposure of interest, (2) independence assumption: the genetic variants are independent of confounders and (3) exclusion restriction assumption which proposes that SNPs should only affect outcome exclusively through path of the exposure (15). Considering that the data in our study were obtained from the publicly available genome-wide association studies (GWAS) database of PD and AIDs, no additional ethic approval and informed consent were required.

As shown in **Table 1**, we included the summary statistics for susceptibility to PD from the most comprehensive and updated genome-wide meta-analyses, which comprised 33,674 cases and 449,056 controls of European ancestry from International Parkinson’s Disease Genomics Consortium, Systems genomics of Parkinson’s disease consortium and UK Biobank (16). With regard to the IVs associated with diverse AIDs, we extracted genetic variants from a total of 17 GWASs involving MS (47,429 cases and 68,374 controls) (17), NMOSD (215 cases and 1,244 controls) (18), MG (1,873 cases and 36,370 controls) (19), asthma (56,167 cases and 352,255 controls) (20), IBD (12,882 cases and 21,770 controls) (21), CD (5,956 cases and 14,927 controls) (21), UC (6,968 cases and 20,464 controls) (21), IBS (53,400 cases and 433,201 controls) (22), CeD (12,041 cases and 12,228 controls) (23), PBC (8,021 cases and 16,489 controls) (24), PSC (2,871 cases and 12,019 controls) (25), T1D (9,266 cases and 15,574 controls) (26), AS (10,619 cases and 15,145 controls) (27), RA (14,361 cases and 43,923 controls) (28), SLE (5,201 cases and 9,066 controls) (29), psoriasis (10,588 cases and 22,806 controls) (30) and vitiligo (4,680 cases and 39,586 controls) (31). Details of recruitment procedures and diagnostic standards were shown in the original publications.

**Table 1:** Characteristics of the GWAS cohorts involving Parkinson’s disease and 17 autoimmune diseases and results of the power analysis.

Traits	Data Source/Reference	Sample size (cases/controls)	Population	Forward min OR	Reverse min OR
Parkinson’s disease (16)	IPDGC-NeuroX, UK Biobank, SGPD, IPDGC	482,730 (33,674/449,056)	European	–	–
Multiple sclerosis (17)	IMSGC	115,803 (47,429/68,374)	European	1.16	1.11
Neuromyelitis optica spectrum disorder (18)	GWAS by Estrada et al.	1,459 (215/1,244)	European	2.97	NA
Myasthenia gravis (19)	GWAS by Chia et al.	38,243 (1,873/36,370)	European	1.55	1.25
Asthma (20)	GWAS by Valette et al.	408,422 (56,167/352,255)	European	1.11	1.15
Inflammatory bowel disease (21)	GWAS by Liu et al.	34,652 (12,882/21,770)	European	1.30	1.05
Crohn’s disease (21)	GWAS by Liu et al.	20,883 (5,956/14,927)	European	1.42	1.04
Ulcerative colitis (21)	GWAS by Liu et al.	27,432 (6,968/20,464)	European	1.37	1.07
Irritable bowel syndrome (22)	GWAS by Eijsbouts et al	486,601 (53,400/433,201)	European	1.11	1.51
Celiac disease (23)	GWAS by Trynka et al.	24,269 (12,041/12,228)	European	NA	1.04
Primary biliary cirrhosis (24)	GWAS by Cordell et al.	24,510 (8,021/16,489)	European	NA	1.05
Primary sclerosing cholangitis (25)	GWAS by Ji et al.	14,890 (2,871/12,019)	European	NA	1.09
Type 1 diabetes (26)	GWAS by Forgetta et al.	24,840 (9,266/15,574)	European	1.36	1.06
Ankylosing spondylitis (27)	IGAS	25,764 (10,619/15,145)	European	NA	1.07
Rheumatoid arthritis (28)	GWAS by Okada et al.	58,284 (14,361/43,923)	European	1.26	1.10
Systemic lupus erythematosus (29)	GWAS by Bentham et al.	14,267 (5,201/9,066)	European	1.52	1.03
Psoriasis (30)	GWAS by Tsoi et al.	33,394 (10,588/22,806)	European	NA	1.05
Vitiligo (31)	GWAS by Jin et al.	44,266 (4,680/39,586)	European	1.39	1.07

IPDGC, International Parkinson’s Disease Genomics Consortium; SGPD, Systems genomics of Parkinson’s disease consortium; IMSGC, International Multiple Sclerosis Genetics Consortium; IGAS, International Genetics of Ankylosing Spondylitis Consortium; GWAS, genome-wide association study; Forward min OR, minimum detectable odds ratios in forward MR with statistical power of 80%; Reverse min OR, minimum detectable odds ratios in reverse MR with statistical power of 80%; NA, not available.

**Figure 1:**
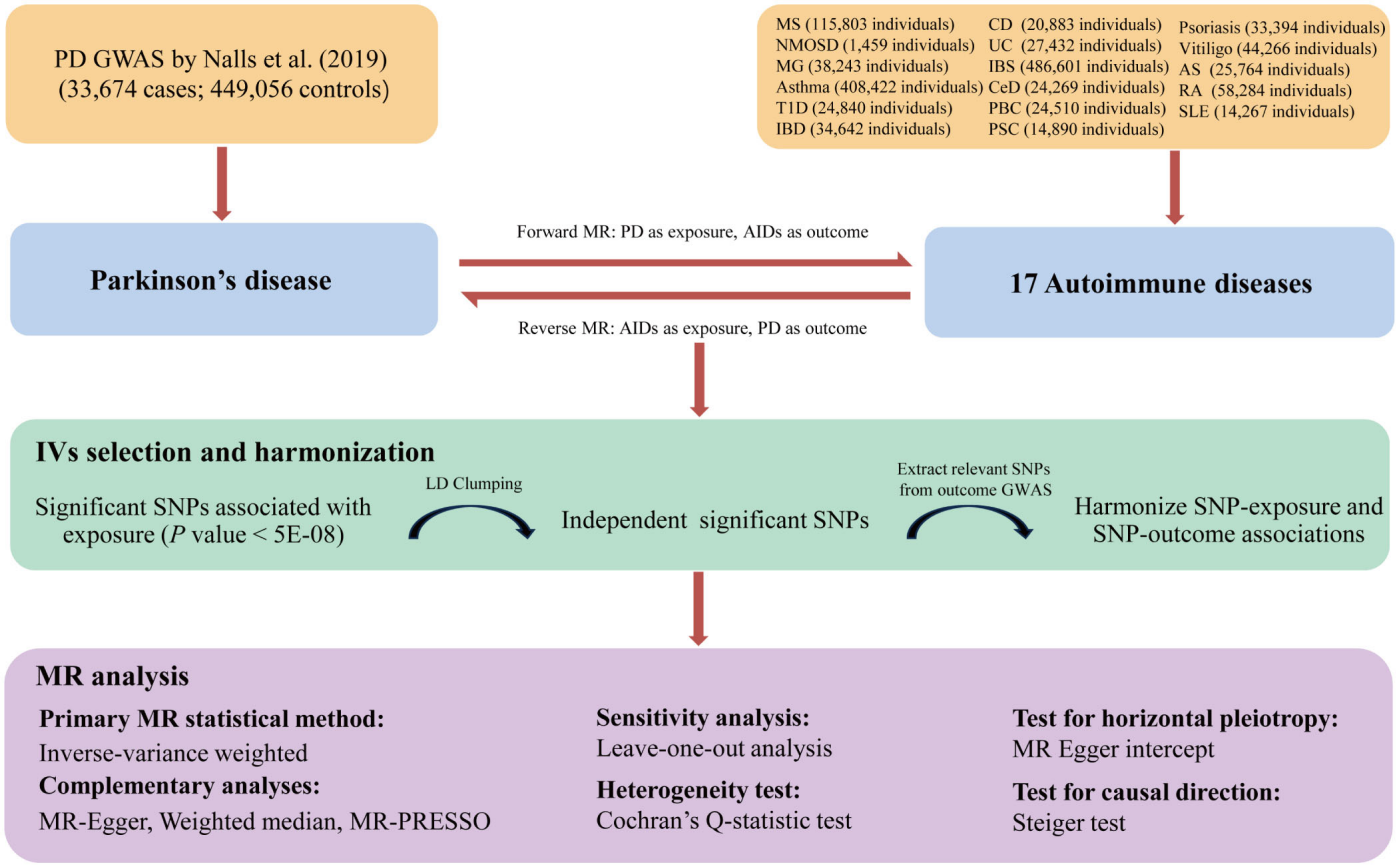
The flowchart of the study design and procedures of the bidirectional Mendelian randomization analysis between PD and AIDs. PD, Parkinson’s disease; AIDs, autoimmune diseases; MS, multiple sclerosis; NMOSD, neuromyelitis optica spectrum disorder; MG, myasthenia gravis; IBD, inflammatory bowel disease; CD, Crohn’s disease; UC, ulcerative colitis; IBS, irritable bowel syndrome; CeD, celiac disease; PBC, primary biliary cirrhosis; PSC, primary sclerosing cholangitis; T1D, type 1 diabetes; AS, ankylosing spondylitis; RA, rheumatoid arthritis; SLE, systemic lupus erythematosus; IV, instrumental variable; SNP, single nucleotide polymorphism; LD, linkage disequilibrium; MR-PRESSO, Mendelian randomization pleiotropy residual sum and outlier.

The overall design of this bidirectional MR study is presented in **Figure 1**. First, we derived SNPs at genome-wide significance (*P* < 5E-08) from the summary-level GWAS statistics and the variants located in human leukocyte antigen (HLA) region (chr6:27,477,797-34,448,354) were removed (32). Then linkage disequilibrium (LD) tests were performed with a genetic window size of 10 Mb and a threshold of *r²* = 0.001 to ascertain independence between SNPs. In the next step, we extracted relevant SNPs from outcome GWAS, and then harmonized the summary effect estimates for each SNP in both exposure and outcome datasets. If exposure IVs were absent in the outcome datasets, proxy SNP in LD (*r²* > 0.8) would be searched on the basis of the European 1,000 Genomes Project reference panel. Furthermore, the *F* statistic was calculated from the formula *F* = *R²*(N-k-1)/[(1- *R²*)k], for which a value above ten was regarded as an indicator of the strong instrument (N: sample size, k: number of IVs, *R²*: the variance of exposure explained by the selected IVs) (33). The same procedure was performed for the reverse MR studies, as such, we conducted a series of bidirectional MR analyses to infer the causality between PD and AIDs, where PD and AIDs were considered either as exposure or as the outcome. Additionally, MR analyses were performed in accordance with the STROBE-MR guidelines (**Supplementary Table 1**) (34, 35).

### 2.2 MR analysis

The inverse variance weighted (IVW) model, as principal analysis, was conducted to assess potential causal associations between genetically determined PD and the occurrence of AIDs. Besides, we applied Cochran’s Q test in the IVW method for quantifying the potential impact of heterogeneity among causal effects of different SNPs (36). Subsequent leave-one-out analysis (LOO) was adopted to investigate whether any single genetic variant carried a disproportionate contribution to IVW estimates in each MR study.

Since the IVW method assumed that all SNPs incorporated in the analysis were valid instrumental variables, the other sensitivity analysis methods were used as the complement to IVW analysis when a violation of the standard MR assumptions existed (33). Specifically, the weighted median (WM) method generated consistent causal estimates even if half of the SNPs were invalid (37). Besides, the MR-Egger intercept, as part of the MR-Egger analysis, was used to identify the possible directional pleiotropic effects which were described as some IVs affecting outcome through alternative pathways other than via exposure. Similarly, Mendelian Randomization Pleiotropy Residual Sum and Outlier (MR-PRESSO) was an approach that allowed for the identification of horizontal pleiotropic effects and removal of outlier SNPs in multi-instrument summary-level MR testing (38). We further carried out the Steiger test to validate direction of causal relationship of exposure with outcome. Using the mRnd power calculation online tool, we had sufficient statistical power greater than 80% to detect the minimum odds ratio (OR).

### 2.3 Genetic correlation

Although SNPs that are directly related to outcomes was eliminated during the IV selection process, SNPs with no relevance could also influence the occurrence of outcome. MR analysis might bias the estimation of causal effects if we found genetic correlations between the exposure and outcome (39). Therefore, after excluding SNPs within the HLA region due to biased LD structure, linkage disequilibrium score (LDSC) regression was performed to estimate latent genetic overlap between two traits by the regression slope utilizing GWAS summary data. When the *P* > 0.05, it indicated that the associations observed in our MR study were truly causal and were not affected by residual pleiotropy. Otherwise, the MR findings might be doubtful.

### 2.4 Statistical analysis

In particular, to adjust for multiple comparisons in assessing the association between PD and AIDs, we utilized Bonferroni correction method. A statistically significant association was identified when the *P*-value was below the Bonferroni-corrected threshold of 0.004 (0.05/13) for AIDs as outcome, and 0.003 (0.05/17) for AIDs as exposure. Moreover, if the *P*-value was lesser than 0.05 but the Bonferroni-corrected *P*-value exceeded 0.05, it was considered a suggestive indication of a causal association. All statistical analyses were performed with LDSC Version 1.0.1, “TwoSampleMR”, “MendelianRandomization” and “MR-PRESSO” packages (R version 4.2.2).

## 3 Results

### 3.1 Causal effect of PD on AIDs

The minimum detectable causal effect sizes of genetic liability to PD on AIDs ranged from 1.11 to 2.97 at a statistical power of 0.8 (**Table 1**). Overall, the number of genetic variants in the PD dataset that ultimately applied as IVs fluctuated from 75 for SLE to 87 for asthma (76 for RA, 82 for IBD, 83 for CD, 84 for MS, UC, vitiligo, 85 for NMOSD, IBS and 86 for MG, T1D). Regarding these various AIDs, the variance explained by IVs ranged from 1.2 to 1.5%, respectively. Additionally, the F-statistics of IVs were consistently above 74, which was greater than the standard cutoff of 10 (**Table 2**).

**Table 2:** Mendelian randomization estimates for causal effects of Parkinson’s disease on 12 autoimmune disorders.

Outcome	No. of SNPs	R²	F-statistics	Inverse variance weighted			MR Egger			Weighted median		
				OR	CI	P-value	OR	CI	P-value	OR	CI	P-value
MS	84	0.013	75	1.02	0.97-1.08	0.448	0.94	0.83-1.06	0.287	0.99	0.92-1.06	0.724
NMOSD	85	0.013	76	1.15	0.87-1.51	0.334	1.40	0.78-2.50	0.259	1.08	0.69-1.68	0.735
MG	86	0.015	84	0.97	0.87-1.07	0.536	0.85	0.69-1.06	0.149	0.95	0.82-1.11	0.531
Asthma	87	0.015	83	0.98	0.96-1.00	0.100	1.00	0.96-1.04	0.824	0.97	0.95-1.00	0.059
IBD	82	0.013	76	1.04	0.98-1.11	0.182	1.06	0.93-1.21	0.380	1.05	0.97-1.13	0.214
CD	83	0.013	76	1.06	0.97-1.15	0.177	1.03	0.87-1.24	0.710	1.00	0.91-1.11	0.954
UC	84	0.013	76	1.04	0.98-1.12	0.211	1.08	0.94-1.25	0.280	1.07	0.97-1.17	0.178
IBS	85	0.013	76	0.98	0.96-0.99	0.032	0.97	0.92-1.01	0.181	0.95	0.93-0.98	0.003
T1D	86	0.013	76	1.10	1.02-1.19	0.010	1.15	0.98-1.35	0.084	1.10	0.99-1.22	0.050
RA	76	0.012	75	0.96	0.91-1.02	0.203	1.04	0.92-1.17	0.568	1.00	0.93-1.08	1.000
SLE	75	0.012	77	1.02	0.92-1.13	0.675	1.12	0.90-1.40	0.321	1.03	0.89-1.19	0.645
Vitiligo	84	0.013	76	0.94	0.85-1.03	0.177	0.83	0.68-1.01	0.059	1.00	0.89-1.13	1.000

MR, Mendelian Randomization; SNP, single-nucleotide polymorphism; OR, odds ratio; CI, confidence interval; MS, multiple sclerosis; NMOSD, neuromyelitis optica spectrum disorder; MG, myasthenia gravis; IBD, inflammatory bowel disease; CD, Crohn’s disease; UC, ulcerative colitis; IBS, irritable bowel syndrome; T1D, type 1 diabetes; RA, rheumatoid arthritis; SLE, systemic lupus erythematosus.

**Figure 2:**
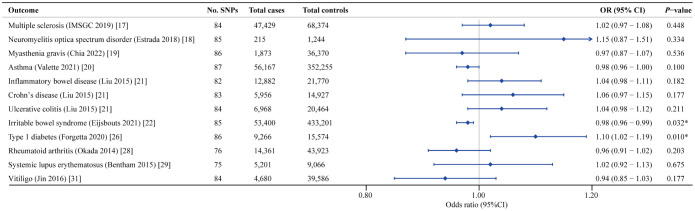
Forest plot of the causal associations between genetically predicted PD and the risk of 12 AIDs.

**Figure 3:**
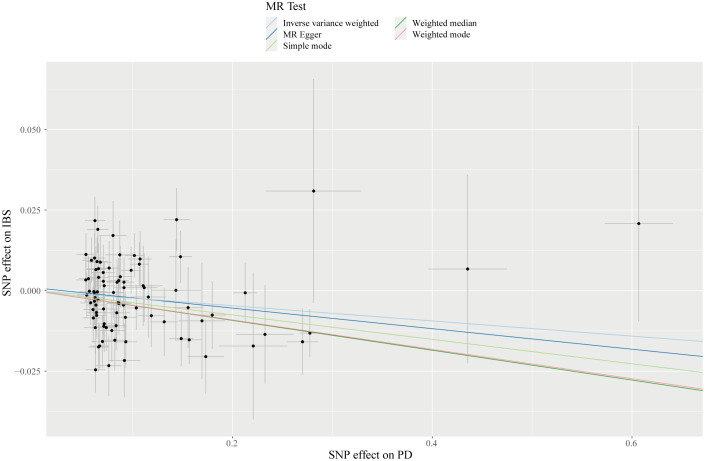
Scatterplot of genetic association with PD against the genetic association with IBS risk. Each black dot indicates an SNP, plotted by the estimate of SNP on PD and the estimate of SNP on IBS risk with standard error bars. The slope of the line represents the causal relationship, and each method has a different line.

According to the IVW estimator, we observed that a higher genetic predisposition to PD was associated with decreased risk of IBS (OR = 0.98; 95% CI: 0.96-0.99; *P* = 0.032), which was corroborated by the weighted median approach (OR = 0.95; 95% CI: 0.93-0.98; *P* = 0.003) (**Table 2**) (**Figures 2**, **3**). Besides, the estimates of the MR-Egger regression analysis showed a directionally consistent but insignificant result (OR = 0.97; 95% CI: 0.92-1.01; *P* = 0.181). Although moderate heterogeneity among chosen SNPs was identified by Cochran’s Q statistics (Q = 123.66; *P* = 0.003), the results of LOO analysis reported that the causal association between PD and IBS was not driven by any individual IV. No horizontal pleiotropic effect could be found due to the MR Egger intercept that was significantly close to zero (*P* = 0.690). Additionally, MR-PRESSO analyses did not detect any outliers, and the Steiger test revealed the causal directions were true, indicating that our MR results were reliable and robust (**Supplementary Table 2**). Furthermore, the results of LDSC analysis provided weak evidence of genetic relevance between PD and IBS (r_g_ = 0.072; se = 0.045; *P* = 0.110), implying the shared genetic components could not confound the causal estimates.

**Figure 4:**
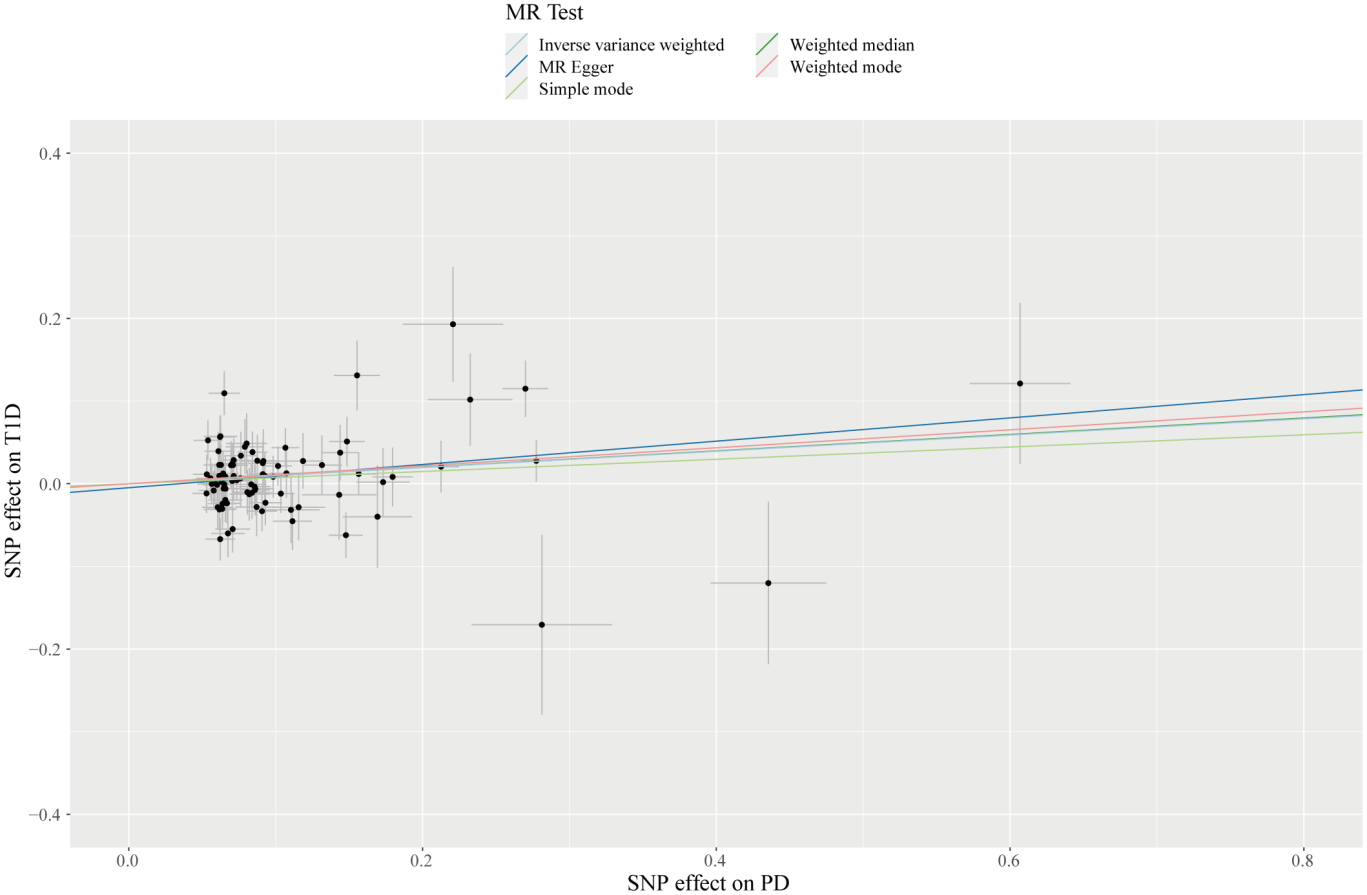
Scatterplot of genetic association with PD against the genetic association with T1D risk. Each black dot indicates an SNP, plotted by the estimate of SNP on PD and the estimate of SNP on IBS risk with standard error bars. The slope of the line represents the causal relationship, and each method has a different line.

Concerning the causal association between PD and T1D, our study suggested a positive relationship of genetically predicted PD on the incidence of T1D (OR = 1.10; 95% CI: 1.02-1.19; *P* = 0.010). Albeit of lower precision, the risk estimates given by the weighted median (OR = 1.10; 95% CI: 0.99-1.22; *P* = 0.050) and MR-Egger method (OR = 1.15; 95% CI: 0.98-1.35; *P* = 0.084) demonstrated a similar causal trend with IVW finding (**Table 2**) (**Figures 2**, **4**). A degree of heterogeneity among SNPs for T1D was detected in the Cochran’s Q test (Q = 129.84; *P* = 0.001), however, by utilizing the leave-one-out approach, we discovered that the causal effect estimates were not influenced by a sole outlier SNP. MR-Egger intercept showed no evidence suggesting significant deviation from zero (*P* = 0.549), which implied the absence of horizontal pleiotropy. Besides, after correction for one outlier variant, the causal effect of PD on T1D remained significant with an OR of 1.09 in the MR-PRESSO test (95% CI: 1.02-1.17; *P* = 0.015). Moreover, the results of the Steiger test showed that the causal pathway was in the direction from PD to T1D (**Supplementary Table 2**). In addition, the genetic correlation results indicated an absence of polygenic pleiotropy between PD and T1D (r_g_ = 0.041; se = 0.073; *P* = 0.578), supporting the issue that pleiotropic IVs unlikely exist in our MR analysis.

As for the remaining AIDs, we found that genetic susceptibility to PD was not relevant to MS, NMOSD, MG, asthma, IBD, CD, UC, RA, SLE, and vitiligo in the IVW as well as subsequent sensitivity analyses (**Table 2**) (**Supplementary Figures 1**, **2**). Moreover, MR analyses of PD on several autoimmune disorders showed low to moderate heterogeneity of used instruments, while MR-Egger regression revealed a lack of horizontal pleiotropic effect (all intercept *P* values were more than 0.05). Although 1, 1, 2, 1, 1, 2, 1 and 1 outliers were depicted by the MR-PRESSO method in the casual estimate of PD on MS, asthma, IBD, CD, UC, RA, SLE and vitiligo, the results of our MR studies did not reflect any significant changes. The MR Steiger test pointed out that all causal impacts except NMOSD were in the intended direction (**Supplementary Table 2**).

### 3.2 Causal effect of AIDs on PD

Our study had 80% power to detect the causal estimates of genetic predisposition to AIDS on PD, whose ORs ranged from 1.03 to 1.51 (**Table 1**). In addition, a total of 4, 6, 12, 20, 30, 32, 32, 33, 34, 37, 38, 48, 57, 60, 62 and 70 SNPs for detecting causal relationship of MG, IBS, PSC, AS, CeD, UC, T1D, RA, vitiligo, SLE, PBC, CD, IBD, MS, psoriasis as well as asthma on PD risk were enrolled in our reverse-direction MR analyses, respectively. Genetic instruments from the GWAS of autoimmune disorders collectively accounted for 0.1 to 22.3% of the variance. Besides, instrumental variables for each AID had F-statistics from 32 to 123, eliminating potential weak instrument bias.

**Figure 5:**
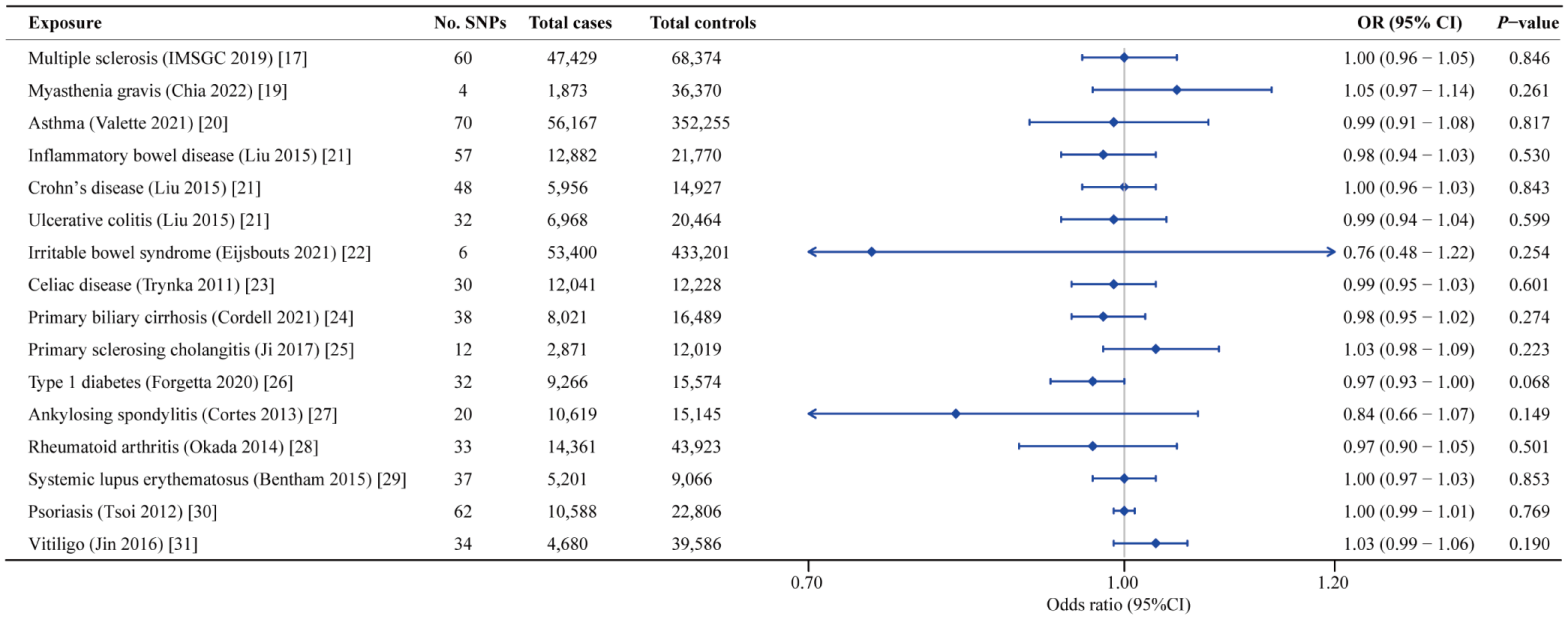
Forest plot of the causal associations between genetically predicted 16 AIDs and the risk of PD.

When tested with IVW analyses, no causal association was observed between genetically determined MS (OR = 1.00; 95% CI: 0.96-1.05; *P* = 0.846), MG (OR = 1.05; 95% CI: 0.97-1.14; *P* = 0.261), asthma (OR = 0.99; 95% CI: 0.91-1.08; *P* = 0.817), IBD (OR = 0.98; 95% CI: 0.94-1.03; *P* = 0.530), CD (OR = 1.00; 95% CI: 0.96-1.03; *P* = 0.843), UC (OR = 0.99; 95% CI: 0.94-1.04; *P* = 0.599), IBS (OR = 0.76; 95% CI: 0.48-1.22; *P* = 0.254), CeD (OR = 0.99; 95% CI: 0.95-1.03; *P* = 0.601), PBC (OR = 0.98; 95% CI: 0.95-1.02; *P* = 0.274), PSC (OR = 1.03; 95% CI: 0.98-1.09; *P* = 0.223), T1D (OR = 0.97; 95% CI: 0.93-1.00; *P* = 0.068), AS (OR = 0.84; 95% CI: 0.66-1.07; *P* = 0.149), RA (OR = 0.97; 95% CI: 0.90-1.05; *P* = 0.501), SLE (OR = 1.00; 95% CI: 0.97-1.03; *P* = 0.853), psoriasis (OR = 1.00; 95% CI: 0.99-1.01; *P* = 0.769) and vitiligo (OR = 1.03; 95% CI: 0.99-1.06; *P* = 0.190) and the risk of PD (**Table 3**) (**Figure 5**; **Supplementary Figures 3**, **4**). For the supplementary analyses we performed, the MR-Egger and weighed median estimator yielded similar results despite some with reduced statistical power. We detected slight heterogeneity in the principal analyses of causal relationships of IBD, CD, UC, PBC, RA, SLE and vitiligo on PD. As the MR-Egger intercept test corresponded to the statistics with a *P*-value > 0.05, it meant that our MR findings, except for IBD as exposure, were not affected by genetic pleiotropy. Additionally, the MR-PRESSO indicated that the causal estimates of IBD, CD, PBC, RA and vitiligo with PD were retained after pruning outliers. Lastly, our Steiger test provided evidence of significant inferred causal direction between 16 AIDs and PD (**Supplementary Table 3**).

**Table 3:** Mendelian randomization estimates for causal effects of 16 autoimmune disorders on Parkinson’s disease.

Exposure	No. of SNPs	R²	F-statistics	Inverse variance weighted			MR Egger			Weighted median		
				OR	CI	P-value	OR	CI	P-value	OR	CI	P-value
MS	60	0.023	46	1.00	0.96-1.05	0.846	1.04	0.90-1.20	0.573	0.99	0.93-1.05	0.761
MG	4	0.004	39	1.05	0.97-1.14	0.261	0.87	0.49-1.54	0.674	1.05	0.95-1.16	0.328
Asthma	70	0.012	70	0.99	0.91-1.08	0.817	1.02	0.80-1.28	0.897	0.96	0.85-1.09	0.550
IBD	57	0.094	63	0.98	0.94-1.03	0.530	1.23	1.01-1.51	0.047	1.01	0.95-1.06	0.917
CD	48	0.134	67	1.00	0.96-1.03	0.843	1.11	0.99-1.26	0.087	1.01	0.97-1.05	0.709
UC	32	0.057	52	0.99	0.94-1.04	0.599	1.05	0.83-1.34	0.690	1.00	0.94-1.06	0.945
IBS	6	0.001	32	0.76	0.48-1.22	0.254	0.03	0.00-16.10	0.339	0.76	0.45-1.31	0.313
CeD	30	0.132	123	0.99	0.95-1.03	0.601	1.02	0.95-1.09	0.639	1.03	0.97-1.09	0.300
PBC	38	0.122	91	0.98	0.95-1.02	0.274	0.97	0.88-1.07	0.527	0.99	0.95-1.03	0.618
PSC	12	0.032	41	1.03	0.98-1.09	0.223	0.98	0.79-1.22	0.879	1.01	0.94-1.07	0.871
T1D	32	0.072	60	0.97	0.93-1.00	0.068	0.92	0.85-0.99	0.026	0.93	0.88-0.99	0.012
AS	20	0.048	63	0.84	0.66-1.07	0.149	1.12	0.53-2.37	0.775	0.85	0.61-1.19	0.346
RA	33	0.028	50	0.97	0.90-1.05	0.501	1.10	0.80-1.52	0.548	0.98	0.90-1.07	0.716
SLE	37	0.223	111	1.00	0.97-1.03	0.853	0.97	0.91-1.04	0.467	1.00	0.96-1.04	0.944
Psoriasis	62	0.100	60	1.00	0.99-1.01	0.769	1.00	0.99-1.01	0.844	1.00	0.99-1.01	0.599
Vitiligo	34	0.047	64	1.03	0.99-1.06	0.190	1.06	0.93-1.20	0.398	1.03	0.98-1.07	0.235

MR, Mendelian Randomization; SNP, single-nucleotide polymorphism; OR, odds ratio; CI, confidence interval; MS, multiple sclerosis; MG, myasthenia gravis; IBD, inflammatory bowel disease; CD, Crohn’s disease; UC, ulcerative colitis; IBS, irritable bowel syndrome; CeD, celiac disease; PBC, primary biliary cirrhosis; PSC, primary sclerosing cholangitis; T1D, type 1 diabetes; AS, ankylosing spondylitis; RA, rheumatoid arthritis; SLE, systemic lupus erythematosus.

## 4 Discussion

Nowadays, Parkinson’s disease and autoimmune disorders have imposed a huge health and economic burden on individuals and society around the world. However, the profound understanding of the potential genetic mechanism and pathophysiological link remains limited, which leads to an absence of effective prevention and treatment strategies. Although a large body of epidemiological researches emerged to investigate the correlation between PD and AIDs, these observational studies were vulnerable to reverse causation and residual confounding bias. Thus, in the present analysis, we conducted a bidirectional two-sample MR study for comprehensively exploring the mutual causal associations between genetically determined PD and AIDs by leveraging publicly available summary statistics from large GWAS datasets. Based on the MR results after correction for multiple sensitivity tests, our study provided evidence that PD had a protective influence on IBS risk, whereas a possible detrimental link was reported between genetic predisposition to PD and T1D susceptibility. Additionally, in the reverse MR analyses, we found no indication to support the causal effect of AIDs on PD occurrence.

Our MR results supported that individuals with high genetic risk for PD suggestively have a lower susceptibility to developing IBS. However, a previous register-based observational study from the Swedish Patient Register showed that IBS diagnosis was related to an increased risk of PD (40). Nonetheless, as an observational case-control study, it was inevitably vulnerable to confounding factors and encountered challenges in accurately determining the diagnostic sequence of PD and IBS. Unlike findings from the observational investigations, our MR analysis improved the reliability of causal inference with the use of genetic variants as IVs, avoiding systemic bias derived from modifiable confounders and reverse causation. Furthermore, the following biological mechanisms could account for the protective effect of PD against IBS risk. The altered gastrointestinal microbiota in PD patients might be a major factor in the pathophysiology of IBS. On the one hand, through the application of high-throughput 16S rRNA sequencing, gut microbiome dysbiosis was commonly detected in PD patients, revealing a higher relative abundance of Lactobacillus and Bifidobacterium (41). Furthermore, O’Mahony et al. observed that Lactobacillus and Bifidobacterium could participate in peripheral immunity through up-regulating IL-10 and down-regulating IL-12 levels in peripheral blood mononuclear cells (42). On the other hand, a prior meta-analysis composed of 22 studies by Romano et al. demonstrated the microbiological pattern of reduced Lachnospiraceae genus content in individuals with PD (43). Moreover, flagellin from Lachnospiraceae was reported to activate innate and adaptive immunity, eliciting anxiety-like behavior, abnormal gastrointestinal transit, and intestinal barrier dysfunction in a humanized mouse model of IBS (44, 45). In addition to gut microbiome dysbiosis, another possible mechanism linking PD to IBS was dysregulation of neurotransmitters and neurotrophic factors. It is well-known that deficits in dopamine and 5-HT signaling are implicated in the initiation and progression of PD (46, 47), but as for IBS, reduced levels of these neurotransmitters could mitigate pathological symptoms like diarrhea, abdominal pain and discomfort and stabilize intestinal permeability (48–51). Additionally, although decreased netrin-1 concentrations in the brain and gut of PD patients resulted in significant loss of motor function, it provided an obvious alleviation effect on visceral hypersensitivity, a core characteristic in approximately half of patients with IBS (52, 53). Thus, future large-scale prospective cohort studies are urgently required to verify this causal relationship.

Regarding the causal effect of PD on T1D occurrence, we found that those who were genetically predisposed to PD exhibited a 10% increased incidence of experiencing T1D. This causal association could be explained by perturbation of the immune system and inflammatory response. It was reported that PD patients were afflicted with persistent neuroinflammation, which disrupted BBB integrity and resulted in BBB leakage (54, 55). Following the changes in BBB permeability, more pro-inflammatory cytokines (TNF-α, IL-1β, etc.) yielded by overactivated neurotoxic microglia had the opportunity to enter the systemic circulation (9, 56). Meanwhile, in a PD rat model induced by injection of 1-methyl-4-phenyl-1,2,3,6-tetrahydropyridine (MPTP), nitrated alpha-synuclein (N-α-synuclein) was detected to break immunological tolerance and activate T cells into Th1 and Th17 subsets of T helper cells (56–58). Moreover, increased activation of natural killer cells, B lymphocytes and monocytes was also found in the peripheral blood samples of PD patients compared to healthy controls using flow cytometric analyses (59–61). Thus, accompanied by elevated concentrations of pro-inflammatory mediators, including IFN-γ, TNF-α, IL-6, IL-17 and activated peripheral immune cells, an enhanced immune response might mistakenly lead to attack and destroy the insulin-producing beta cells in pancreatic islets (62). Additionally, Li et al. identified that in A53 transgenic (A53T) PD mice, the expression levels of cytotoxic-T-lymphocyte-antigen-4 (CTLA-4) and inducible costimulator (ICOS) were markedly reduced in T regulatory lymphocytes (Tregs), a critical player in maintaining immune homeostasis (63). Similarly, Tregs were also found to exhibit functional defects in PD patients, implying an abolished suppressive effect on autoimmune response and a contributory factor for the emergence of T1D (64). Meanwhile, in the reverse direction MR analysis, we failed to observe a causal effect of T1D on the susceptibility to PD. However, a previous MR analysis observed that T1D might play a protective role against PD with an OR of 0.97 (95% CI: 0.94-0.99; *P* = 0.039) (65). The primary factors contributing to the inconsistency could stem from the utilization of disparate GWAS datasets and selection strategy for instrumental variables. Specifically, the T1D GWAS adopted by Senkevich et al. only encompassed 714,850 SNPs, half of which lacked summary statistics (beta, SE, *p*-value), whereas the GWAS data utilized in our MR analysis included over 1.2 million SNPs with complete data. The evident increase in the number of available SNPs guaranteed more comprehensive genome coverage, thereby minimizing the possibility of falsely detecting genetic variants associated with T1D and PD. Additionally, we applied stringent criteria to choose instrumental variables. The SNPs related to more than one autoimmune disease were excluded to mitigate pleiotropic effects. Interestingly, after adding this rigorous screening procedure, we re-analyzed the GWAS data used in previous MR study by Senkevich et al. and the results demonstrated that the protective effect of T1D on PD occurrence disappeared (data not shown).

Concerning the relationships between AIDs and PD, a comprehensive meta-analysis by Li et al. showed that aging individuals diagnosed with IBD, CD and UC had a 1.24, 1.30 and 1.31-fold increased likelihood of developing PD than the healthy controls (66). Likewise, Yeh et al. found that in a population−based cohort study of 6,440 AS patients and 25,760 matched controls, the incidence of PD was significantly higher in the AS cohort than in reference individuals (67). However, compared with previous findings in observational studies, our reverse MR analyses showed that genetically determined AIDs were not causally associated with PD risk. This paradox might be ascribed to the reasons that observational studies were prone to bias owing to the limited sample size, inevitable confounding factors and reverse causation. Therefore, we carried out these two-sample MR analyses and obtained reliable and robust results demonstrating no causality of 16 AIDs on PD susceptibility.

Our study benefits from several noteworthy strengths. First, to the best of our knowledge, our MR study is the first to systematically elucidate the bidirectional causative links between PD and AIDs, which yields instructive insight for the management of autoimmune disorders in PD patients as well as the prevention strategies for PD problems in individuals with AIDs. An additional advantage of this MR analysis is that the largest and latest GWAS datasets for AIDs and PD were implemented, providing strong statistical power and precise effect estimates to explore the causal associations. Additionally, except for IVW analysis, we also employed exhaustive complementary analyses containing MR Egger regression, weighted median and MR-PRESSO. The directionally consistent results observed from multiple MR techniques ensured the robustness of our MR findings. Lastly, since genetic variants within the HLA region (chr6:27,477,797-34,448,354) were in strong LD and had high pleiotropic effects on a variety of autoimmune disorders, our study excluded these variants to help us avoid possible bias in MR instruments and more accurately identify causality. However, some potential limitations should also be acknowledged in our MR analyses. First, although we selected the largest GWAS datasets to date for various AIDs, the screened IVs for AIDs such as NMOSD remained somewhat underrepresented, making it difficult to obtain sufficient genetic instruments for bidirectional MR studies. As such, larger-scale GWAS should be updated in the future to boost the statistical power for investigating the causal associations between PD and AIDs. Second, it should be noted that the majority of GWAS participants were of European ancestry. Thus, our viewpoint on causal inferences between PD and AIDs should be applied with caution to other racial and ethnic populations. Last but not least, although our MR studies support the evidence for causal relationships of genetically determined PD with IBS and T1D occurrence, these findings and underlying pathophysiological mechanisms need to be further confirmed by cross-sectional, longitudinal cohort studies and molecular biology experiments *in vivo* and *in vitro*.

## 5 Conclusion

In conclusion, we performed this bidirectional 2-sample MR analysis for the first time to explore the causal associations between PD and AIDs. Our forward MR studies identified that genetically predicted PD played a dual role in AIDs, not only protecting against IBS but also exerting a detrimental effect on T1D. Simultaneously, in the reverse direction, we found a lack of evidence for the causal effect of AIDs on PD risk. We hope our study could provide new insights into the causal relationships between PD and AIDs, thereby contributing to the development of clinical strategies for autoimmune disorders in PD individuals.

## Data availability statement

The datasets presented in this study can be found in online repositories. The names of the repository/repositories and accession number(s) can be found in the article/**Supplementary Material**.

## Author contributions

JY: Data curation, Formal analysis, Investigation, Software, Visualization, Writing – original draft. WL: Data curation, Formal analysis, Writing – original draft. YM: Writing – original draft, Visualization. HS: Writing – original draft. CM: Writing – original draft, Data curation. QW: Writing – original draft. CH: Writing – original draft. JZ: Conceptualization, Data curation, Writing – review & editing. XL: Conceptualization, Funding acquisition, Methodology, Writing – review & editing.

## Supplementary material

Supplementary Figure 1 Scatter plots of MR tests assessing the effect of PD on AIDs. **(A)** PD on MS; **(B)** PD on NMOSD; **(C)** PD on MG; **(D)** PD on Asthma; **(E)** PD on IBD; **(F)** PD on CD; **(G)** PD on UC; **(H)** PD on RA; **(I)** PD on SLE; **(J)** PD on Vitiligo. PD, Parkinson’s disease; AIDs, autoimmune diseases; MS, multiple sclerosis; NMOSD, neuromyelitis optica spectrum disorder; MG, myasthenia gravis; IBD, inflammatory bowel disease; CD, Crohn’s disease; UC, ulcerative colitis; RA, rheumatoid arthritis; SLE, systemic lupus erythematosus.

Supplementary Figure 2 Leave-one-out plots of MR tests assessing the effect of PD on AIDs. **(A)** PD on MS; **(B)** PD on NMOSD; **(C)** PD on MG; **(D)** PD on Asthma; **(E)** PD on IBD; **(F)** PD on CD; **(G)** PD on UC; **(H)** PD on IBS; **(I)** PD on T1D; **(J)** PD on RA; **(K)** PD on SLE; **(L)** PD on Vitiligo. PD, Parkinson’s disease; AIDs, autoimmune diseases; MS, multiple sclerosis; NMOSD, neuromyelitis optica spectrum disorder; MG, myasthenia gravis; IBD, inflammatory bowel disease; CD, Crohn’s disease; UC, ulcerative colitis; IBS, irritable bowel syndrome; T1D, type 1 diabetes; RA, rheumatoid arthritis; SLE, systemic lupus erythematosus.

Supplementary Figure 3 Scatter plots of MR tests assessing the effect of AIDs on PD. **(A)** MS on PD; **(B)** MG on PD; **(C)** Asthma on PD; **(D)** IBD on PD; **(E)** CD on PD; **(F)** UC on PD; **(G)** IBS on PD; **(H)** CeD on PD; **(I)** PBC on PD; **(J)** PSC on PD; **(K)** T1D on PD; **(L)** AS on PD; **(M)** RA on PD; **(N)** SLE on PD; **(O)** Psoriasis on PD; **(P)** Vitiligo on PD. AIDs, autoimmune diseases; PD, Parkinson’s disease; MS, multiple sclerosis; MG, myasthenia gravis; IBD, inflammatory bowel disease; CD, Crohn’s disease; UC, ulcerative colitis; IBS, irritable bowel syndrome; CeD, celiac disease; PBC, primary biliary cirrhosis; PSC, primary sclerosing cholangitis; T1D, type 1 diabetes; AS, ankylosing spondylitis; RA, rheumatoid arthritis; SLE, systemic lupus erythematosus.

Supplementary Figure 4 Leave-one-out plots of MR tests assessing the effect of AIDs on PD. **(A)** MS on PD; **(B)** MG on PD; **(C)** Asthma on PD; **(D)** IBD on PD; **(E)** CD on PD; **(F)** UC on PD; **(G)** IBS on PD; **(H)** CeD on PD; **(I)** PBC on PD; **(J)** PSC on PD; **(K)** T1D on PD; **(L)** AS on PD; **(M)** RA on PD; **(N)** SLE on PD; **(O)** Psoriasis on PD; **(P)** Vitiligo on PD. AIDs, autoimmune diseases; PD, Parkinson’s disease; MS, multiple sclerosis; MG, myasthenia gravis; IBD, inflammatory bowel disease; CD, Crohn’s disease; UC, ulcerative colitis; IBS, irritable bowel syndrome; CeD, celiac disease; PBC, primary biliary cirrhosis; PSC, primary sclerosing cholangitis; T1D, type 1 diabetes; AS, ankylosing spondylitis; RA, rheumatoid arthritis; SLE, systemic lupus erythematosus.
