# Supplementary material for: Investigation of the causal association between Parkinson’s disease and autoimmune disorders: a bidirectional Mendelian randomization study
Source: Front Immunol. 2024 May 7;15:1370831. doi: 10.3389/fimmu.2024.1370831 (PMC11106379; doi:10.3389/fimmu.2024.1370831)
Supplement: Supplementary Figure 1 — Scatter plots of MR tests assessing the effect of PD on AIDs. (A) PD on MS; (B) PD on NMOSD; (C) PD on MG; (D) PD on Asthma; (E) PD on IBD; (F) PD on CD; (G) PD on UC; (H) PD on RA; (I) PD on SLE; (J) PD on Vitiligo. PD, Parkinson’s disease; AIDs, autoimmune diseases; MS, multiple sclerosis; NMOSD, neuromyelitis optica spectrum disorder; MG, myasthenia gravis; IBD, inflammatory bowel disease; CD, Crohn’s disease; UC, ulcerative colitis; RA, rheumatoid arthritis; SLE, systemic lupus erythematosus. [file DataSheet_1.pdf]

Supplementary Figure 1. Scatter plots of MR tests assessing the effect of PD on AIDs.

A

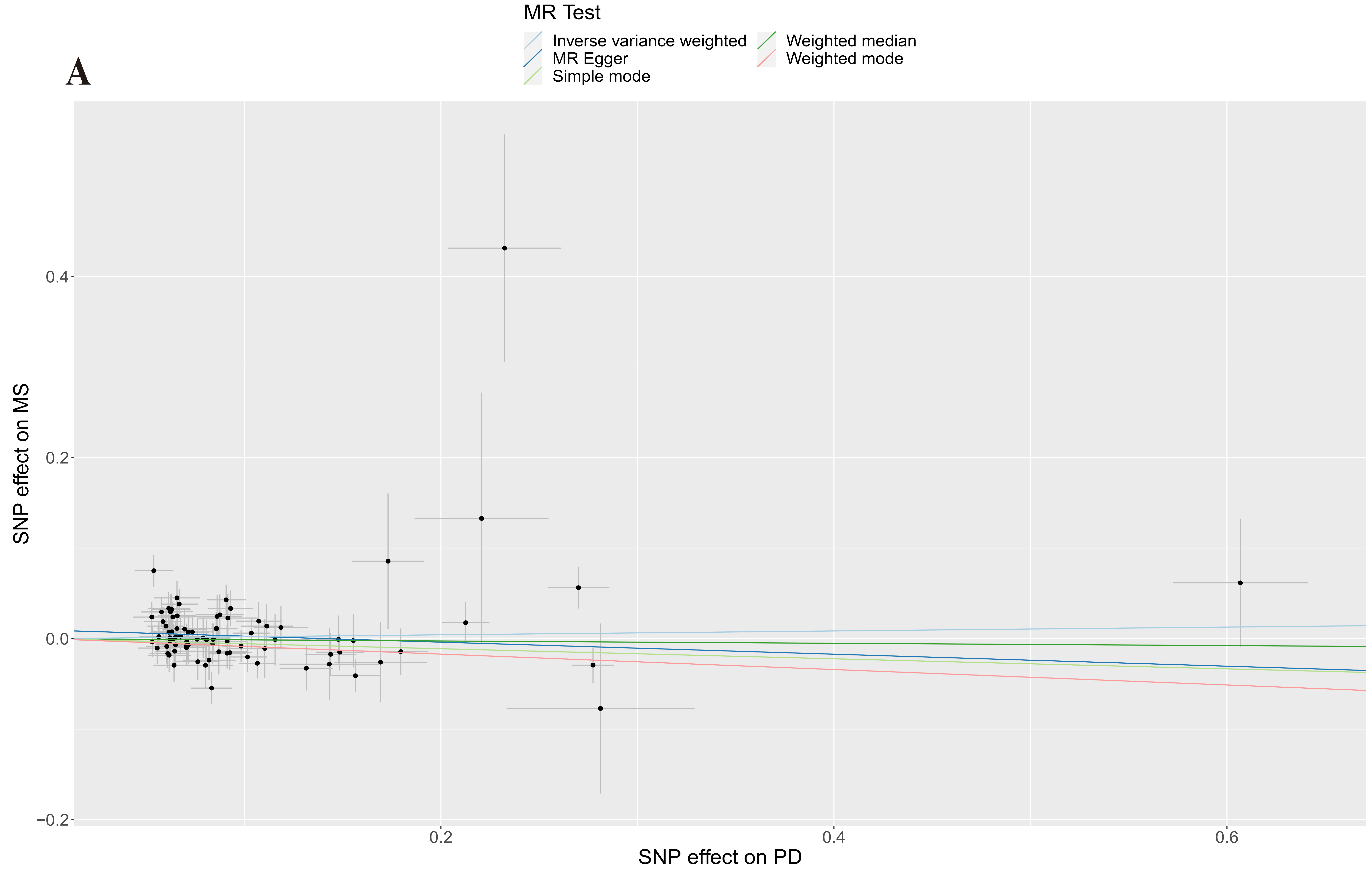

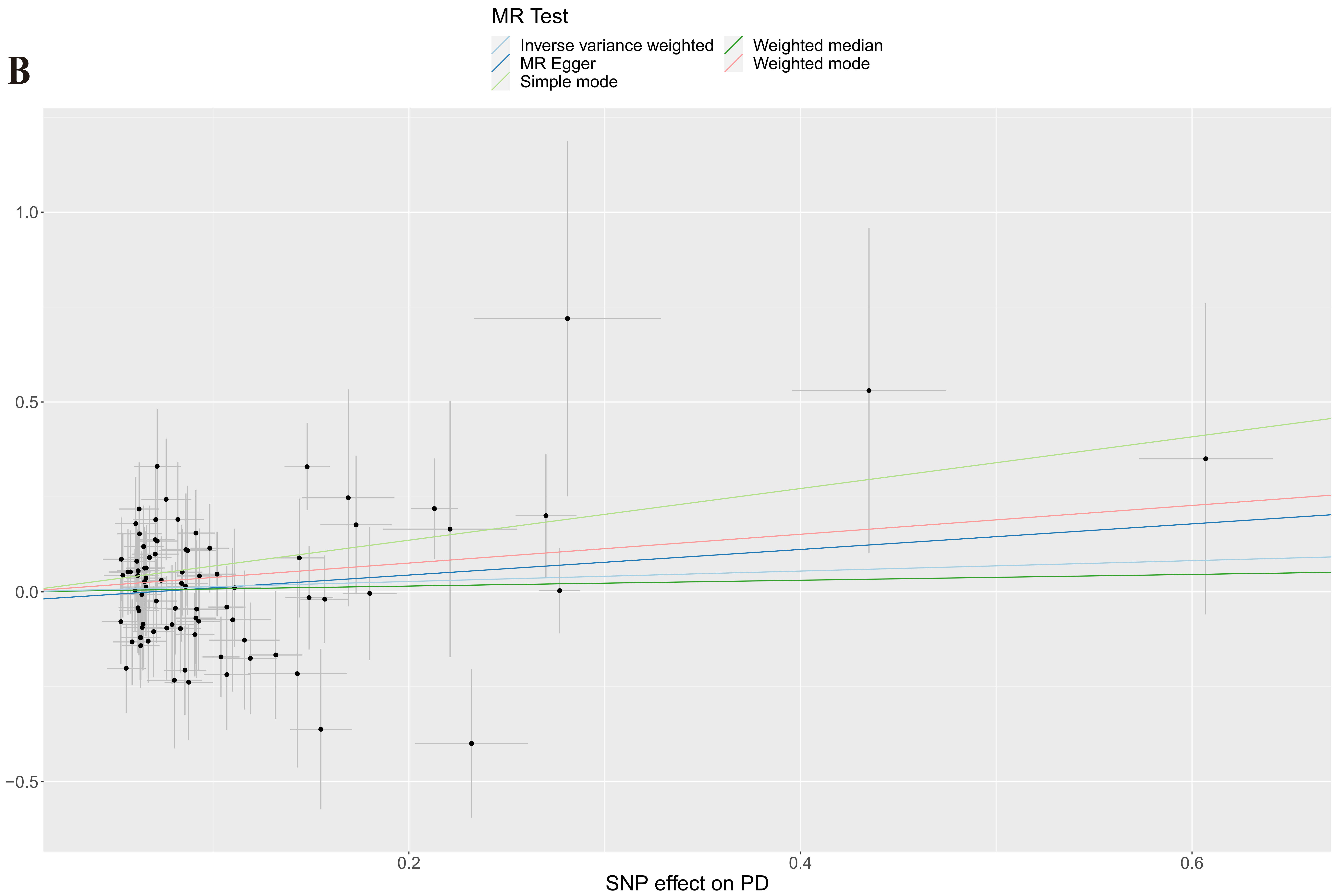

B
MR Test
Inverse variance weighted
MR Egger
Simple mode
Weighted median
Weighted mode
1.0
0.5
0.0
−0.5
SNP effect on NMOSD
0.2
0.4
0.6
SNP effect on PD

C

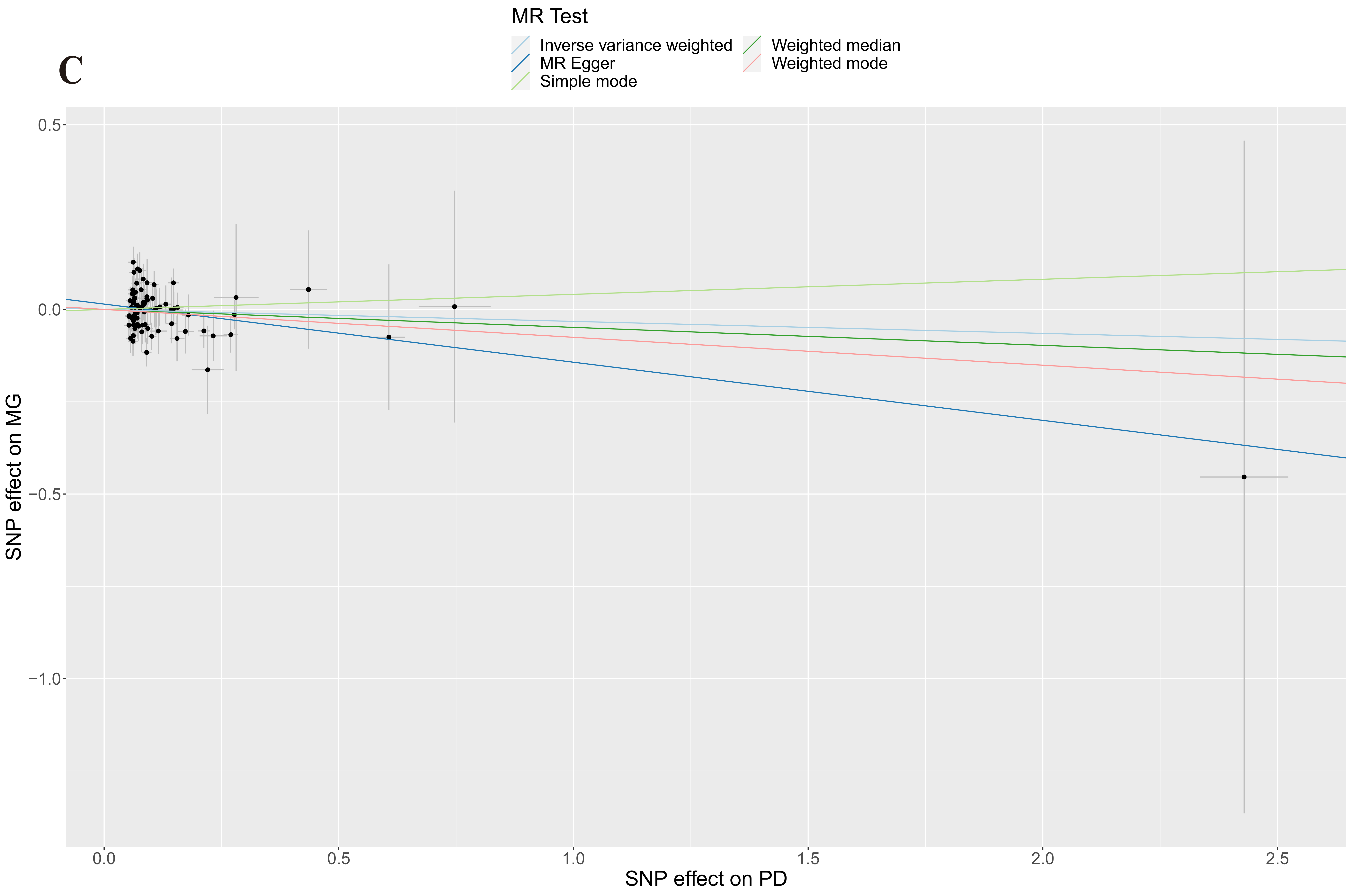

MR Test
Inverse variance weighted
MR Egger
Simple mode
Weighted median
Weighted mode
0.5
0.0
−0.5
−1.0
SNP effect on MG
0.0
0.5
1.0
1.5
2.0
2.5
SNP effect on PD

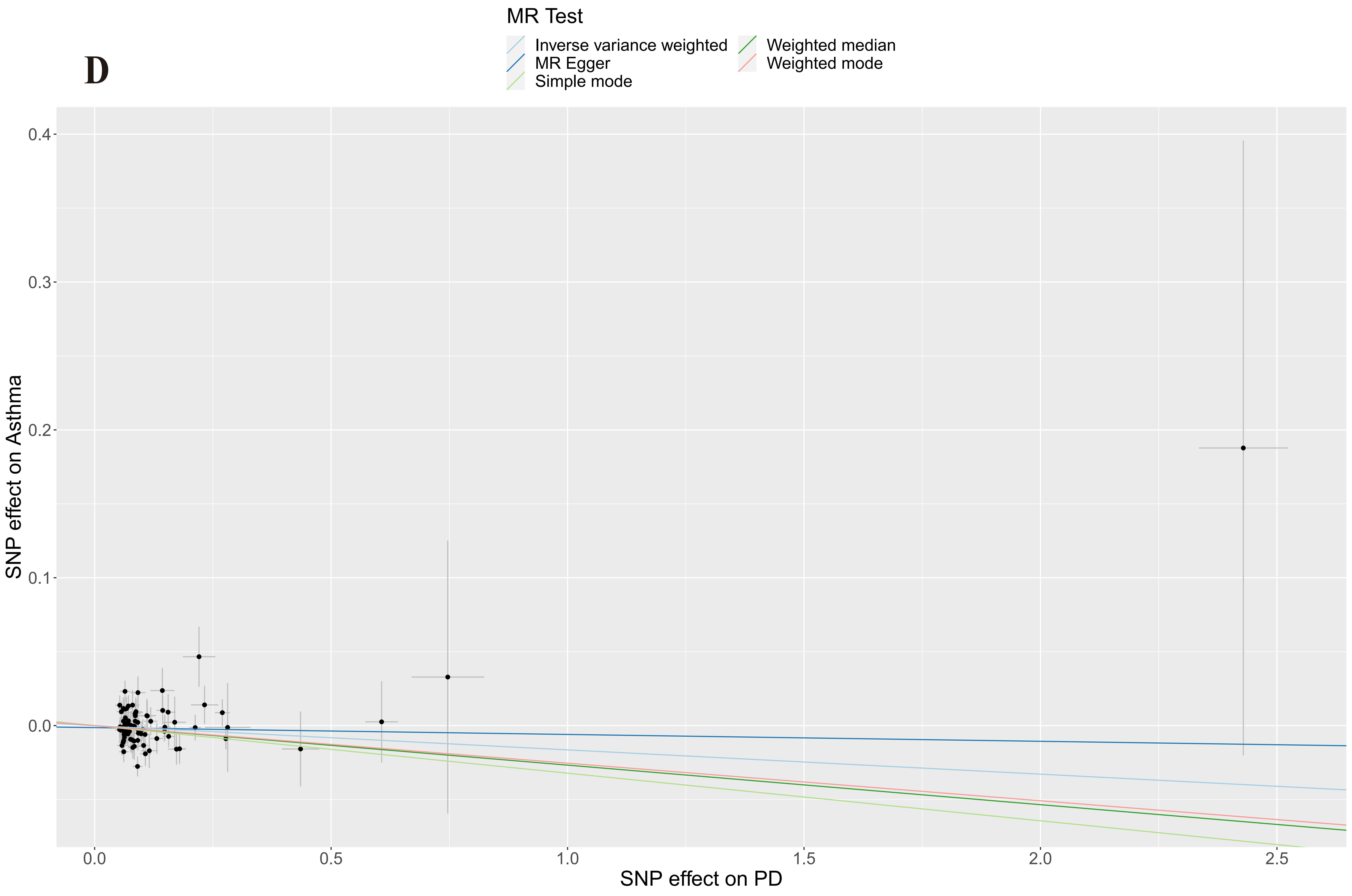
D
MR Test
Inverse variance weighted
MR Egger
Simple mode
Weighted median
Weighted mode
SNP effect on Asthma
0.4
0.3
0.2
0.1
0.0
0.0
0.5
1.0
1.5
2.0
2.5
SNP effect on PD

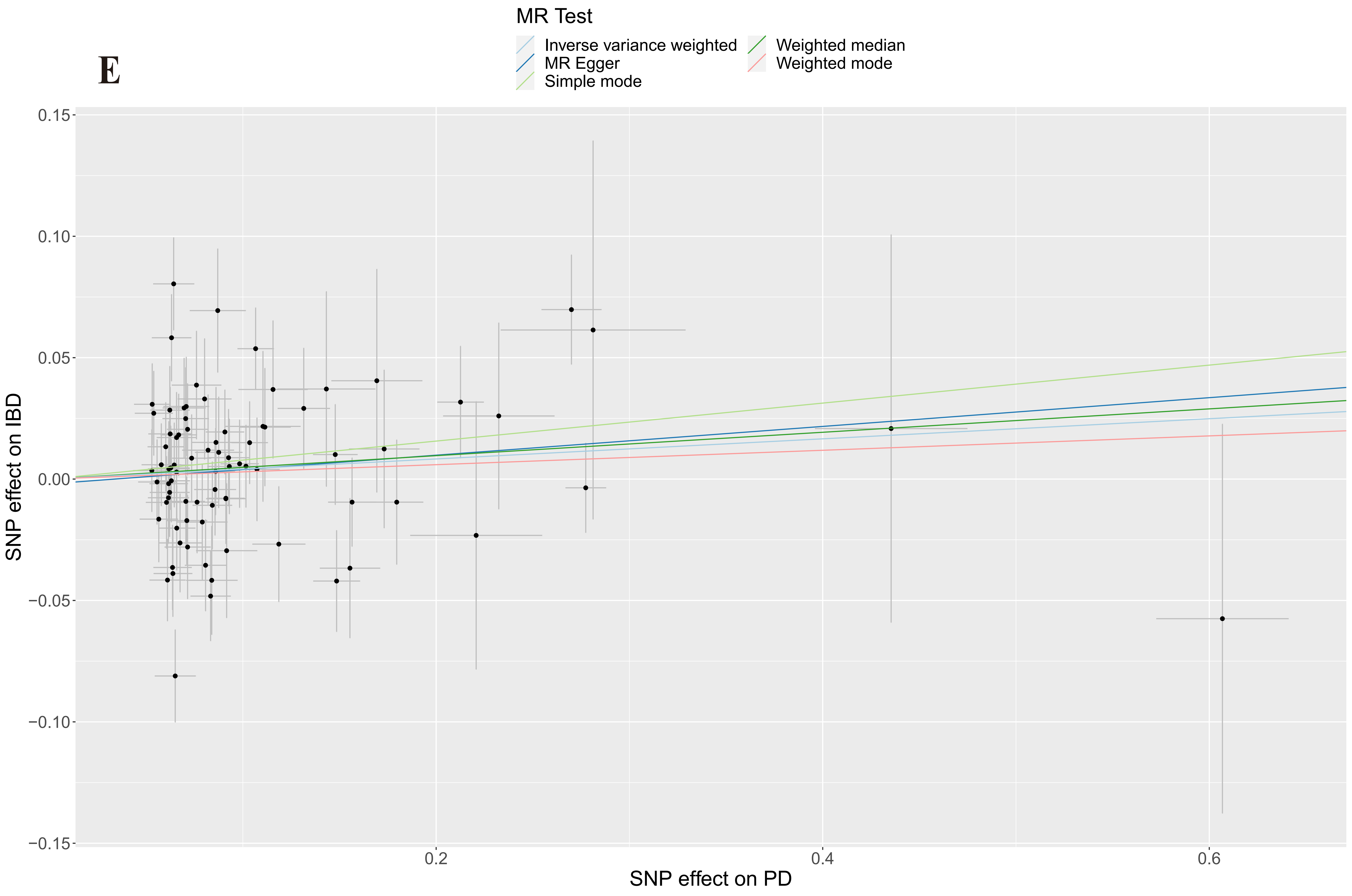

E
MR Test
Inverse variance weighted
MR Egger
Simple mode
Weighted median
Weighted mode
0.15
0.10
0.05
0.00
−0.05
−0.10
−0.15
SNP effect on IBD
0.2
0.4
0.6
SNP effect on PD

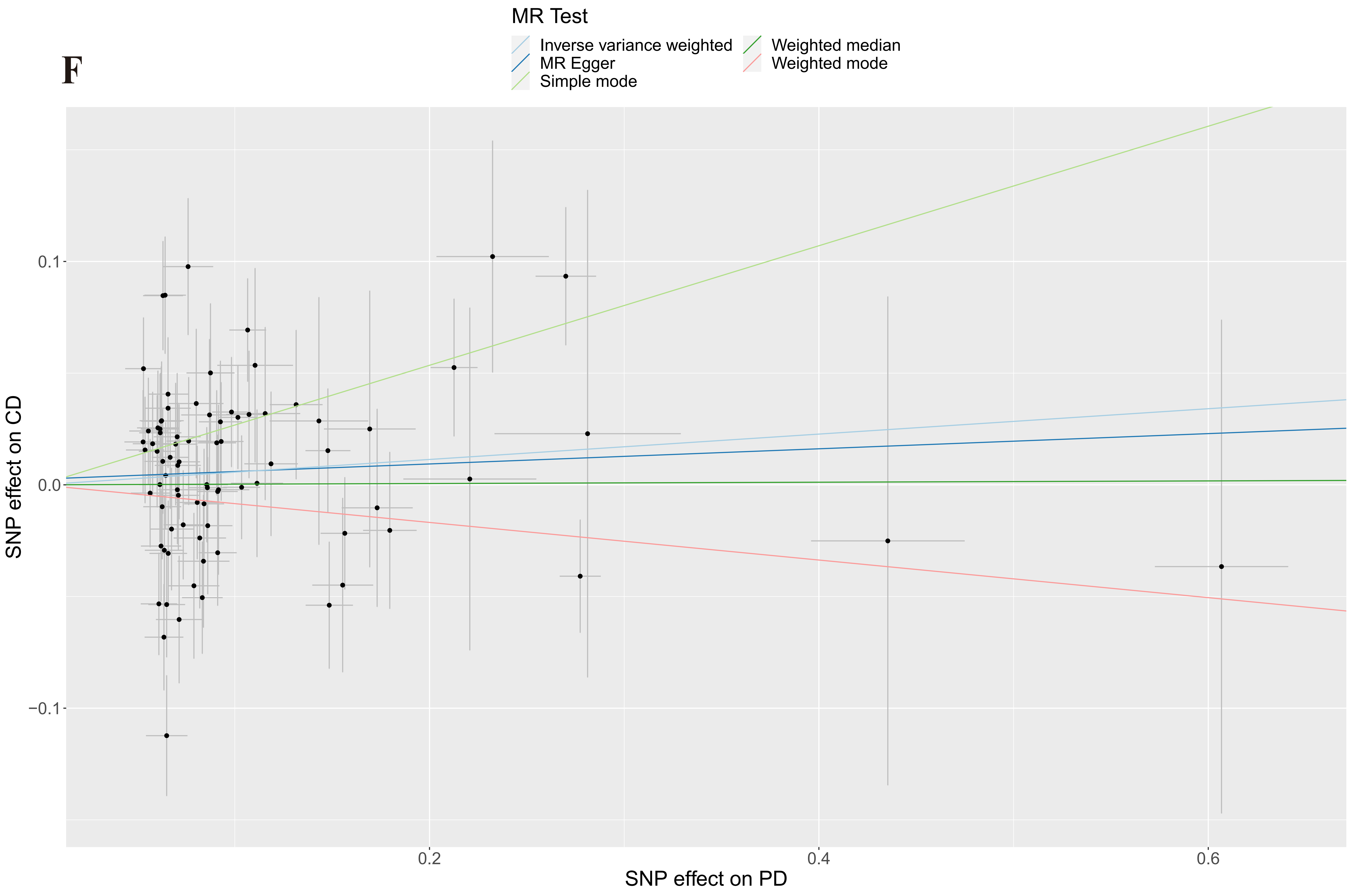
F
MR Test
Inverse variance weighted
MR Egger
Simple mode
Weighted median
Weighted mode
SNP effect on CD
0.1
0.0
−0.1
SNP effect on PD
0.2
0.4
0.6

G

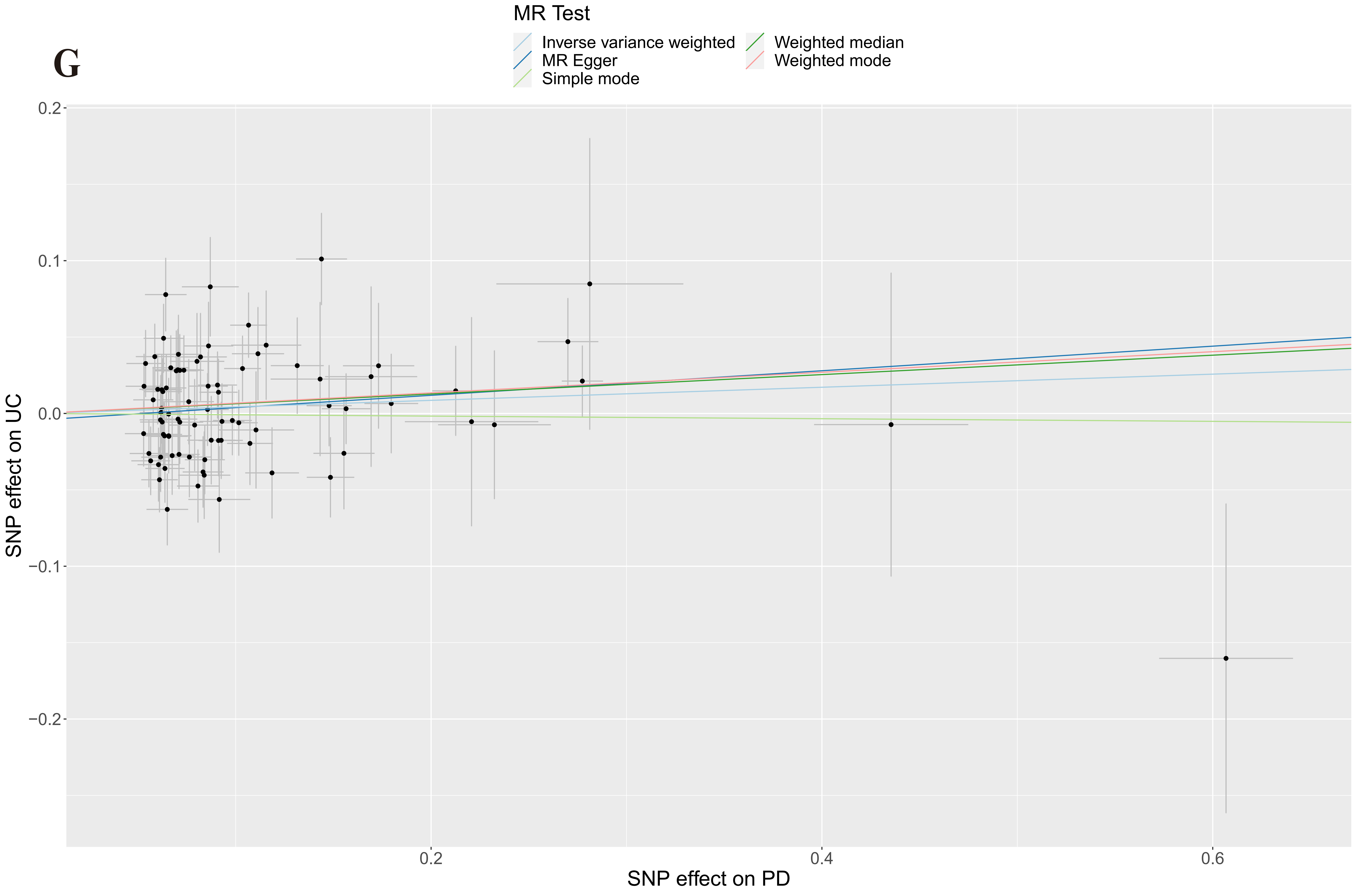

MR Test
Inverse variance weighted
MR Egger
Simple mode
Weighted median
Weighted mode
0.2
0.1
0.0
−0.1
−0.2
SNP effect on UC
0.2
0.4
0.6
SNP effect on PD

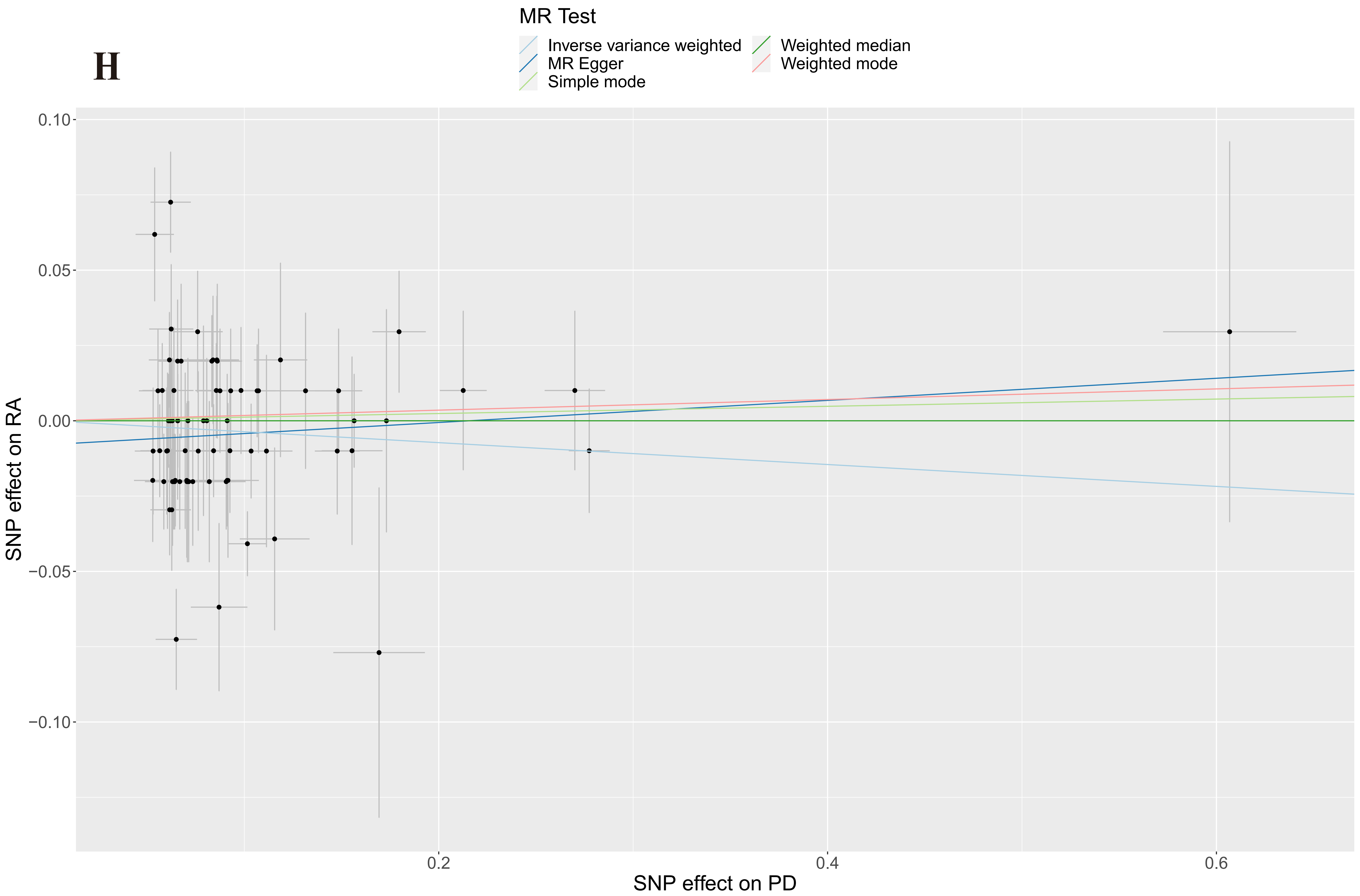

H
MR Test
Inverse variance weighted
MR Egger
Simple mode
Weighted median
Weighted mode
0.10
0.05
0.00
−0.05
−0.10
SNP effect on RA
0.2
0.4
0.6
SNP effect on PD

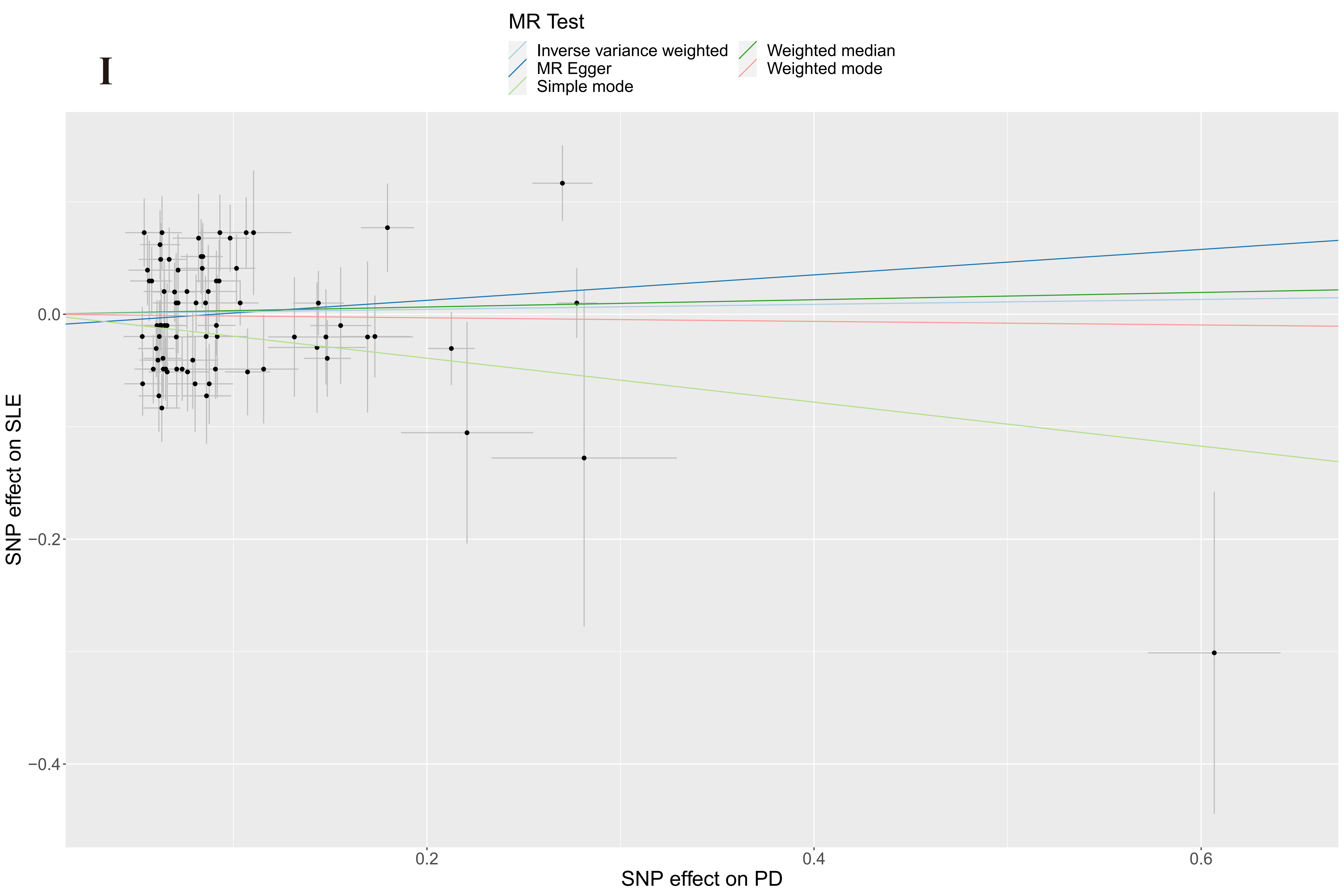

I
MR Test
Inverse variance weighted
MR Egger
Simple mode
Weighted median
Weighted mode
SNP effect on SLE
0.0
−0.2
−0.4
SNP effect on PD
0.2
0.4
0.6

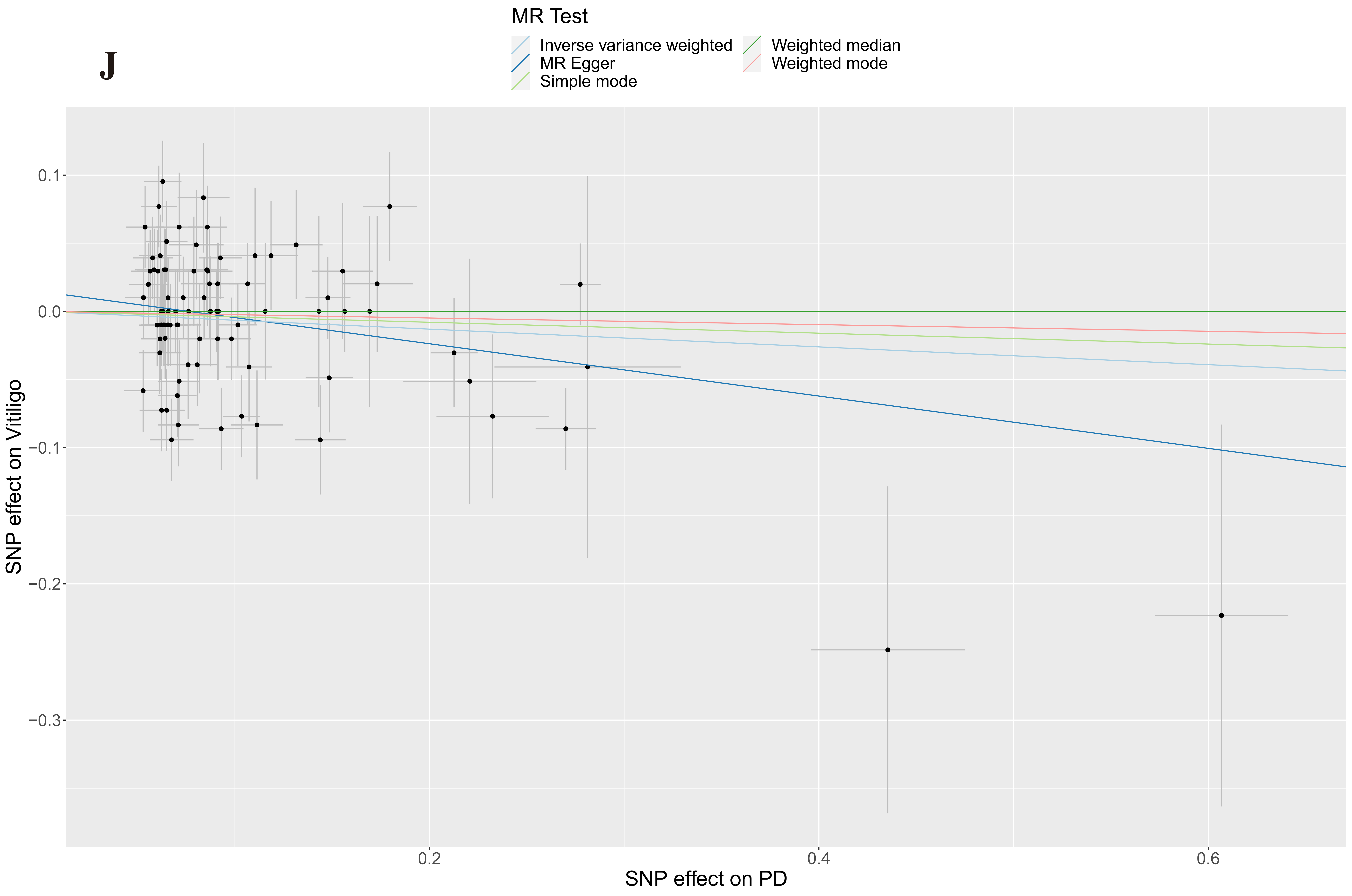

J
MR Test
Inverse variance weighted
MR Egger
Simple mode
Weighted median
Weighted mode
SNP effect on Vitiligo
SNP effect on PD
0.1
0.0
−0.1
−0.2
−0.3
0.2
0.4
0.6
