# Supplementary material for: Investigation of the causal association between Parkinson’s disease and autoimmune disorders: a bidirectional Mendelian randomization study
Source: Front Immunol. 2024 May 7;15:1370831. doi: 10.3389/fimmu.2024.1370831 (PMC11106379; doi:10.3389/fimmu.2024.1370831)
Supplement: Supplementary Figure 2 — Leave-one-out plots of MR tests assessing the effect of PD on AIDs. (A) PD on MS; (B) PD on NMOSD; (C) PD on MG; (D) PD on Asthma; (E) PD on IBD; (F) PD on CD; (G) PD on UC; (H) PD on IBS; (I) PD on T1D; (J) PD on RA; (K) PD on SLE; (L) PD on Vitiligo. PD, Parkinson’s disease; AIDs, autoimmune diseases; MS, multiple sclerosis; NMOSD, neuromyelitis optica spectrum disorder; MG, myasthenia gravis; IBD, inflammatory bowel disease; CD, Crohn’s disease; UC, ulcerative colitis; IBS, irritable bowel syndrome; T1D, type 1 diabetes; RA, rheumatoid arthritis; SLE, systemic lupus erythematosus. [file DataSheet_2.pdf]

Supplementary Figure 2. Leave-one-out plots of MR tests assessing the effect of PD on AIDs.

A

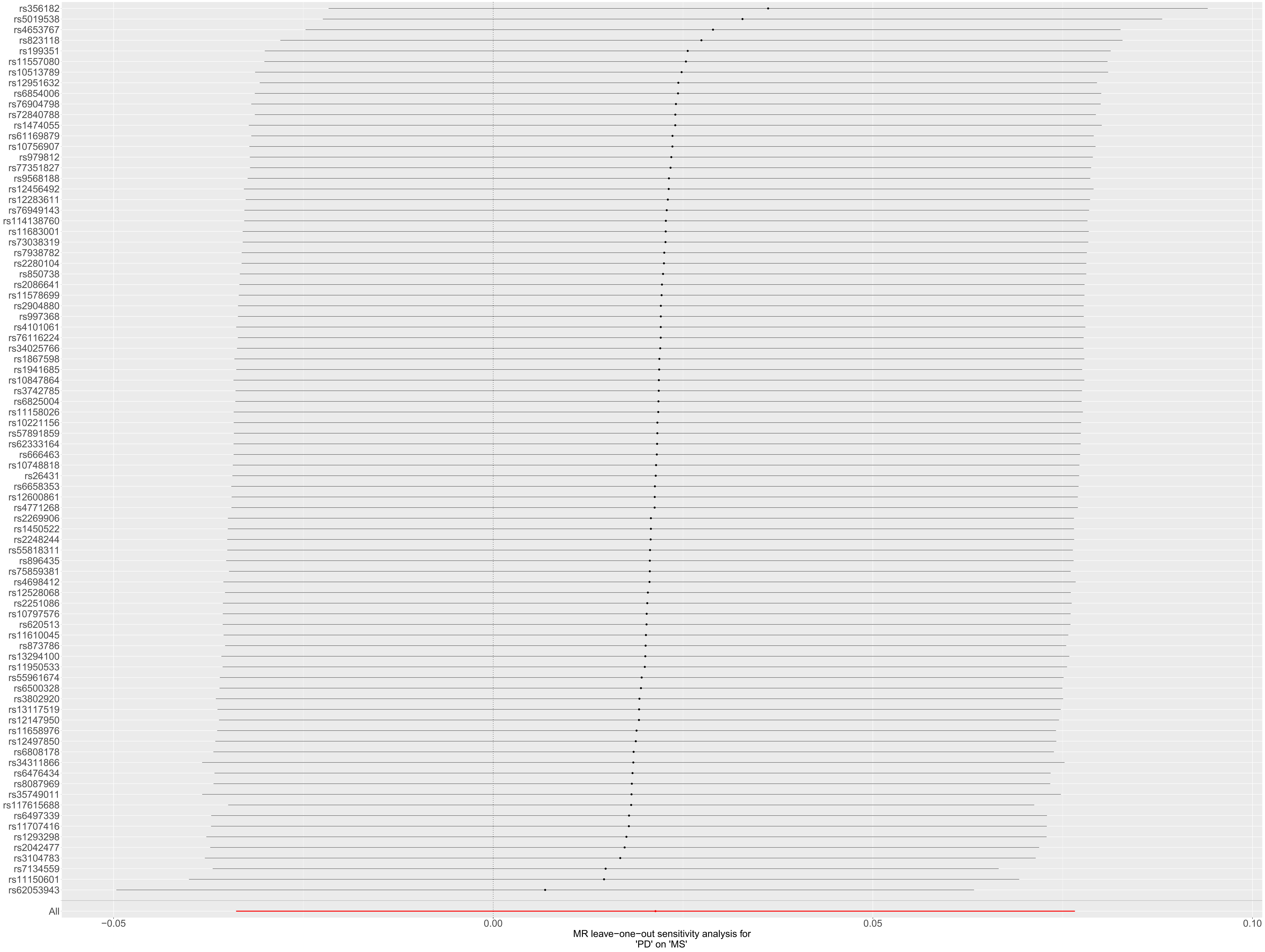

B

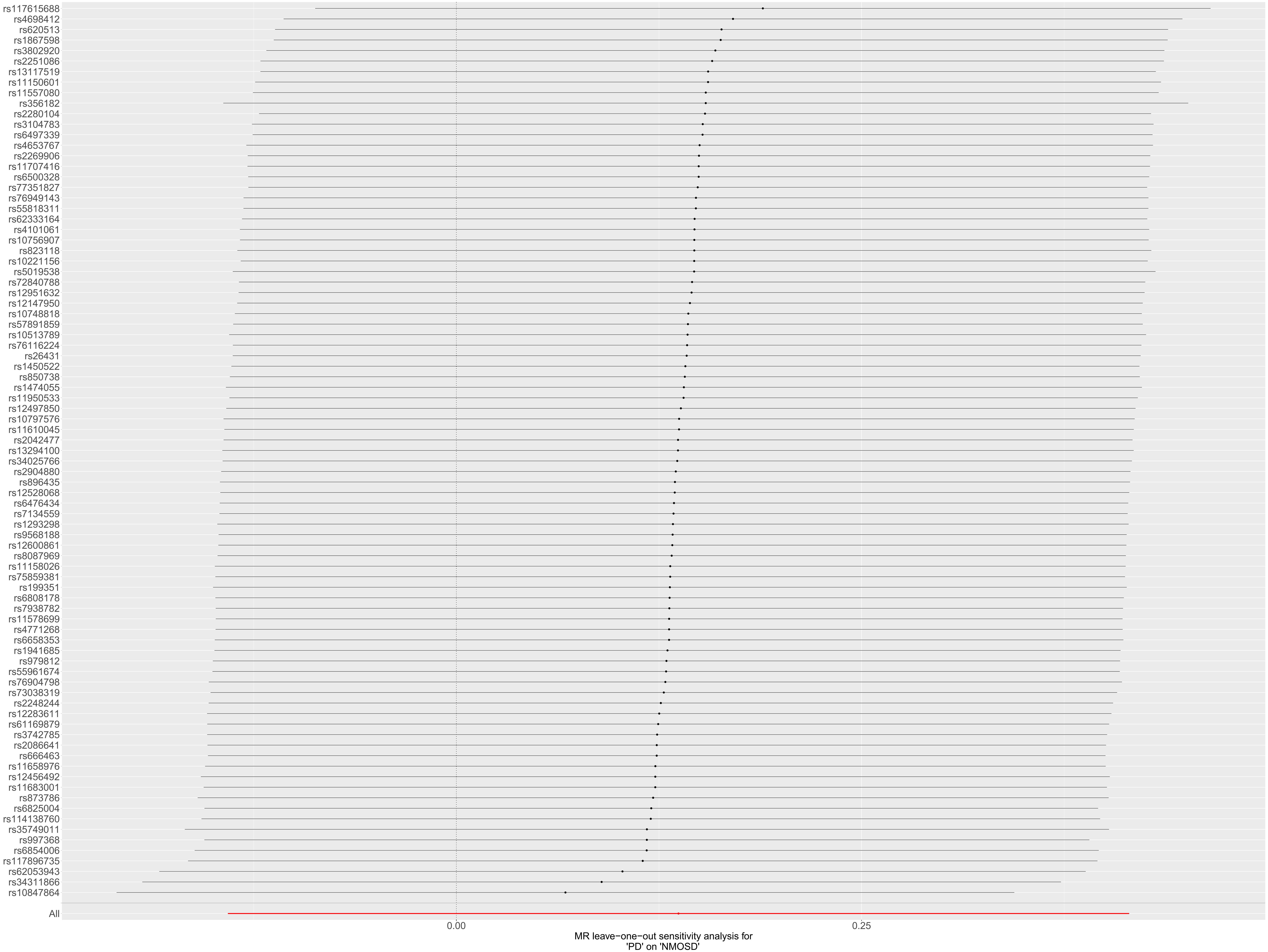
rs117615688
rs4698412
rs620513
rs1867598
rs3802920
rs2251086
rs13117519
rs11150601
rs11557080
rs356182
rs2280104
rs3104783
rs6497339
rs4653767
rs2269906
rs11707416
rs6500328
rs77351827
rs76949143
rs55818311
rs62333164
rs4101061
rs10756907
rs823118
rs10221156
rs5019538
rs72840788
rs12951632
rs12147950
rs10748818
rs57891859
rs10513789
rs76116224
rs26431
rs1450522
rs850738
rs1474055
rs11950533
rs12497850
rs10797576
rs11610045
rs2042477
rs13294100
rs34025766
rs2904880
rs896435
rs12528068
rs6476434
rs7134559
rs1293298
rs9568188
rs12600861
rs8087969
rs11158026
rs75859381
rs199351
rs6808178
rs7938782
rs11578699
rs4771268
rs6658353
rs1941685
rs979812
rs55961674
rs76904798
rs73038319
rs2248244
rs12283611
rs61169879
rs3742785
rs2086641
rs666463
rs11658976
rs12456492
rs11683001
rs873786
rs6825004
rs114138760
rs35749011
rs997368
rs6854006
rs117896735
rs62053943
rs34311866
rs10847864
All
0.00
0.25
MR leave−one−out sensitivity analysis for
'PD' on 'NMOSD'

C

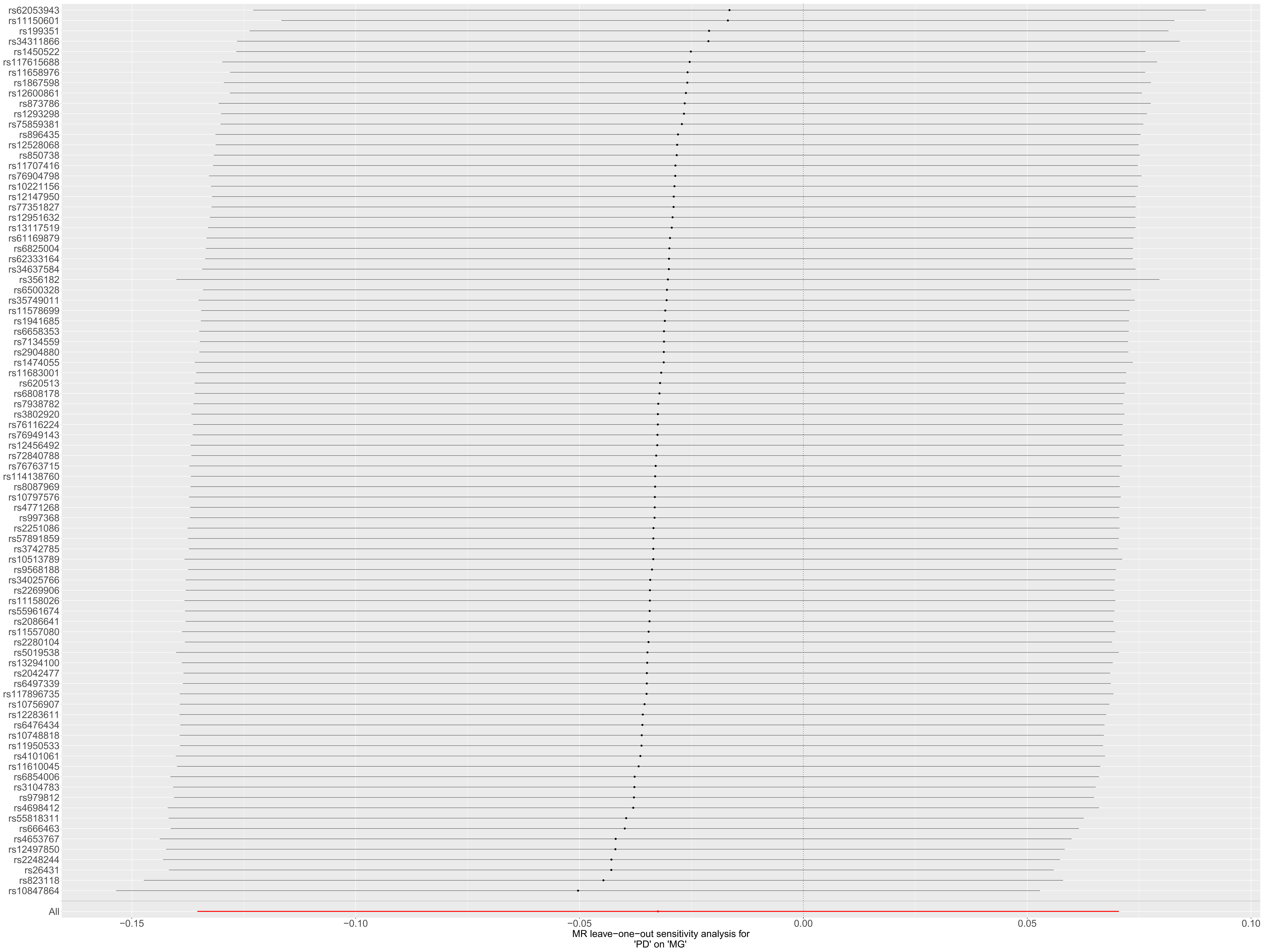

rs62053943
rs11150601
rs199351
rs34311866
rs1450522
rs117615688
rs11658976
rs1867598
rs12600861
rs873786
rs1293298
rs75859381
rs896435
rs12528068
rs850738
rs11707416
rs76904798
rs10221156
rs12147950
rs77351827
rs12951632
rs13117519
rs61169879
rs6825004
rs62333164
rs34637584
rs356182
rs6500328
rs35749011
rs11578699
rs1941685
rs6658353
rs7134559
rs2904880
rs1474055
rs11683001
rs620513
rs6808178
rs7938782
rs3802920
rs76116224
rs76949143
rs12456492
rs72840788
rs76763715
rs114138760
rs8087969
rs10797576
rs4771268
rs997368
rs2251086
rs57891859
rs3742785
rs10513789
rs9568188
rs34025766
rs2269906
rs11158026
rs55961674
rs2086641
rs11557080
rs2280104
rs5019538
rs13294100
rs2042477
rs6497339
rs117896735
rs10756907
rs12283611
rs6476434
rs10748818
rs11950533
rs4101061
rs11610045
rs6854006
rs3104783
rs979812
rs4698412
rs55818311
rs666463
rs4653767
rs12497850
rs2248244
rs26431
rs823118
rs10847864
All
−0.15
−0.10
−0.05
0.00
0.05
0.10
MR leave−one−out sensitivity analysis for
'PD' on 'MG'

D

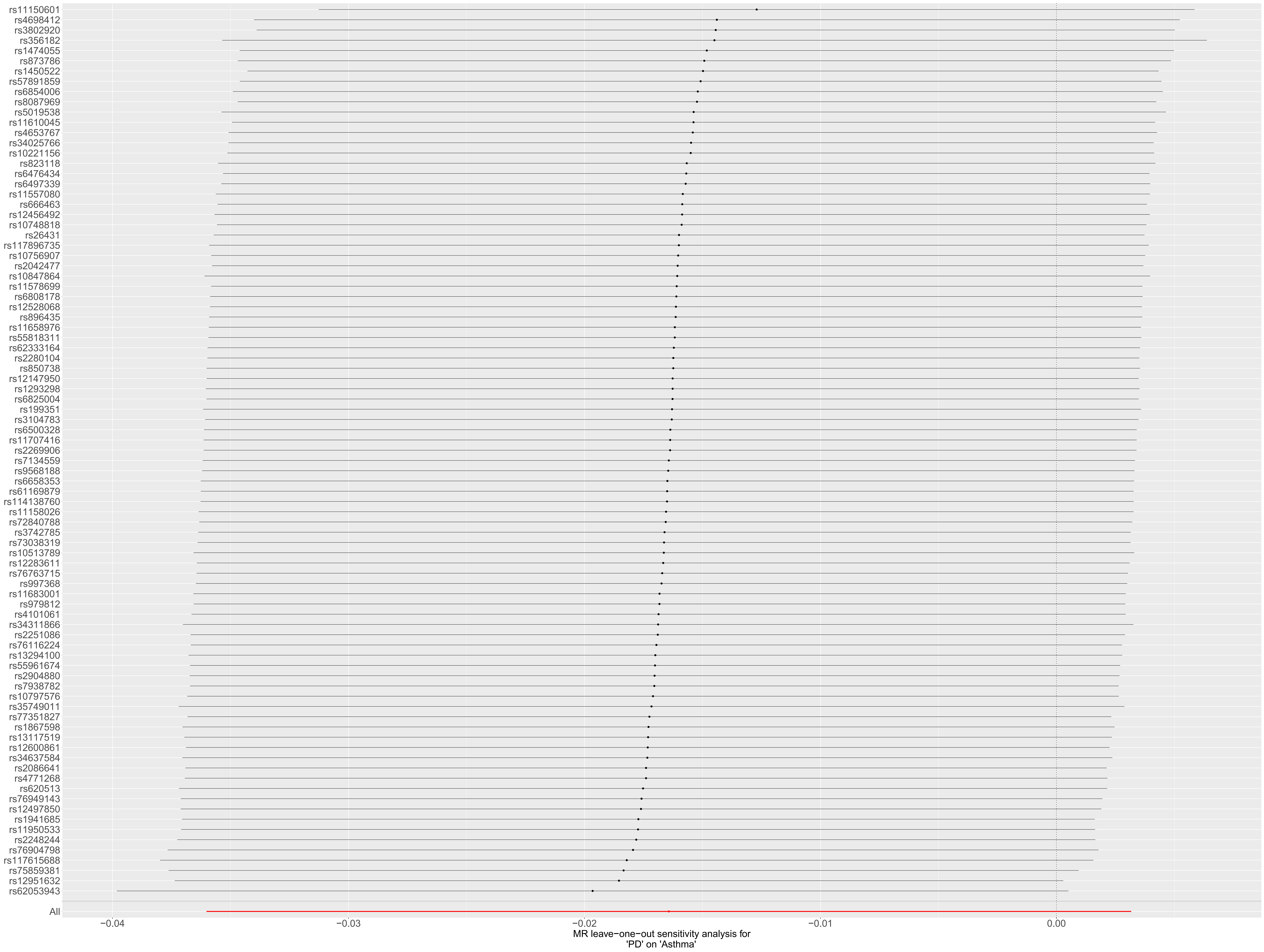
rs11150601
rs4698412
rs3802920
rs356182
rs1474055
rs873786
rs1450522
rs57891859
rs6854006
rs8087969
rs5019538
rs11610045
rs4653767
rs34025766
rs10221156
rs823118
rs6476434
rs6497339
rs11557080
rs666463
rs12456492
rs10748818
rs26431
rs117896735
rs10756907
rs2042477
rs10847864
rs11578699
rs6808178
rs12528068
rs896435
rs11658976
rs55818311
rs62333164
rs2280104
rs850738
rs12147950
rs1293298
rs6825004
rs199351
rs3104783
rs6500328
rs11707416
rs2269906
rs7134559
rs9568188
rs6658353
rs61169879
rs114138760
rs11158026
rs72840788
rs3742785
rs73038319
rs10513789
rs12283611
rs76763715
rs997368
rs11683001
rs979812
rs4101061
rs34311866
rs2251086
rs76116224
rs13294100
rs55961674
rs2904880
rs7938782
rs10797576
rs35749011
rs77351827
rs1867598
rs13117519
rs12600861
rs34637584
rs2086641
rs4771268
rs620513
rs76949143
rs12497850
rs1941685
rs11950533
rs2248244
rs76904798
rs117615688
rs75859381
rs12951632
rs62053943
All
−0.04
−0.03
−0.02
−0.01
0.00
MR leave−one−out sensitivity analysis for
'PD' on 'Asthma'

E

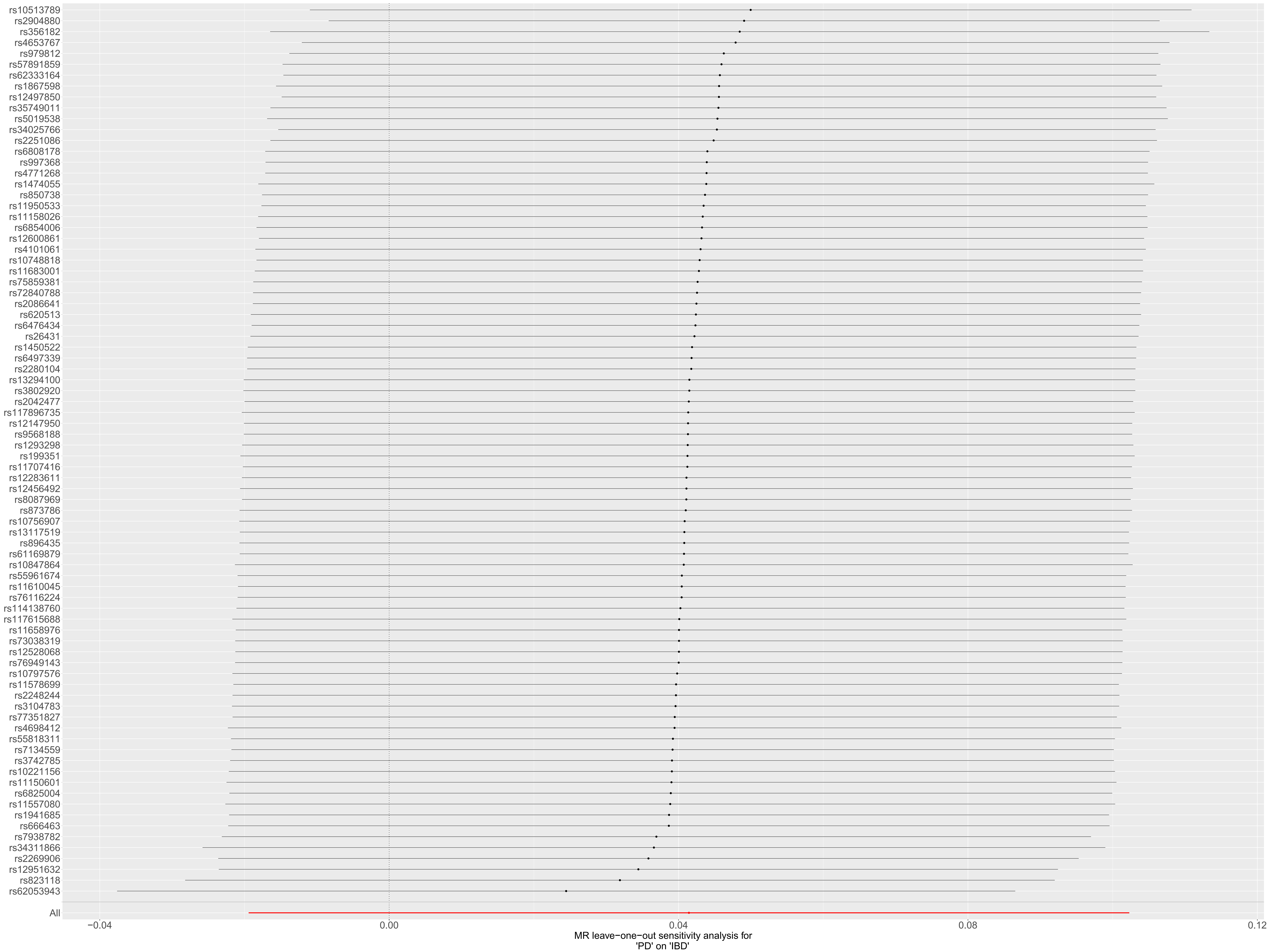
rs10513789
rs2904880
rs356182
rs4653767
rs979812
rs57891859
rs62333164
rs1867598
rs12497850
rs35749011
rs5019538
rs34025766
rs2251086
rs6808178
rs997368
rs4771268
rs1474055
rs850738
rs11950533
rs11158026
rs6854006
rs12600861
rs4101061
rs10748818
rs11683001
rs75859381
rs72840788
rs2086641
rs620513
rs6476434
rs26431
rs1450522
rs6497339
rs2280104
rs13294100
rs3802920
rs2042477
rs117896735
rs12147950
rs9568188
rs1293298
rs199351
rs11707416
rs12283611
rs12456492
rs8087969
rs873786
rs10756907
rs13117519
rs896435
rs61169879
rs10847864
rs55961674
rs11610045
rs76116224
rs114138760
rs117615688
rs11658976
rs73038319
rs12528068
rs76949143
rs10797576
rs11578699
rs2248244
rs3104783
rs77351827
rs4698412
rs55818311
rs7134559
rs3742785
rs10221156
rs11150601
rs6825004
rs11557080
rs1941685
rs666463
rs7938782
rs34311866
rs2269906
rs12951632
rs823118
rs62053943
All
−0.04
0.00
0.04
0.08
0.12
MR leave−one−out sensitivity analysis for
'PD' on 'IBD'

F

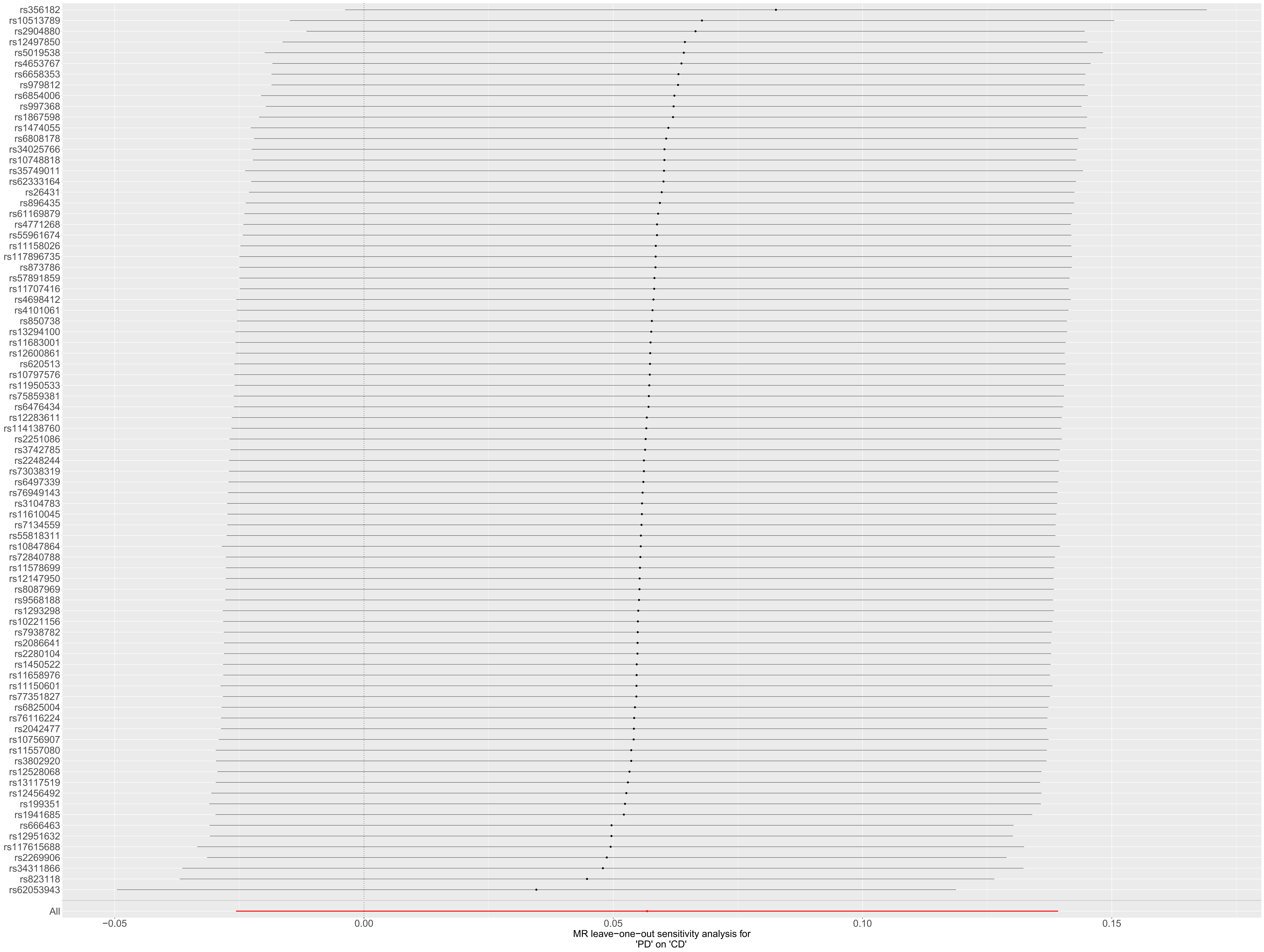
rs356182
rs10513789
rs2904880
rs12497850
rs5019538
rs4653767
rs6658353
rs979812
rs6854006
rs997368
rs1867598
rs1474055
rs6808178
rs34025766
rs10748818
rs35749011
rs62333164
rs26431
rs896435
rs61169879
rs4771268
rs55961674
rs11158026
rs117896735
rs873786
rs57891859
rs11707416
rs4698412
rs4101061
rs850738
rs13294100
rs11683001
rs12600861
rs620513
rs10797576
rs11950533
rs75859381
rs6476434
rs12283611
rs114138760
rs2251086
rs3742785
rs2248244
rs73038319
rs6497339
rs76949143
rs3104783
rs11610045
rs7134559
rs55818311
rs10847864
rs72840788
rs11578699
rs12147950
rs8087969
rs9568188
rs1293298
rs10221156
rs7938782
rs2086641
rs2280104
rs1450522
rs11658976
rs11150601
rs77351827
rs6825004
rs76116224
rs2042477
rs10756907
rs11557080
rs3802920
rs12528068
rs13117519
rs12456492
rs199351
rs1941685
rs666463
rs12951632
rs117615688
rs2269906
rs34311866
rs823118
rs62053943
All
−0.05
0.00
0.05
0.10
0.15
MR leave−one−out sensitivity analysis for
'PD' on 'CD'

G

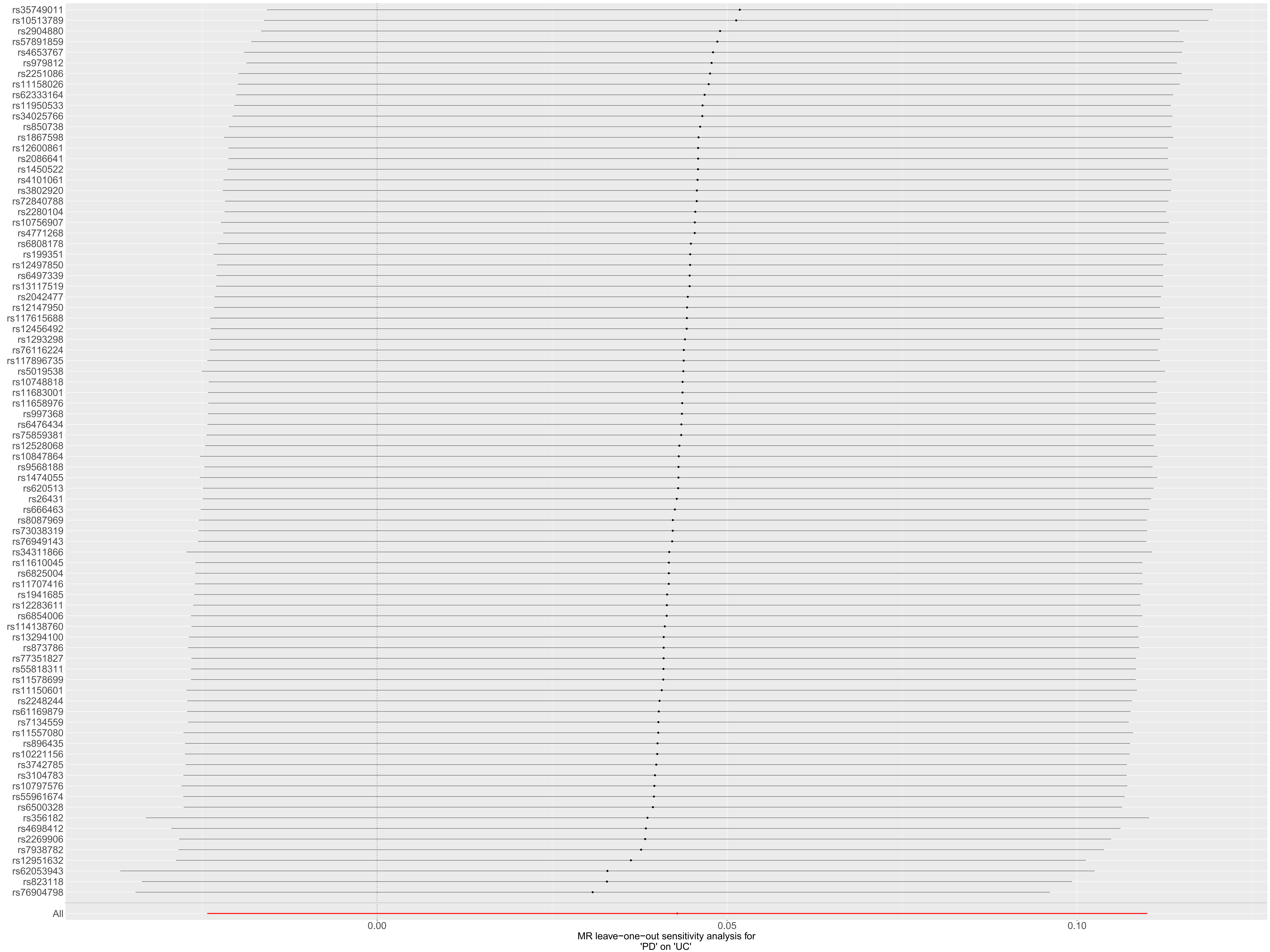
rs35749011
rs10513789
rs2904880
rs57891859
rs4653767
rs979812
rs2251086
rs11158026
rs62333164
rs11950533
rs34025766
rs850738
rs1867598
rs12600861
rs2086641
rs1450522
rs4101061
rs3802920
rs72840788
rs2280104
rs10756907
rs4771268
rs6808178
rs199351
rs12497850
rs6497339
rs13117519
rs2042477
rs12147950
rs117615688
rs12456492
rs1293298
rs76116224
rs117896735
rs5019538
rs10748818
rs11683001
rs11658976
rs997368
rs6476434
rs75859381
rs12528068
rs10847864
rs9568188
rs1474055
rs620513
rs26431
rs666463
rs8087969
rs73038319
rs76949143
rs34311866
rs11610045
rs6825004
rs11707416
rs1941685
rs12283611
rs6854006
rs114138760
rs13294100
rs873786
rs77351827
rs55818311
rs11578699
rs11150601
rs2248244
rs61169879
rs7134559
rs11557080
rs896435
rs10221156
rs3742785
rs3104783
rs10797576
rs55961674
rs6500328
rs356182
rs4698412
rs2269906
rs7938782
rs12951632
rs62053943
rs823118
rs76904798
All
0.00
0.05
0.10
MR leave−one−out sensitivity analysis for
'PD' on 'UC'

H

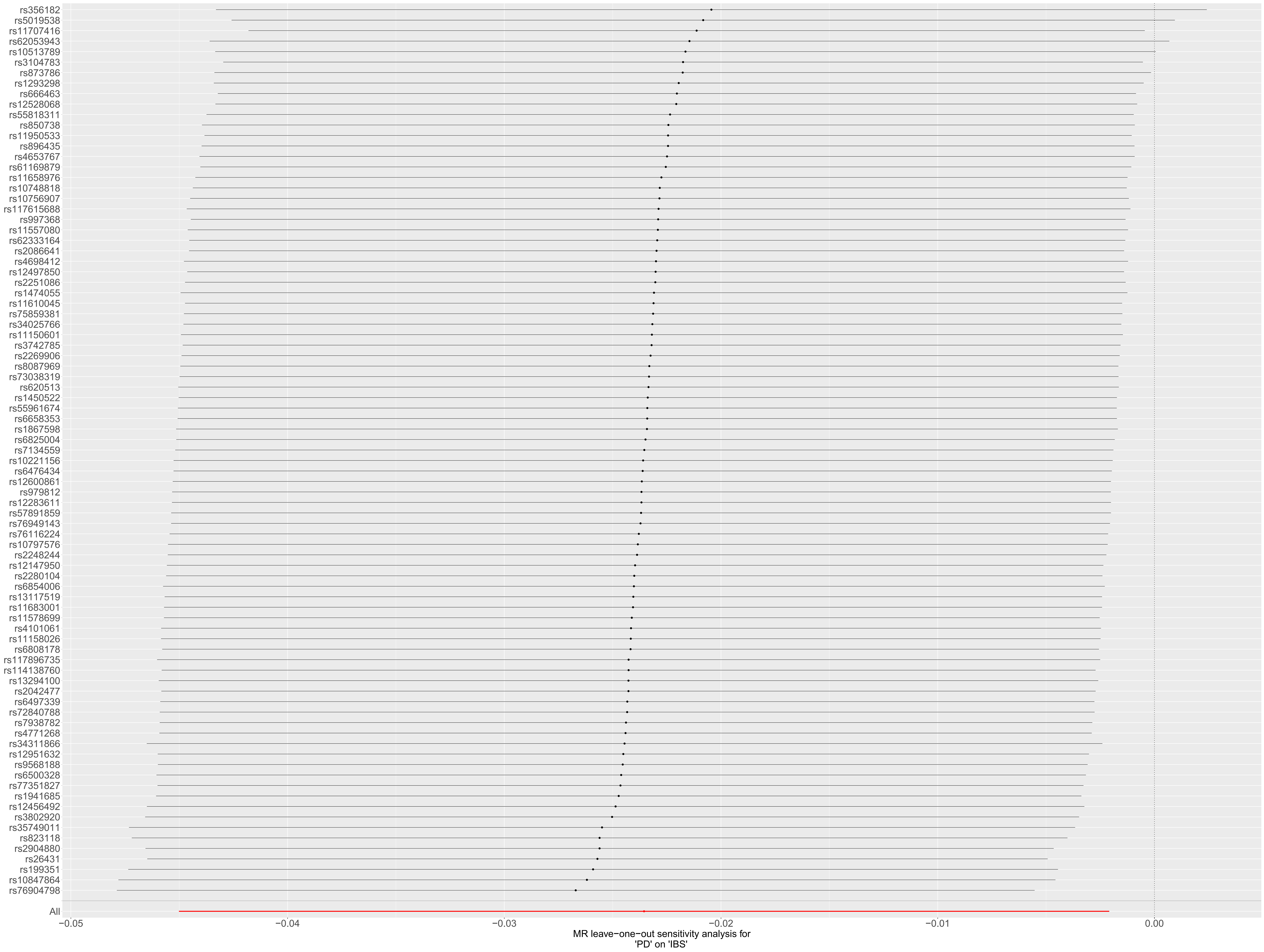

rs356182
rs5019538
rs11707416
rs62053943
rs10513789
rs3104783
rs873786
rs1293298
rs666463
rs12528068
rs55818311
rs850738
rs11950533
rs896435
rs4653767
rs61169879
rs11658976
rs10748818
rs10756907
rs117615688
rs997368
rs11557080
rs62333164
rs2086641
rs4698412
rs12497850
rs2251086
rs1474055
rs11610045
rs75859381
rs34025766
rs11150601
rs3742785
rs2269906
rs8087969
rs73038319
rs620513
rs1450522
rs55961674
rs6658353
rs1867598
rs6825004
rs7134559
rs10221156
rs6476434
rs12600861
rs979812
rs12283611
rs57891859
rs76949143
rs76116224
rs10797576
rs2248244
rs12147950
rs2280104
rs6854006
rs13117519
rs11683001
rs11578699
rs4101061
rs11158026
rs6808178
rs117896735
rs114138760
rs13294100
rs2042477
rs6497339
rs72840788
rs7938782
rs4771268
rs34311866
rs12951632
rs9568188
rs6500328
rs77351827
rs1941685
rs12456492
rs3802920
rs35749011
rs823118
rs2904880
rs26431
rs199351
rs10847864
rs76904798
All
−0.05
−0.04
−0.03
−0.02
−0.01
0.00
MR leave−one−out sensitivity analysis for
'PD' on 'IBS'

I

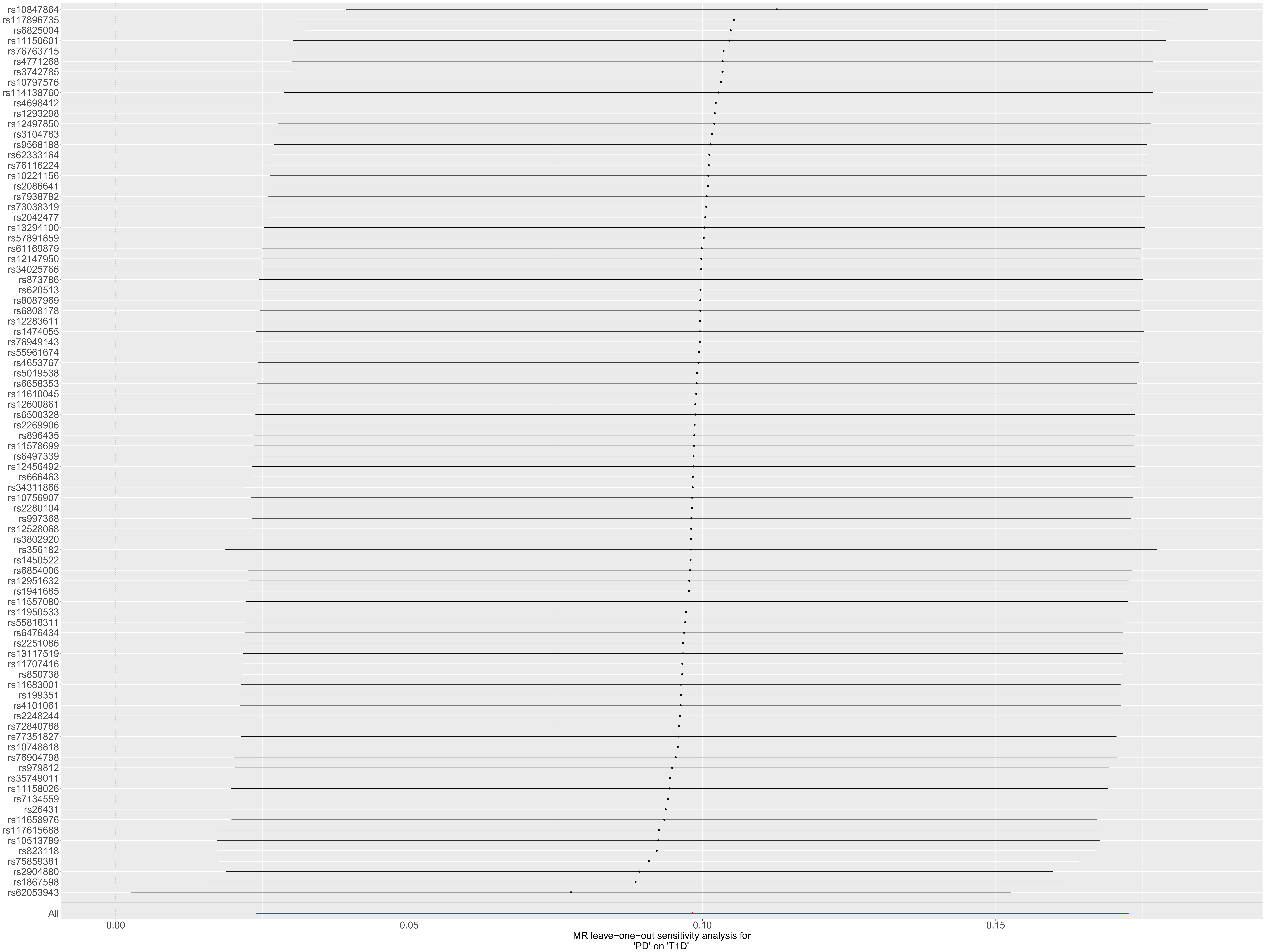
rs10847864
rs117896735
rs6825004
rs11150601
rs76763715
rs4771268
rs3742785
rs10797576
rs114138760
rs4698412
rs1293298
rs12497850
rs3104783
rs9568188
rs62333164
rs76116224
rs10221156
rs2086641
rs7938782
rs73038319
rs2042477
rs13294100
rs57891859
rs61169879
rs12147950
rs34025766
rs873786
rs620513
rs8087969
rs6808178
rs12283611
rs1474055
rs76949143
rs55961674
rs4653767
rs5019538
rs6658353
rs11610045
rs12600861
rs6500328
rs2269906
rs896435
rs11578699
rs6497339
rs12456492
rs666463
rs34311866
rs10756907
rs2280104
rs997368
rs12528068
rs3802920
rs356182
rs1450522
rs6854006
rs12951632
rs1941685
rs11557080
rs11950533
rs55818311
rs6476434
rs2251086
rs13117519
rs11707416
rs850738
rs11683001
rs199351
rs4101061
rs2248244
rs72840788
rs77351827
rs10748818
rs76904798
rs979812
rs35749011
rs11158026
rs7134559
rs26431
rs11658976
rs117615688
rs10513789
rs823118
rs75859381
rs2904880
rs1867598
rs62053943
All
0.00
0.05
0.10
0.15
MR leave−one−out sensitivity analysis for
'PD' on 'T1D'

J

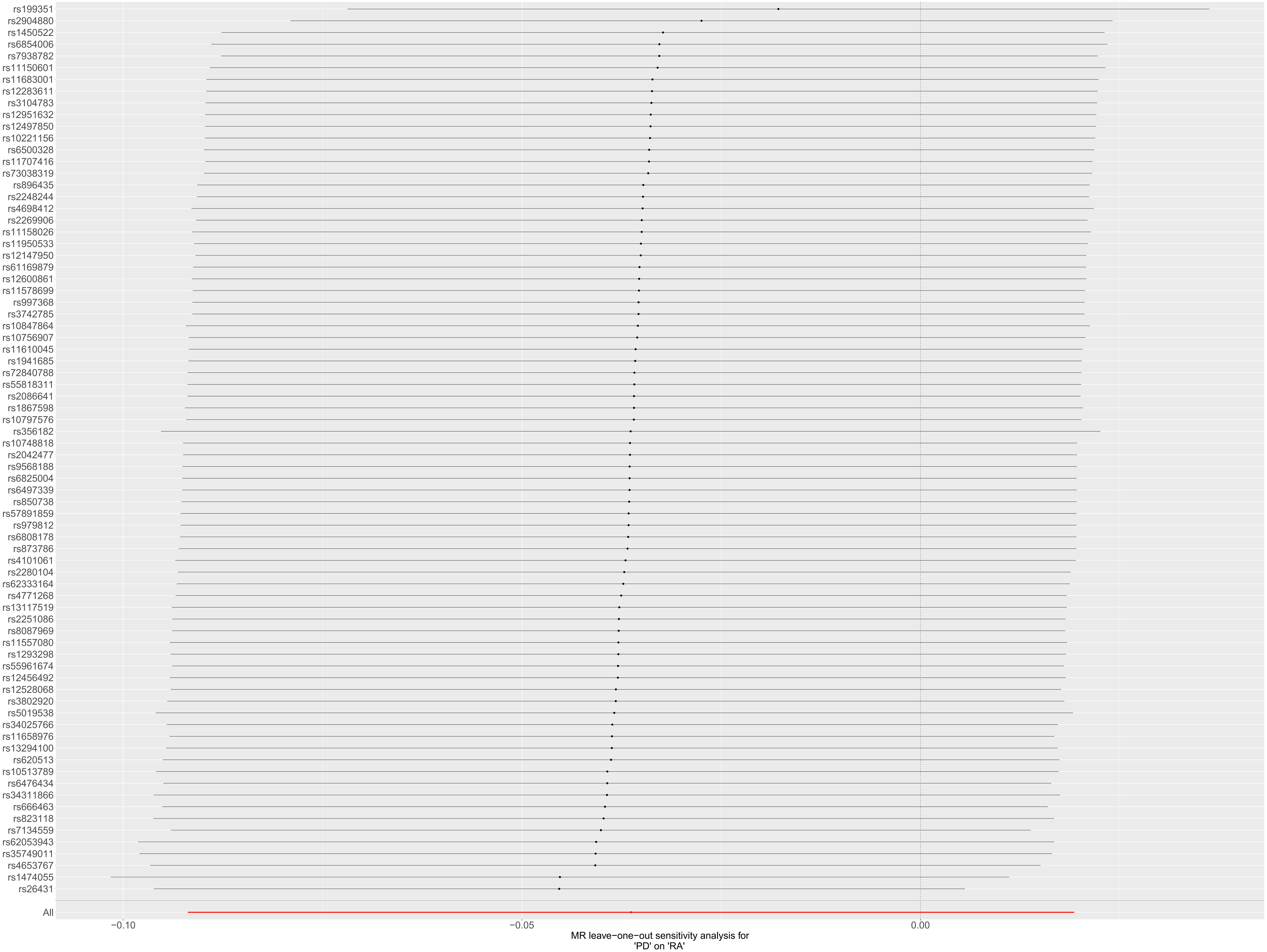
rs199351
rs2904880
rs1450522
rs6854006
rs7938782
rs11150601
rs11683001
rs12283611
rs3104783
rs12951632
rs12497850
rs10221156
rs6500328
rs11707416
rs73038319
rs896435
rs2248244
rs4698412
rs2269906
rs11158026
rs11950533
rs12147950
rs61169879
rs12600861
rs11578699
rs997368
rs3742785
rs10847864
rs10756907
rs11610045
rs1941685
rs72840788
rs55818311
rs2086641
rs1867598
rs10797576
rs356182
rs10748818
rs2042477
rs9568188
rs6825004
rs6497339
rs850738
rs57891859
rs979812
rs6808178
rs873786
rs4101061
rs2280104
rs62333164
rs4771268
rs13117519
rs2251086
rs8087969
rs11557080
rs1293298
rs55961674
rs12456492
rs12528068
rs3802920
rs5019538
rs34025766
rs11658976
rs13294100
rs620513
rs10513789
rs6476434
rs34311866
rs666463
rs823118
rs7134559
rs62053943
rs35749011
rs4653767
rs1474055
rs26431
All
−0.10
−0.05
0.00
MR leave−one−out sensitivity analysis for
'PD' on 'RA'

K

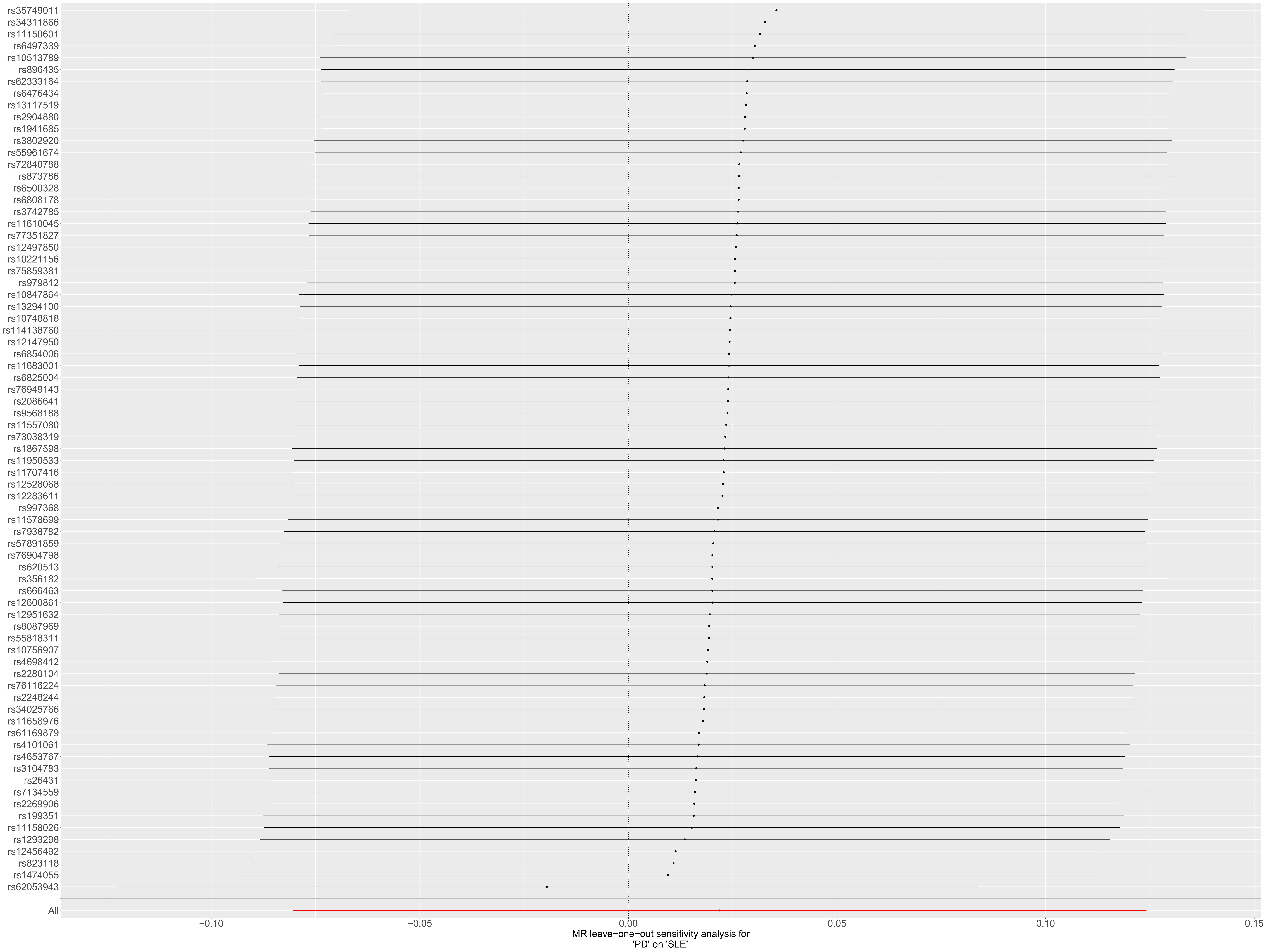
rs35749011
rs34311866
rs11150601
rs6497339
rs10513789
rs896435
rs62333164
rs6476434
rs13117519
rs2904880
rs1941685
rs3802920
rs55961674
rs72840788
rs873786
rs6500328
rs6808178
rs3742785
rs11610045
rs77351827
rs12497850
rs10221156
rs75859381
rs979812
rs10847864
rs13294100
rs10748818
rs114138760
rs12147950
rs6854006
rs11683001
rs6825004
rs76949143
rs2086641
rs9568188
rs11557080
rs73038319
rs1867598
rs11950533
rs11707416
rs12528068
rs12283611
rs997368
rs11578699
rs7938782
rs57891859
rs76904798
rs620513
rs356182
rs666463
rs12600861
rs12951632
rs8087969
rs55818311
rs10756907
rs4698412
rs2280104
rs76116224
rs2248244
rs34025766
rs11658976
rs61169879
rs4101061
rs4653767
rs3104783
rs26431
rs7134559
rs2269906
rs199351
rs11158026
rs1293298
rs12456492
rs823118
rs1474055
rs62053943
All
−0.10
−0.05
0.00
0.05
0.10
0.15
MR leave−one−out sensitivity analysis for
'PD' on 'SLE'

L

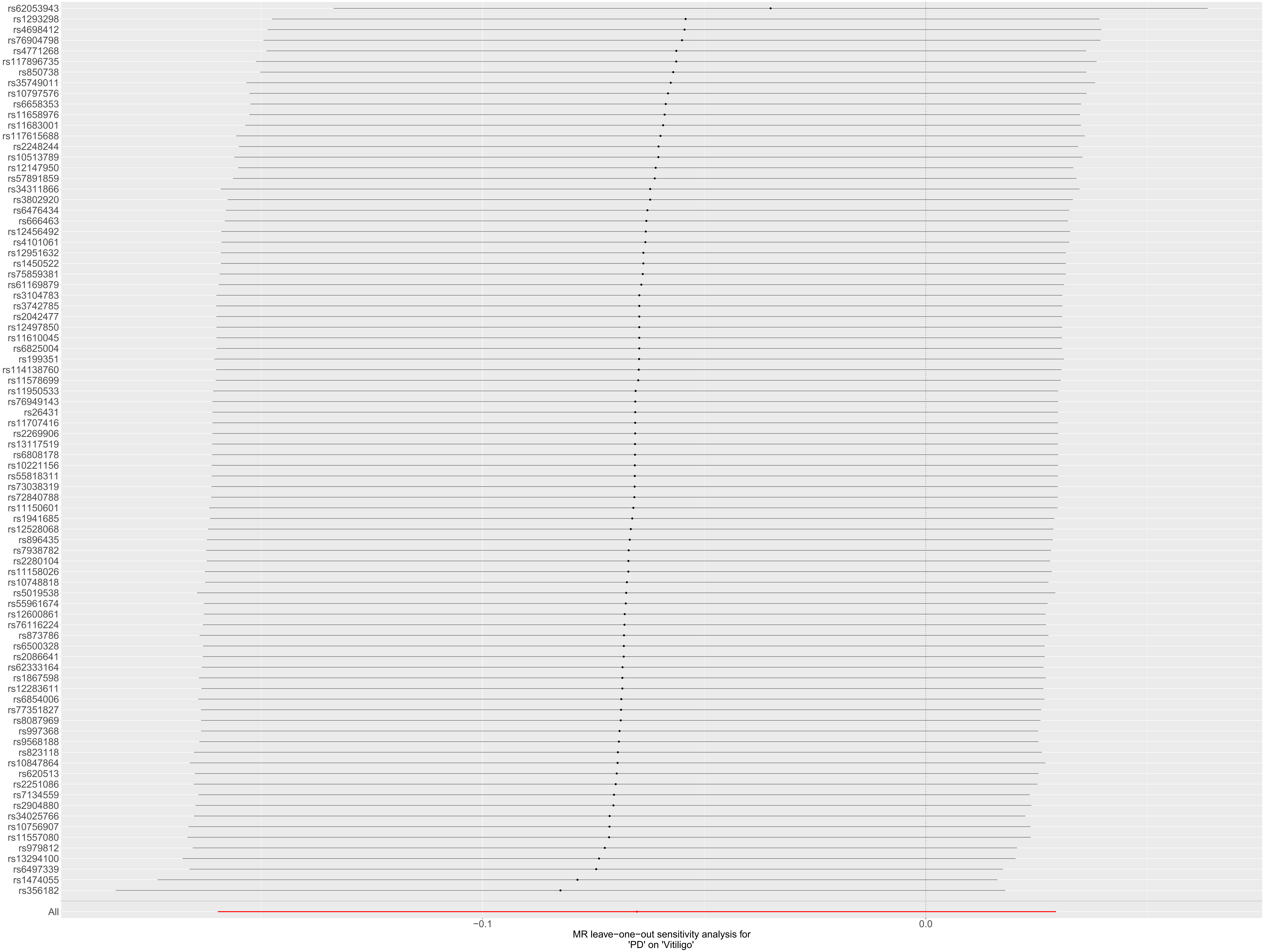

rs62053943
rs1293298
rs4698412
rs76904798
rs4771268
rs117896735
rs850738
rs35749011
rs10797576
rs6658353
rs11658976
rs11683001
rs117615688
rs2248244
rs10513789
rs12147950
rs57891859
rs34311866
rs3802920
rs6476434
rs666463
rs12456492
rs4101061
rs12951632
rs1450522
rs75859381
rs61169879
rs3104783
rs3742785
rs2042477
rs12497850
rs11610045
rs6825004
rs199351
rs114138760
rs11578699
rs11950533
rs76949143
rs26431
rs11707416
rs2269906
rs13117519
rs6808178
rs10221156
rs55818311
rs73038319
rs72840788
rs11150601
rs1941685
rs12528068
rs896435
rs7938782
rs2280104
rs11158026
rs10748818
rs5019538
rs55961674
rs12600861
rs76116224
rs873786
rs6500328
rs2086641
rs62333164
rs1867598
rs12283611
rs6854006
rs77351827
rs8087969
rs997368
rs9568188
rs823118
rs10847864
rs620513
rs2251086
rs7134559
rs2904880
rs34025766
rs10756907
rs11557080
rs979812
rs13294100
rs6497339
rs1474055
rs356182
All
−0.1
0.0
MR leave−one−out sensitivity analysis for
'PD' on 'Vitiligo'
