# Supplementary material for: Investigation of the causal association between Parkinson’s disease and autoimmune disorders: a bidirectional Mendelian randomization study
Source: Front Immunol. 2024 May 7;15:1370831. doi: 10.3389/fimmu.2024.1370831 (PMC11106379; doi:10.3389/fimmu.2024.1370831)
Supplement: Supplementary Figure 3 — Scatter plots of MR tests assessing the effect of AIDs on PD. (A) MS on PD; (B) MG on PD; (C) Asthma on PD; (D) IBD on PD; (E) CD on PD; (F) UC on PD; (G) IBS on PD; (H) CeD on PD; (I) PBC on PD; (J) PSC on PD; (K) T1D on PD; (L) AS on PD; (M) RA on PD; (N) SLE on PD; (O) Psoriasis on PD; (P) Vitiligo on PD. AIDs, autoimmune diseases; PD, Parkinson’s disease; MS, multiple sclerosis; MG, myasthenia gravis; IBD, inflammatory bowel disease; CD, Crohn’s disease; UC, ulcerative colitis; IBS, irritable bowel syndrome; CeD, celiac disease; PBC, primary biliary cirrhosis; PSC, primary sclerosing cholangitis; T1D, type 1 diabetes; AS, ankylosing spondylitis; RA, rheumatoid arthritis; SLE, systemic lupus erythematosus. [file DataSheet_3.pdf]

Supplementary Figure 3. Scatter plots of MR tests assessing the effect of AIDs on PD.

A

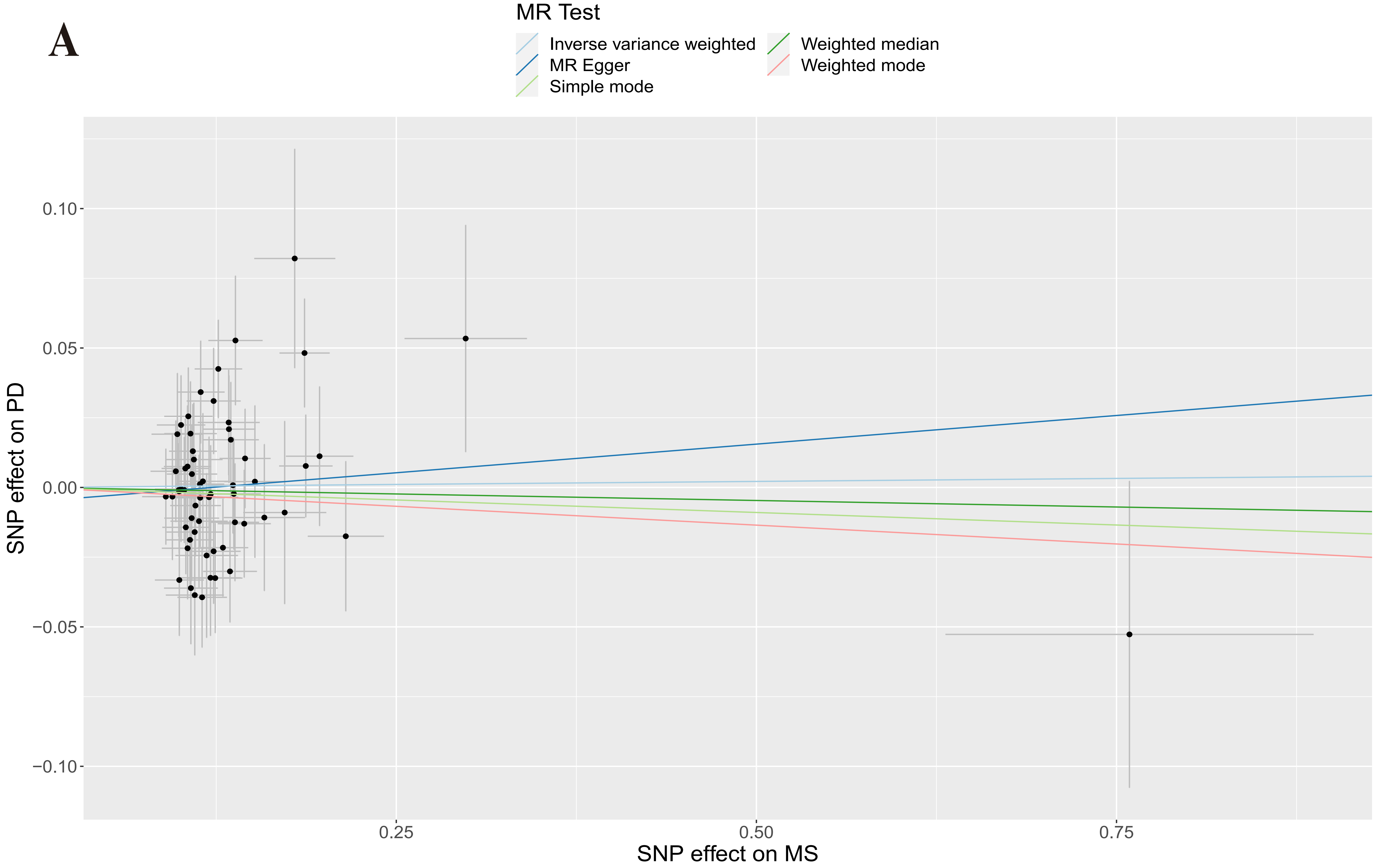

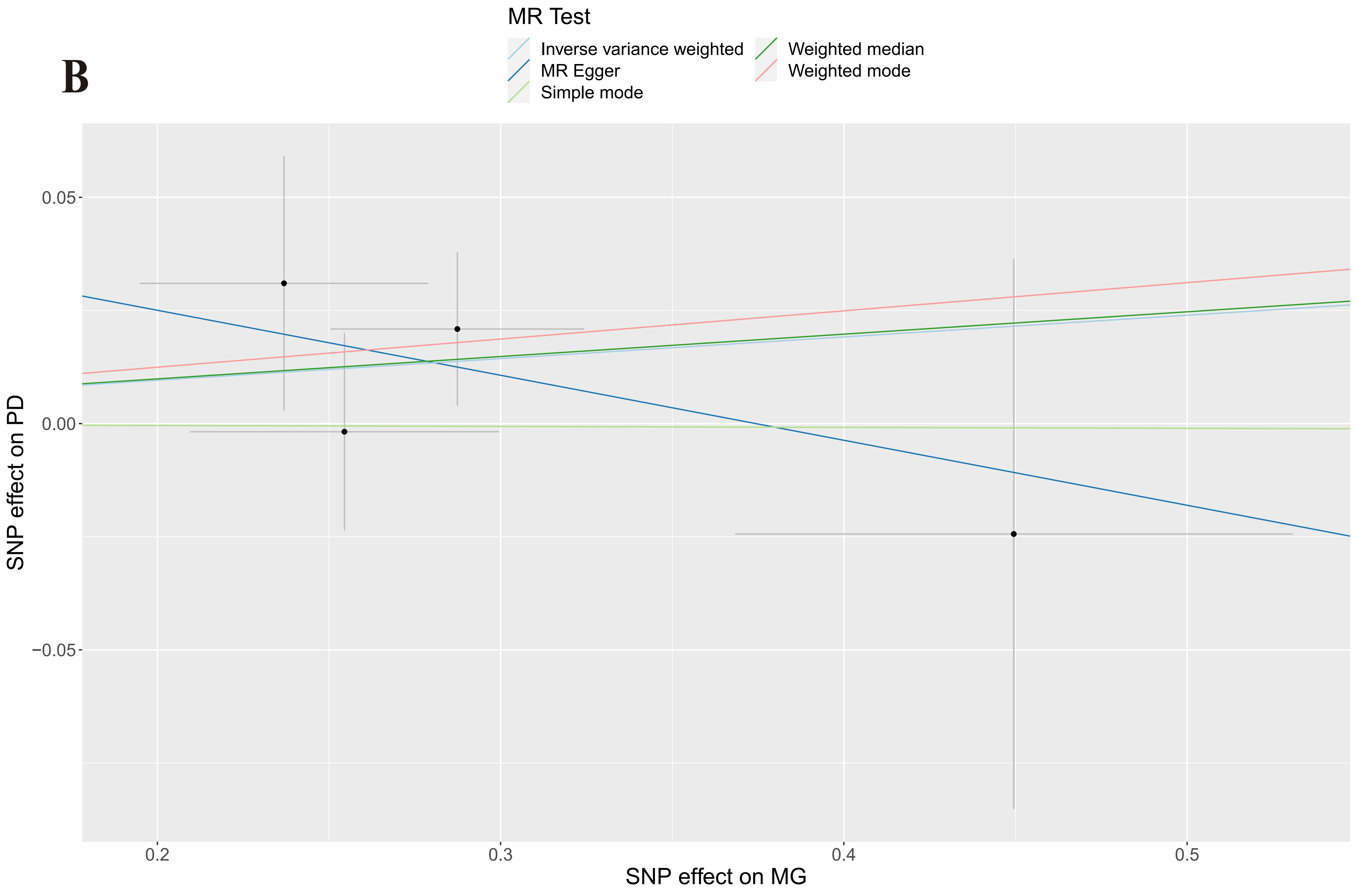
B
MR Test
Inverse variance weighted
MR Egger
Simple mode
Weighted median
Weighted mode
0.05
0.00
−0.05
0.2
0.3
0.4
0.5
SNP effect on PD
SNP effect on MG

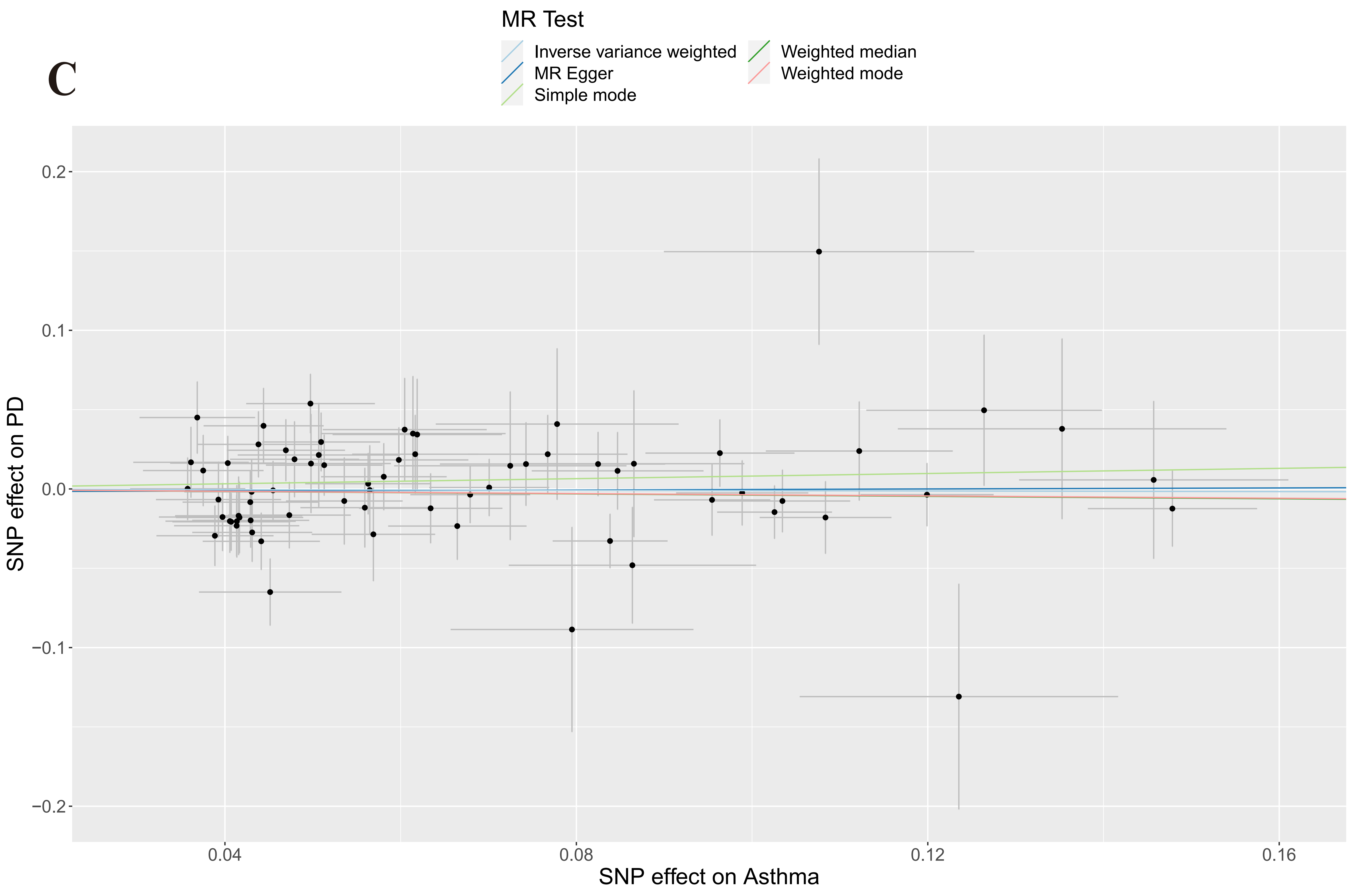
C
MR Test
Inverse variance weighted
MR Egger
Simple mode
Weighted median
Weighted mode
0.2
0.1
0.0
−0.1
−0.2
SNP effect on PD
0.04
0.08
0.12
0.16
SNP effect on Asthma

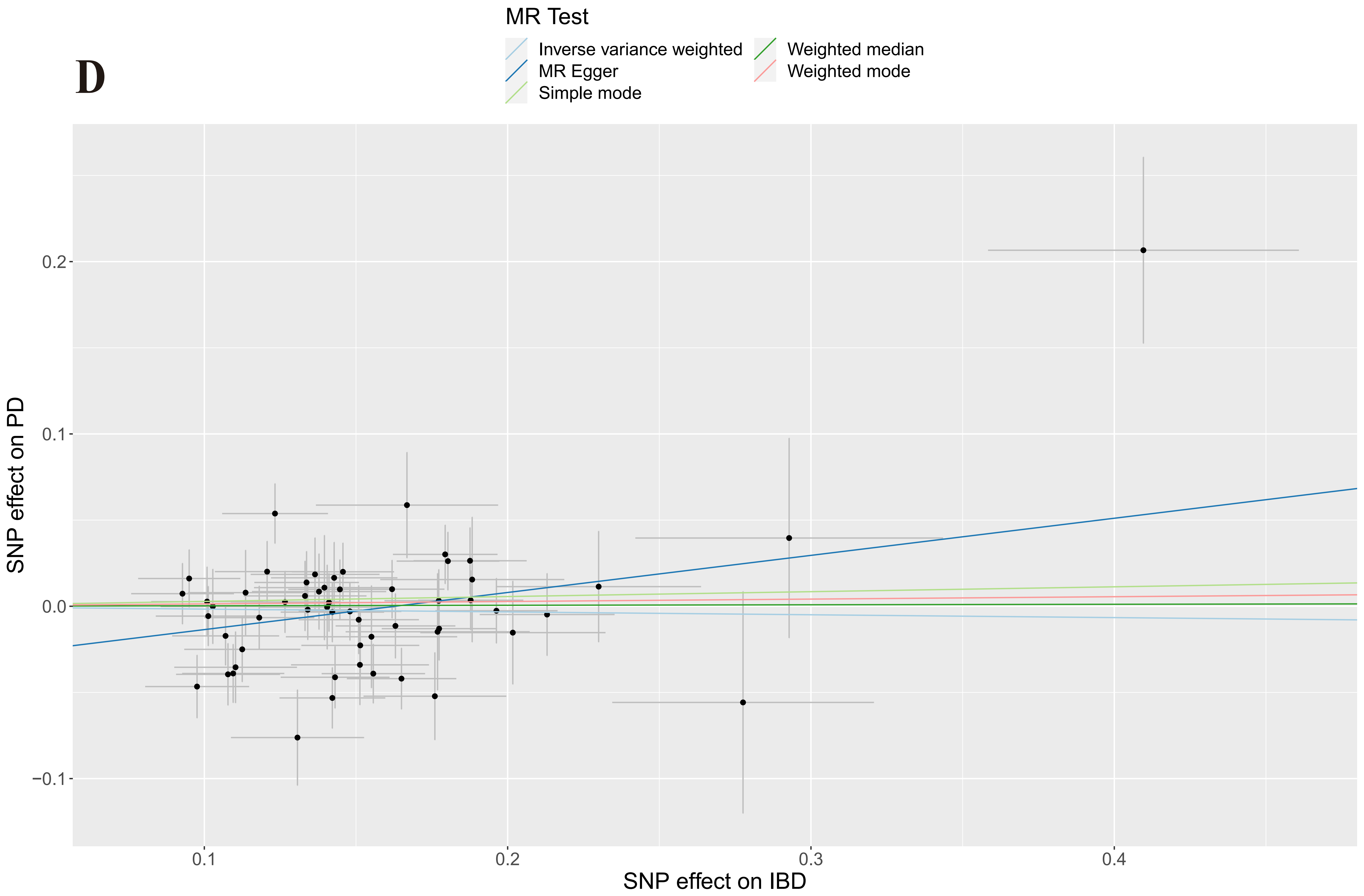

D
MR Test
Inverse variance weighted
MR Egger
Simple mode
Weighted median
Weighted mode
SNP effect on PD
0.2
0.1
0.0
−0.1
0.1
0.2
0.3
0.4
SNP effect on IBD

E

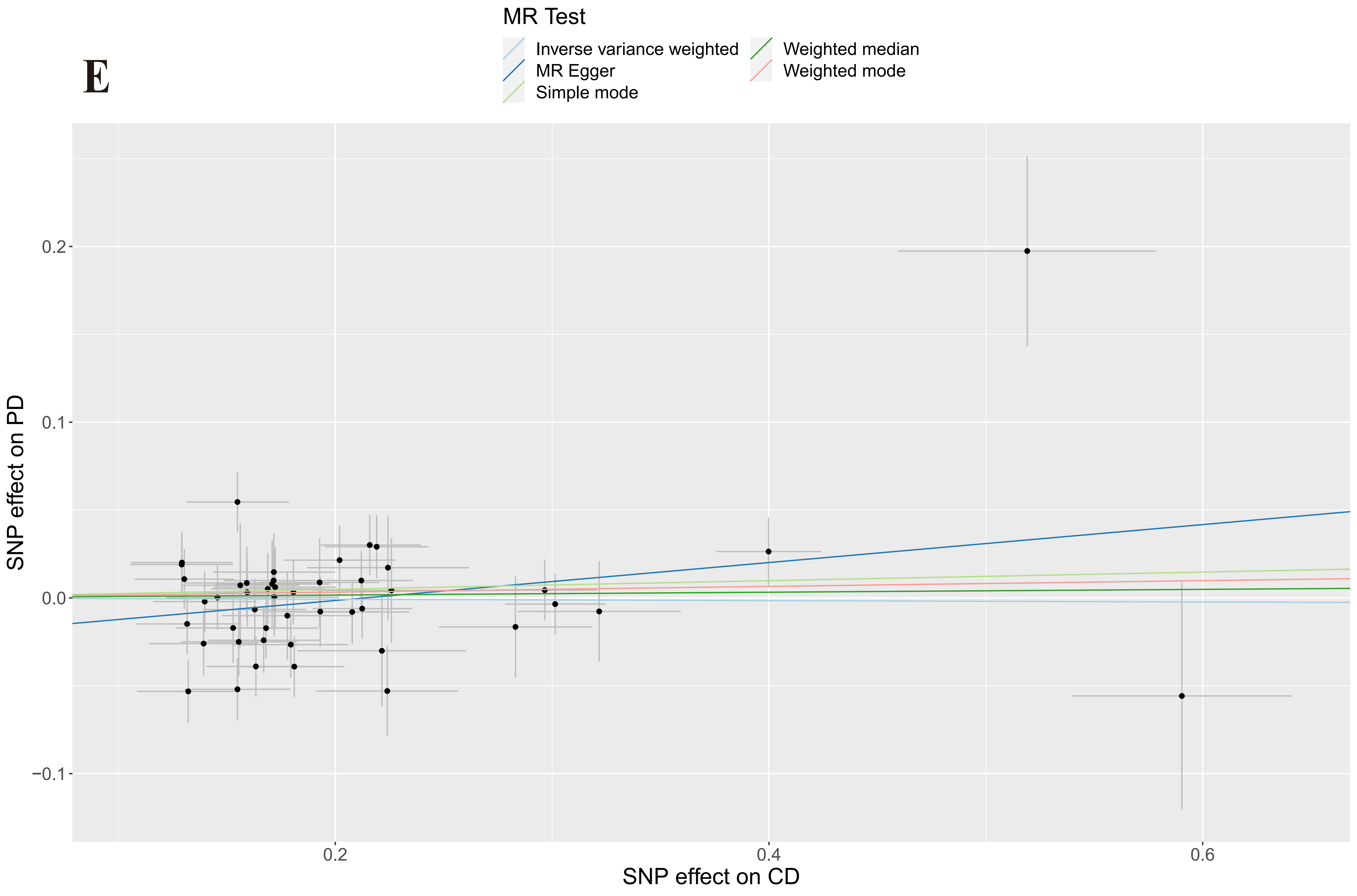
MR Test
Inverse variance weighted
MR Egger
Simple mode
Weighted median
Weighted mode
SNP effect on PD
0.2
0.1
0.0
−0.1
SNP effect on CD
0.2
0.4
0.6

F

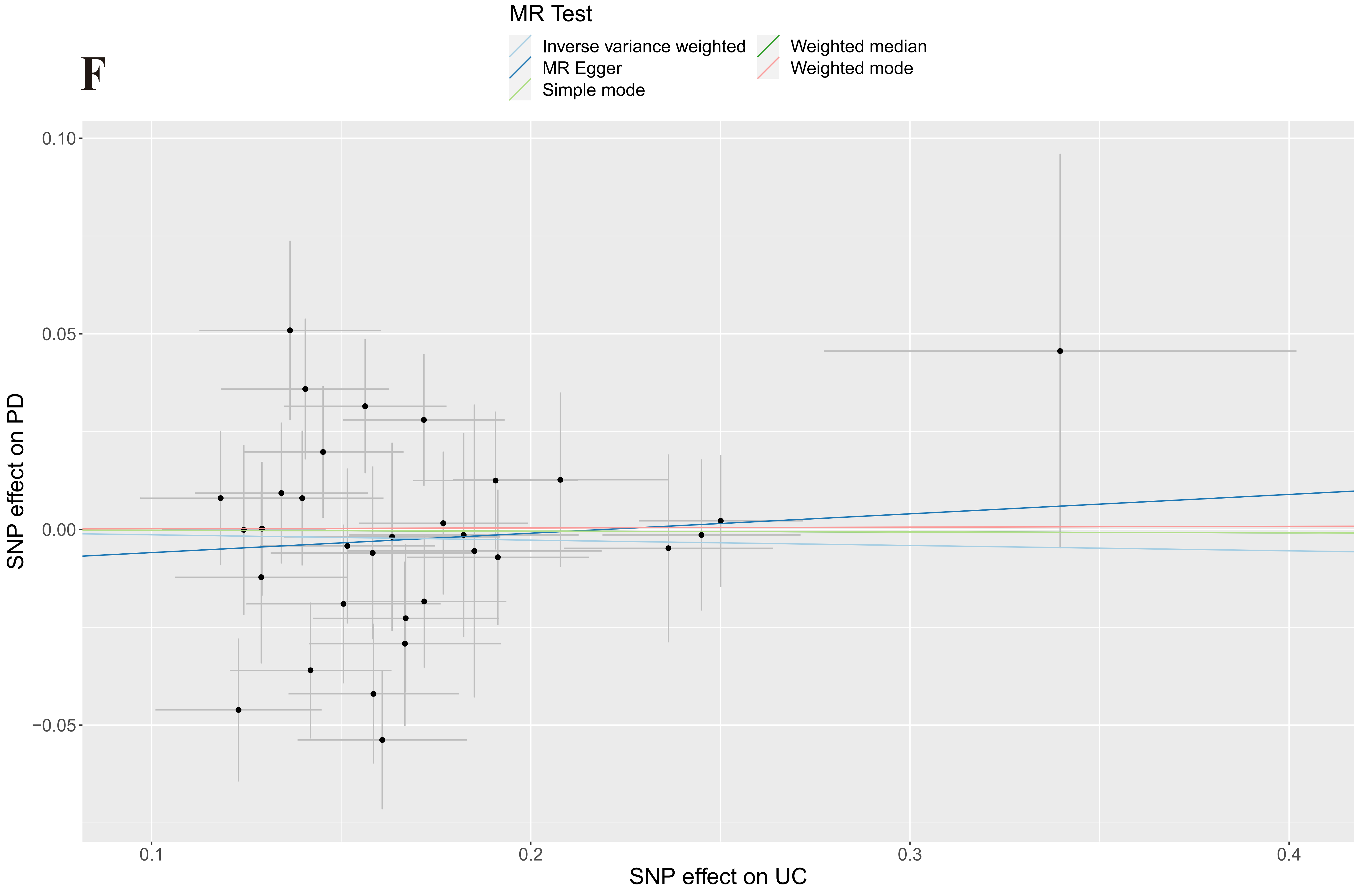
MR Test
Inverse variance weighted
MR Egger
Simple mode
Weighted median
Weighted mode
0.10
0.05
0.00
−0.05
0.1
0.2
0.3
0.4
SNP effect on PD
SNP effect on UC

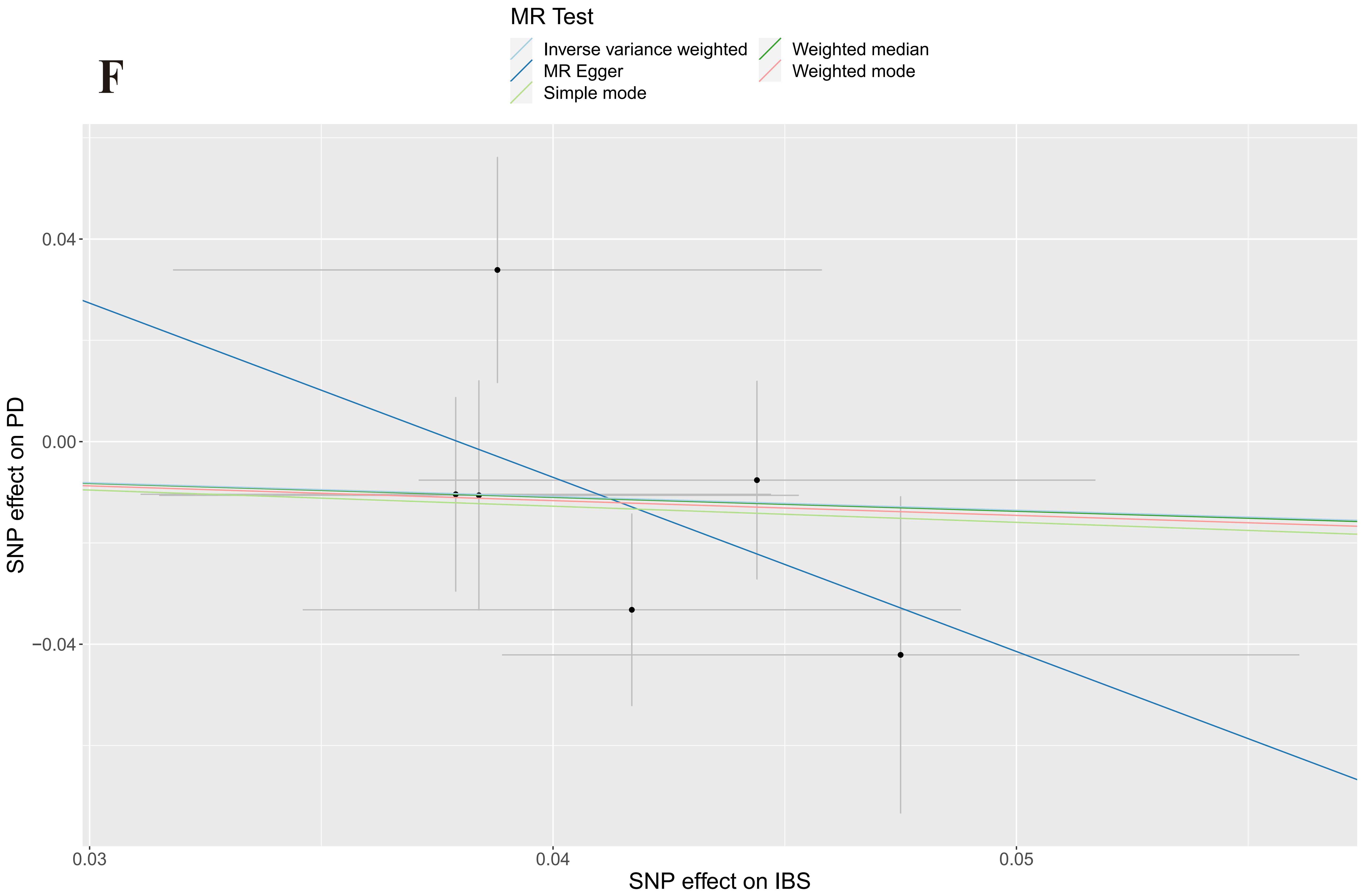
F
MR Test
Inverse variance weighted
MR Egger
Simple mode
Weighted median
Weighted mode
SNP effect on PD
0.04
0.00
−0.04
0.03
0.04
0.05
SNP effect on IBS

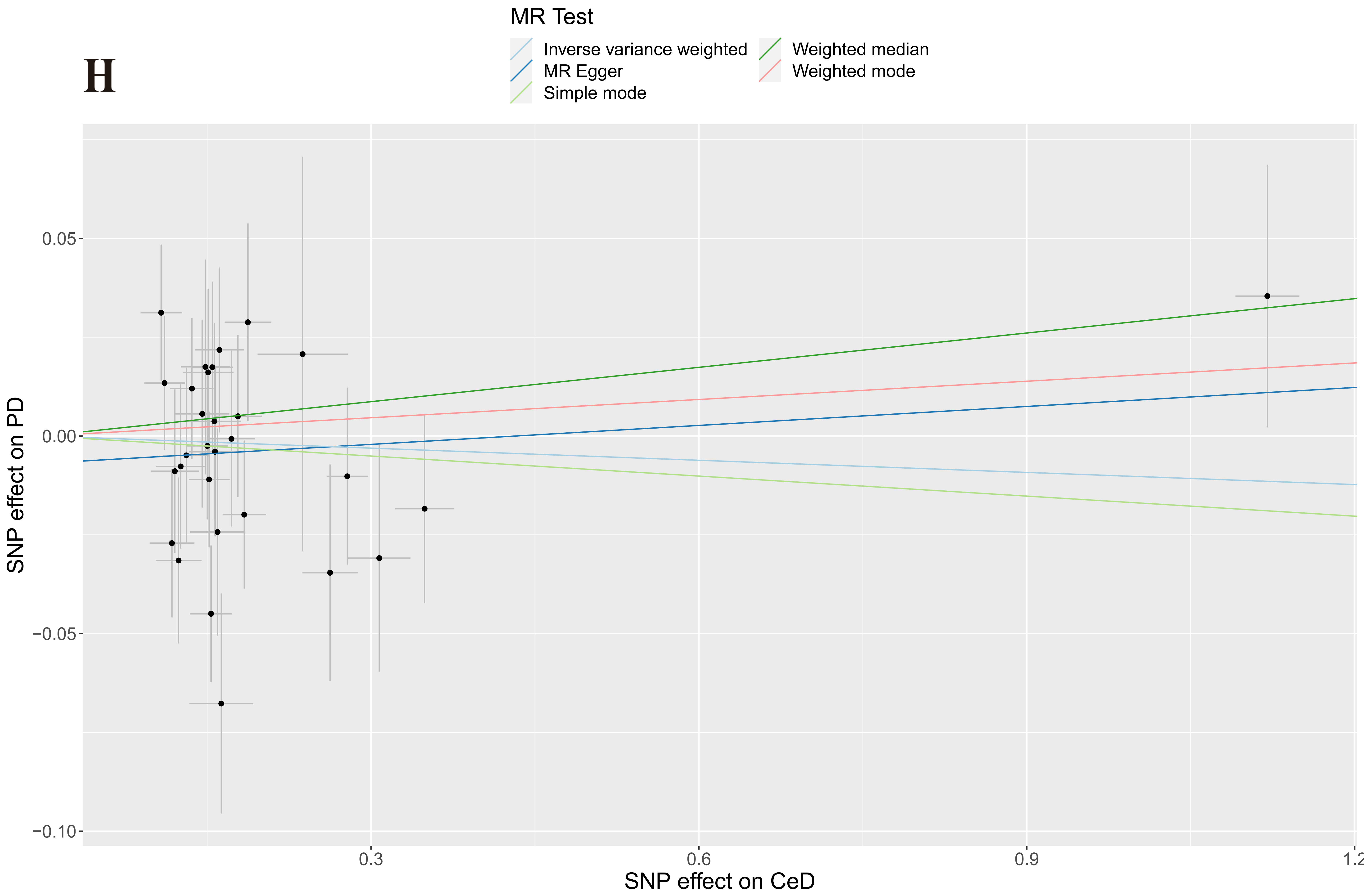

H
MR Test
Inverse variance weighted
MR Egger
Simple mode
Weighted median
Weighted mode
0.05
0.00
−0.05
−0.10
0.3
0.6
0.9
1.2
SNP effect on CeD
SNP effect on PD

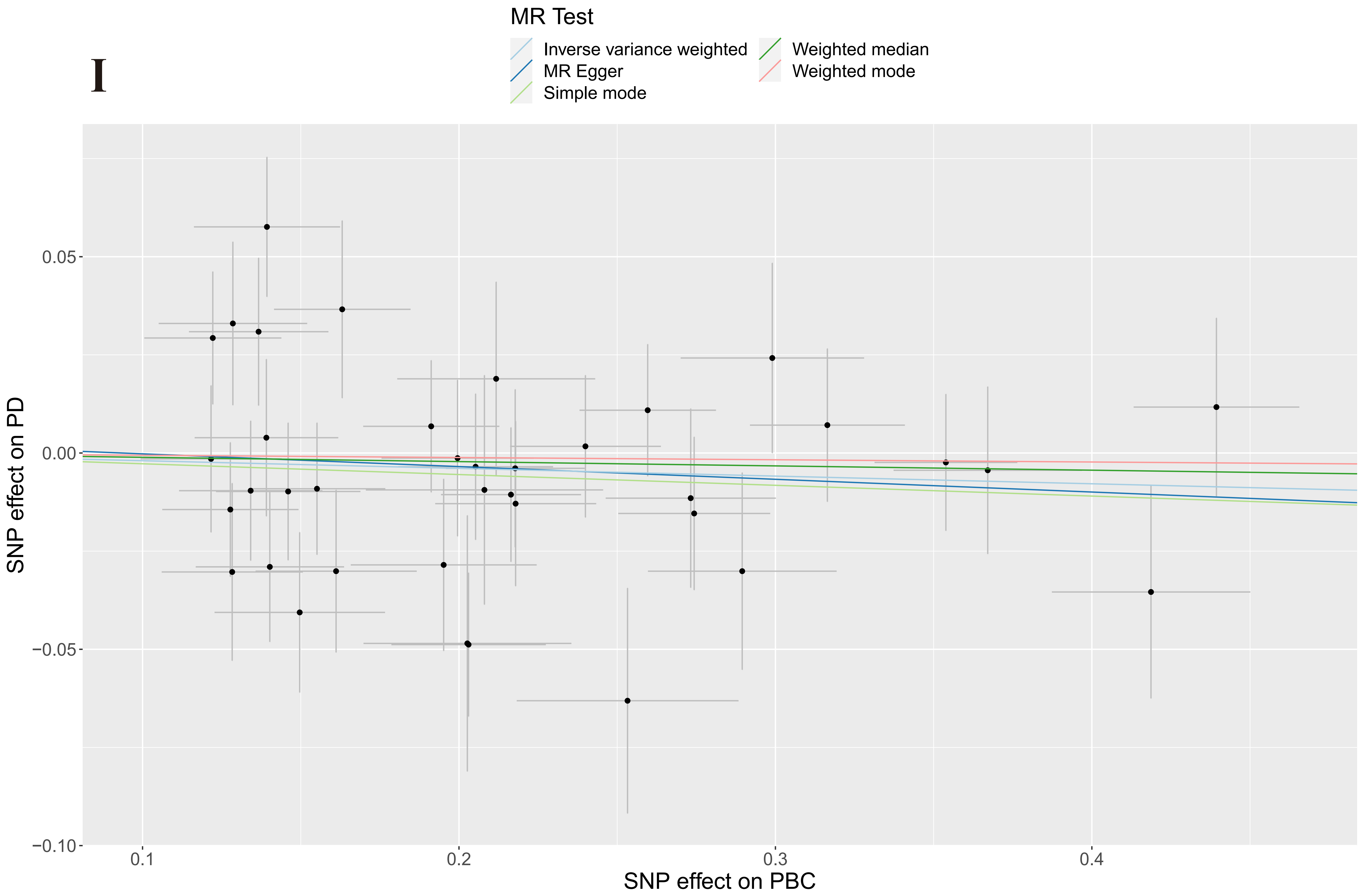
I
MR Test
Inverse variance weighted
MR Egger
Simple mode
Weighted median
Weighted mode
SNP effect on PD
0.05
0.00
−0.05
−0.10
0.1
0.2
0.3
0.4
SNP effect on PBC

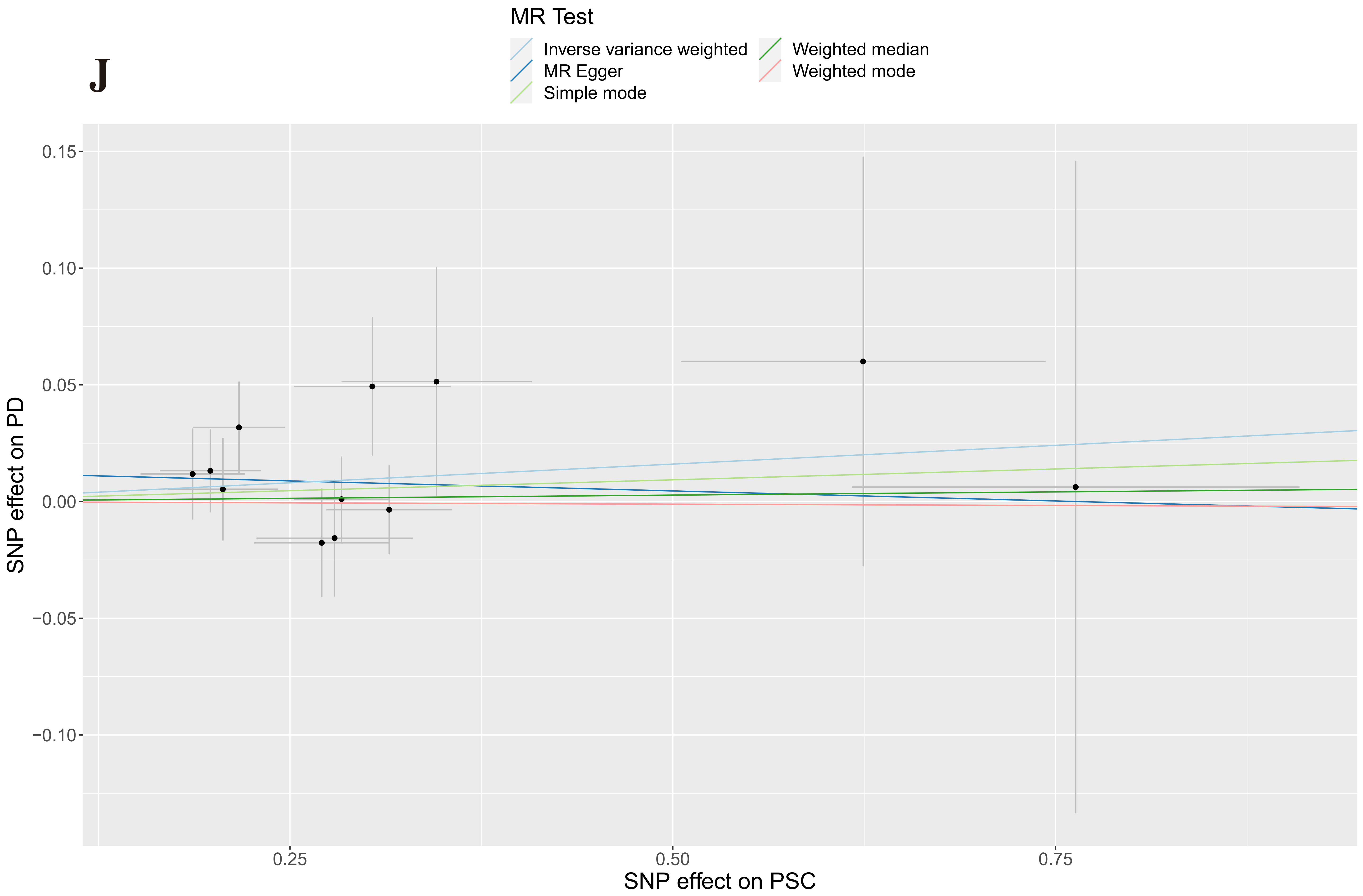

J
MR Test
Inverse variance weighted
MR Egger
Simple mode
Weighted median
Weighted mode
0.15
0.10
0.05
0.00
−0.05
−0.10
SNP effect on PD
0.25
0.50
0.75
SNP effect on PSC

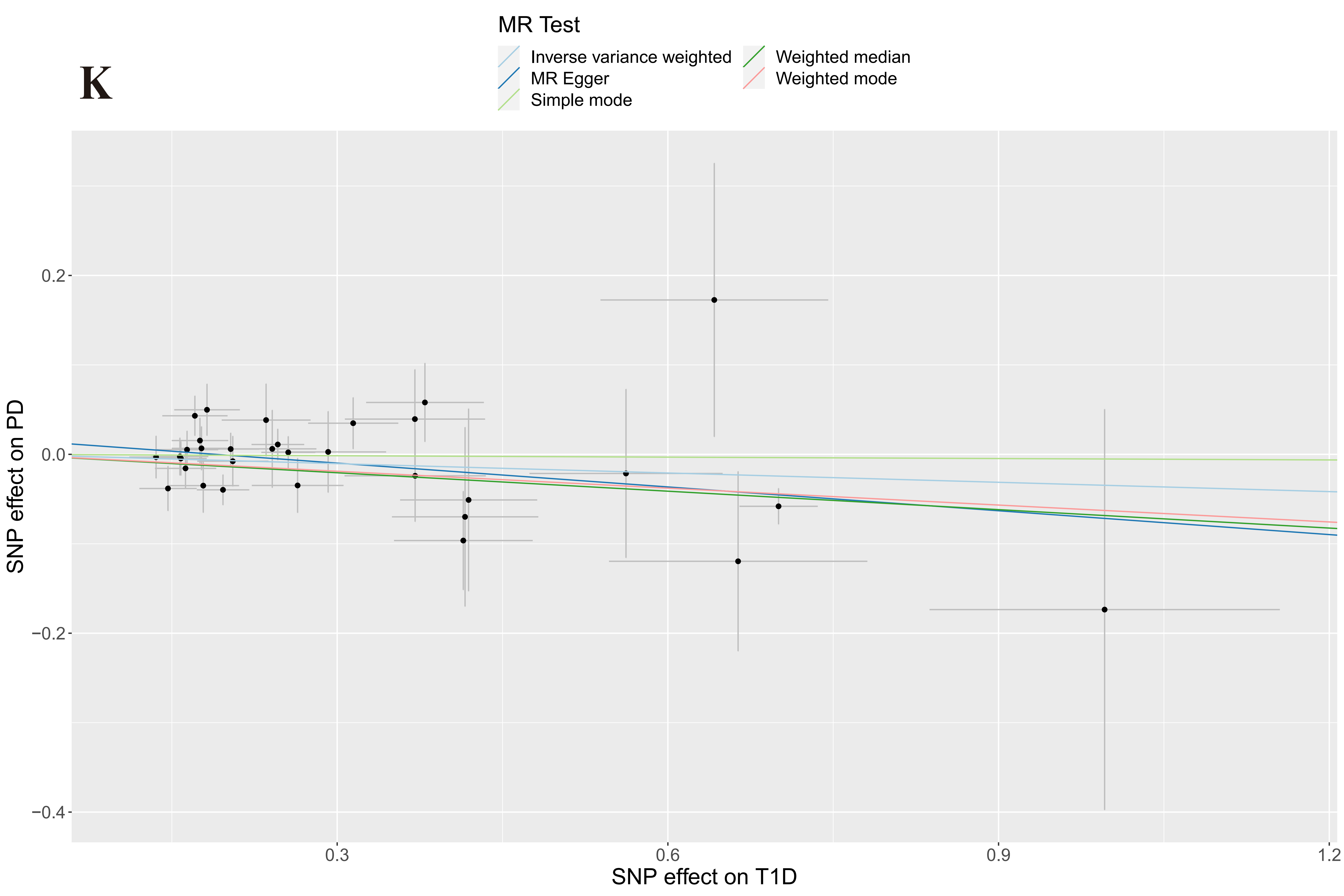

K
MR Test
Inverse variance weighted
MR Egger
Simple mode
Weighted median
Weighted mode
SNP effect on PD
0.2
0.0
−0.2
−0.4
SNP effect on T1D
0.3
0.6
0.9
1.2

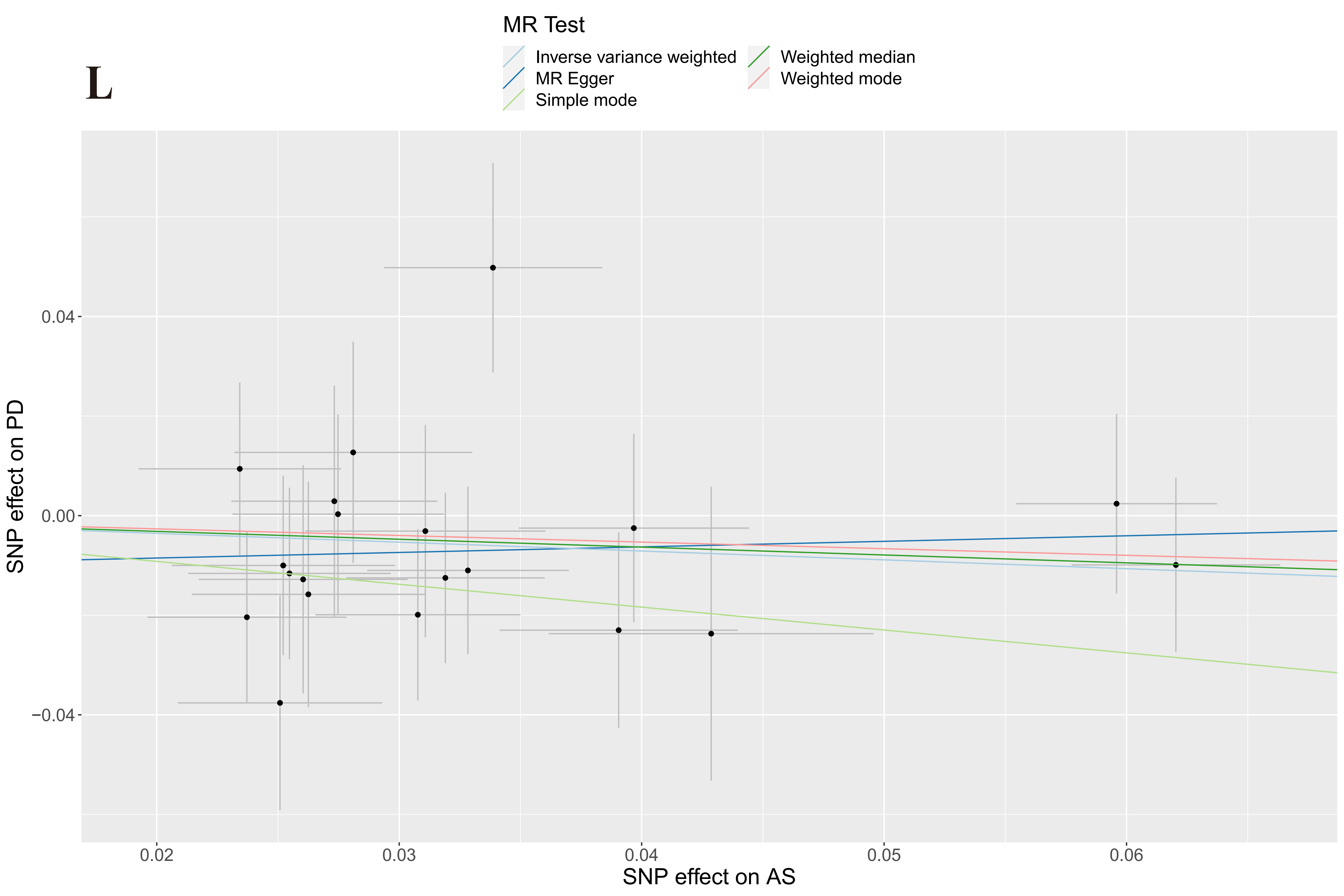
L
MR Test
Inverse variance weighted
MR Egger
Simple mode
Weighted median
Weighted mode
SNP effect on PD
0.04
0.00
−0.04
SNP effect on AS
0.02
0.03
0.04
0.05
0.06

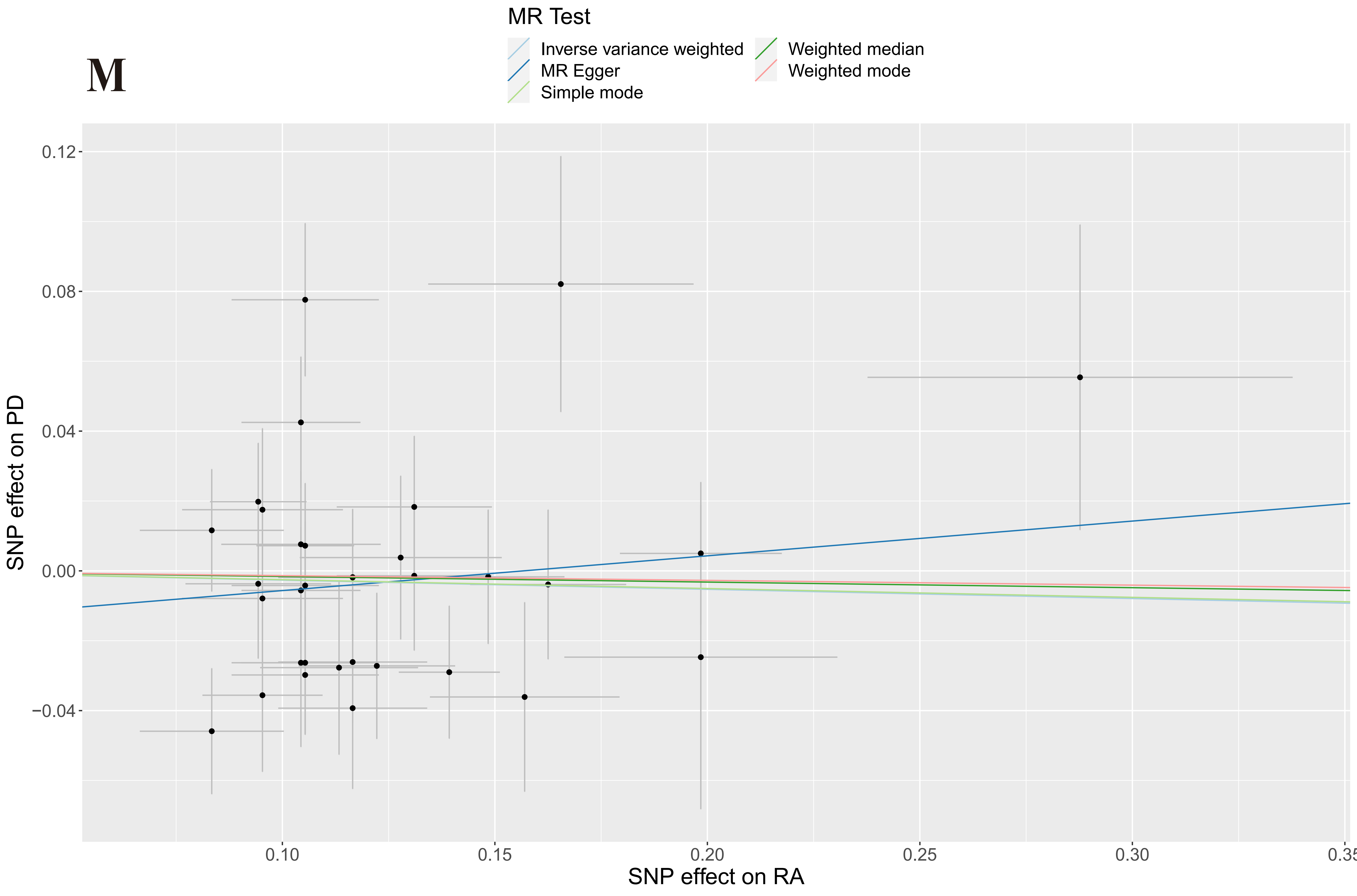
M
MR Test
Inverse variance weighted
MR Egger
Simple mode
Weighted median
Weighted mode
0.12
0.08
0.04
0.00
−0.04
SNP effect on PD
0.10
0.15
0.20
0.25
0.30
0.35
SNP effect on RA

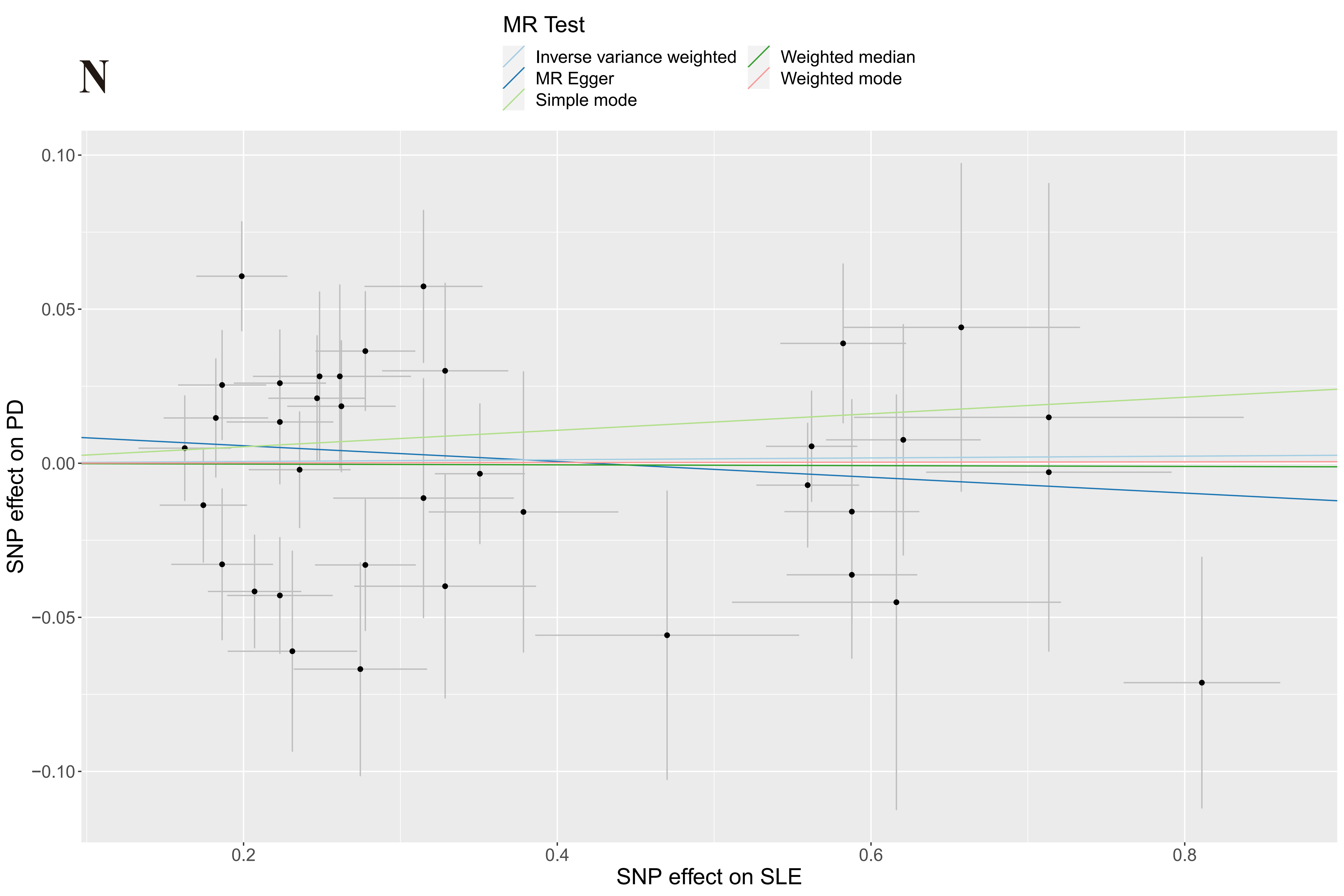

N
MR Test
Inverse variance weighted
MR Egger
Simple mode
Weighted median
Weighted mode
0.10
0.05
0.00
−0.05
−0.10
SNP effect on PD
0.2
0.4
0.6
0.8
SNP effect on SLE

O

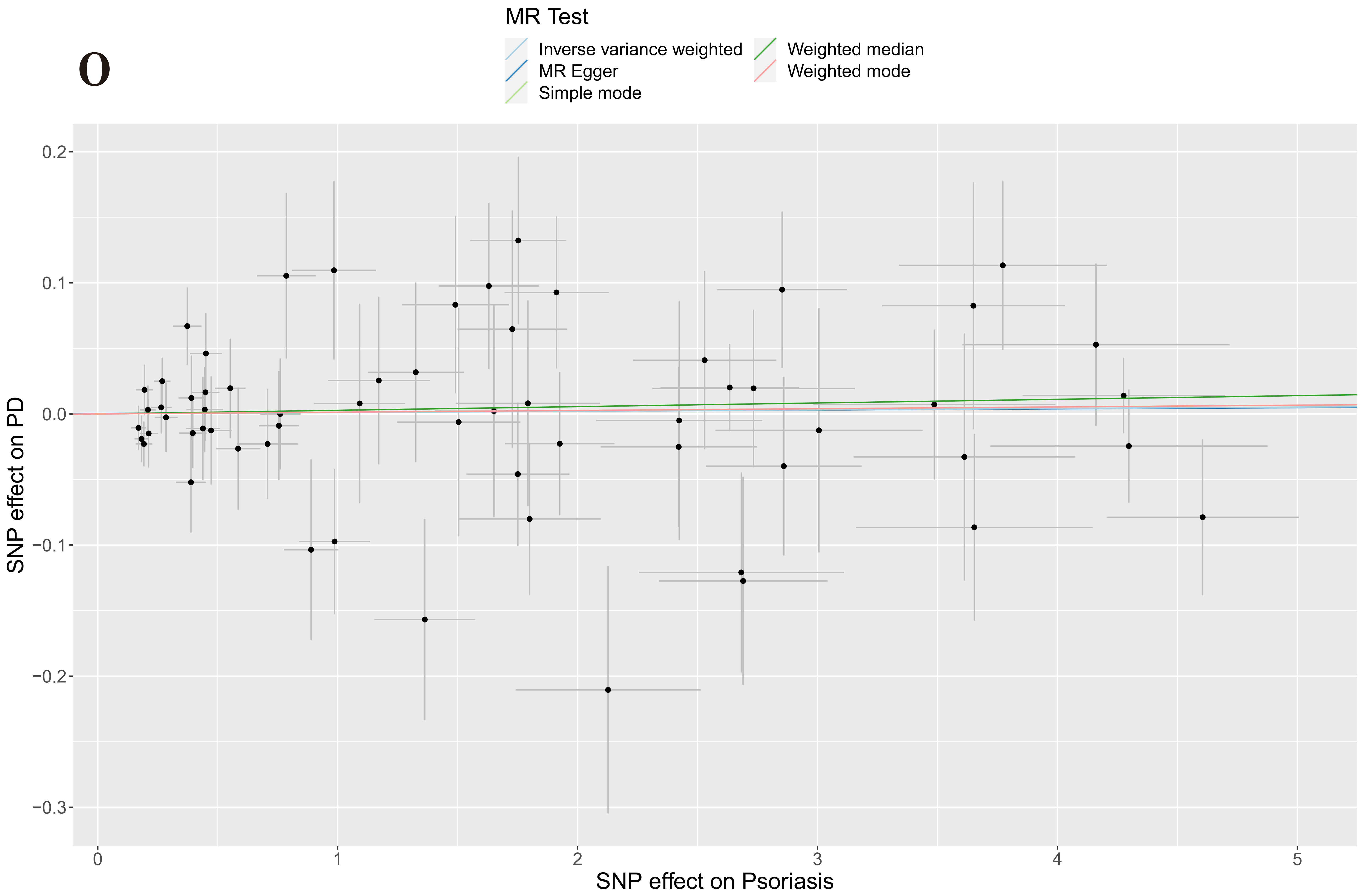

MR Test
Inverse variance weighted
MR Egger
Simple mode
Weighted median
Weighted mode
0.2
0.1
0.0
−0.1
−0.2
−0.3
SNP effect on PD
0
1
2
3
4
5
SNP effect on Psoriasis

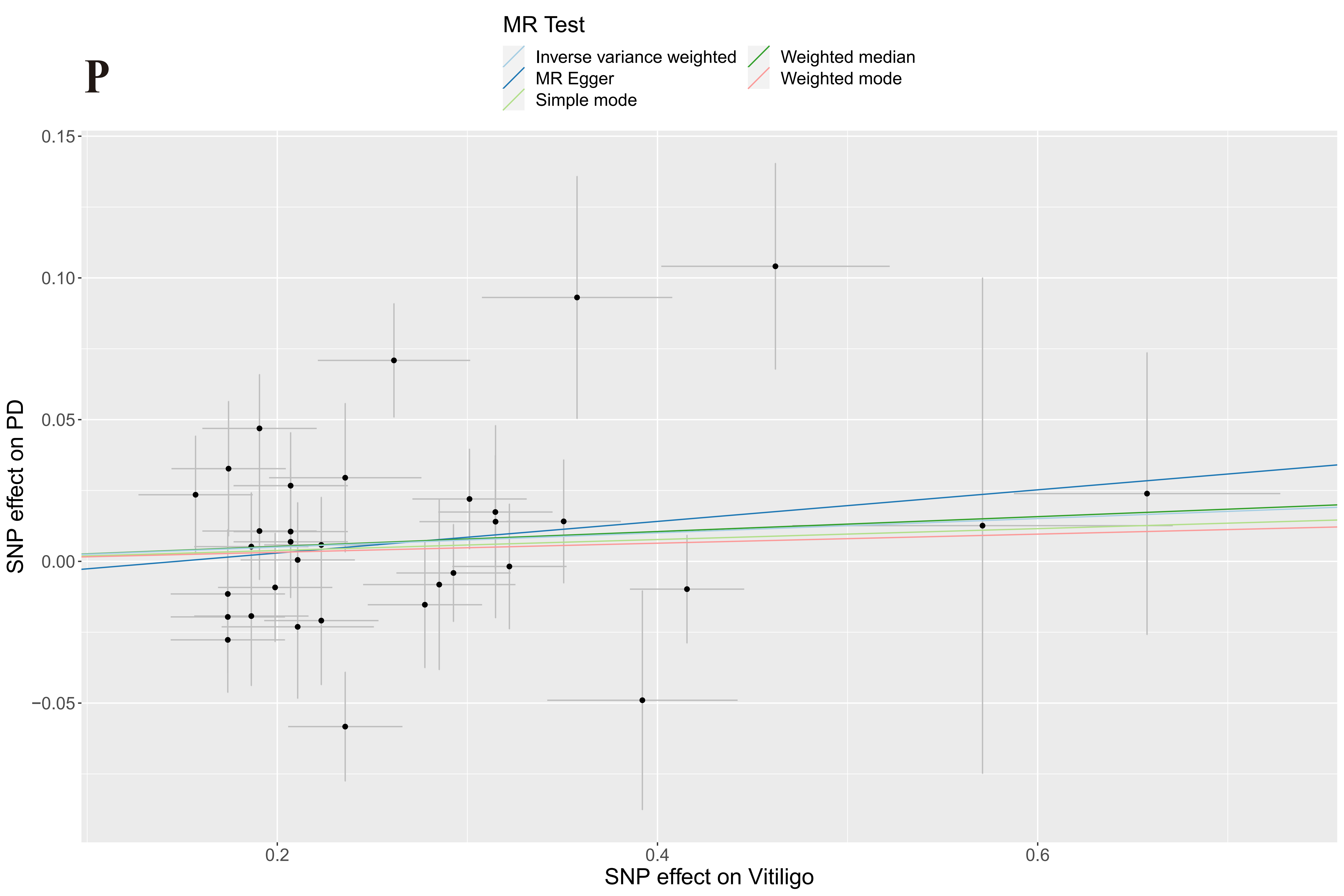
P
MR Test
Inverse variance weighted
MR Egger
Simple mode
Weighted median
Weighted mode
0.15
0.10
0.05
0.00
−0.05
SNP effect on PD
0.2
0.4
0.6
SNP effect on Vitiligo
