# Supplementary material for: Investigation of the causal association between Parkinson’s disease and autoimmune disorders: a bidirectional Mendelian randomization study
Source: Front Immunol. 2024 May 7;15:1370831. doi: 10.3389/fimmu.2024.1370831 (PMC11106379; doi:10.3389/fimmu.2024.1370831)
Supplement: Supplementary Figure 4 — Leave-one-out plots of MR tests assessing the effect of AIDs on PD. (A) MS on PD; (B) MG on PD; (C) Asthma on PD; (D) IBD on PD; (E) CD on PD; (F) UC on PD; (G) IBS on PD; (H) CeD on PD; (I) PBC on PD; (J) PSC on PD; (K) T1D on PD; (L) AS on PD; (M) RA on PD; (N) SLE on PD; (O) Psoriasis on PD; (P) Vitiligo on PD. AIDs, autoimmune diseases; PD, Parkinson’s disease; MS, multiple sclerosis; MG, myasthenia gravis; IBD, inflammatory bowel disease; CD, Crohn’s disease; UC, ulcerative colitis; IBS, irritable bowel syndrome; CeD, celiac disease; PBC, primary biliary cirrhosis; PSC, primary sclerosing cholangitis; T1D, type 1 diabetes; AS, ankylosing spondylitis; RA, rheumatoid arthritis; SLE, systemic lupus erythematosus. [file DataSheet_4.pdf]

Supplementary Figure 4. Leave-one-out plots of MR tests assessing the effect of AIDs on PD.

A

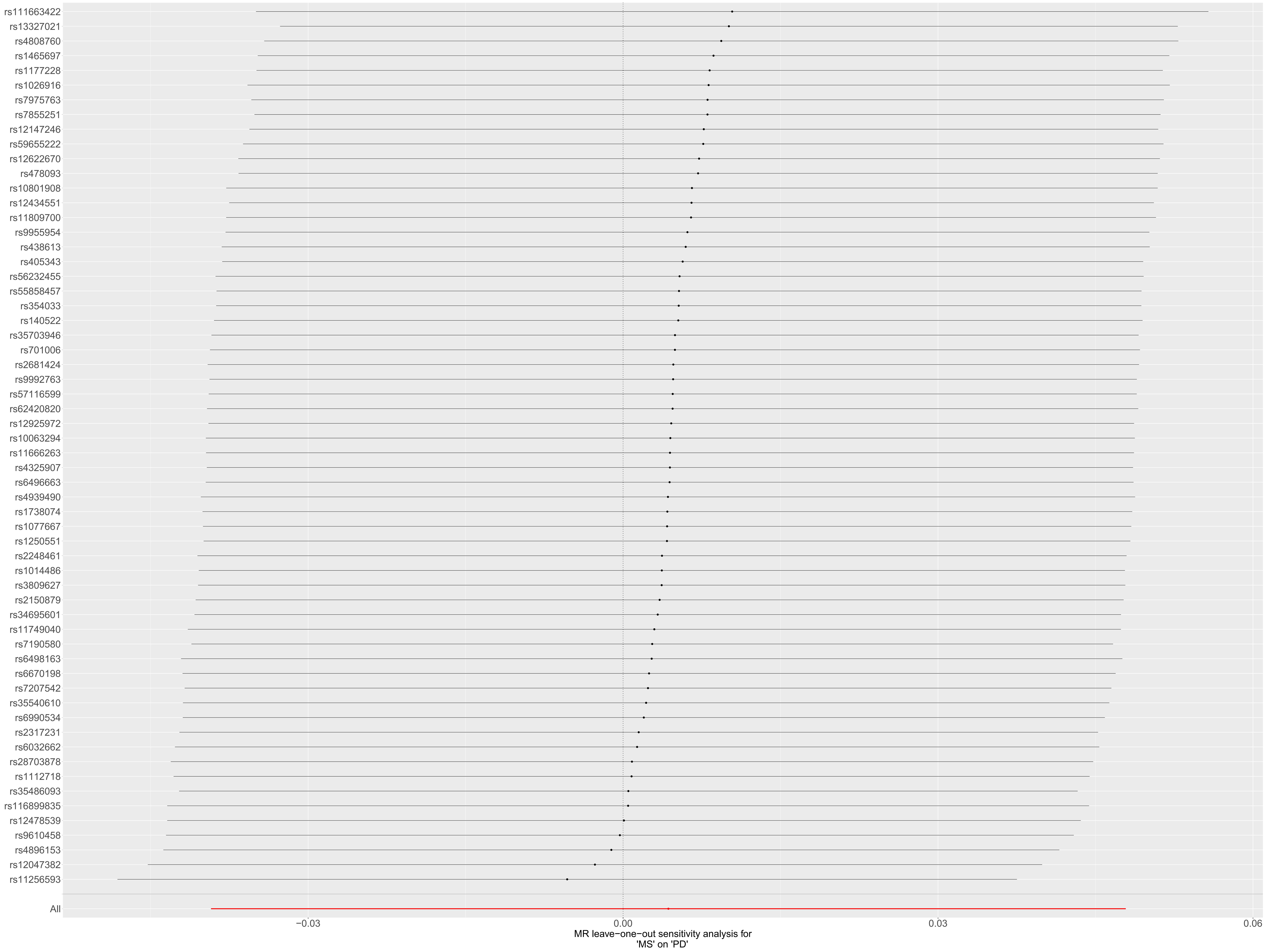

B

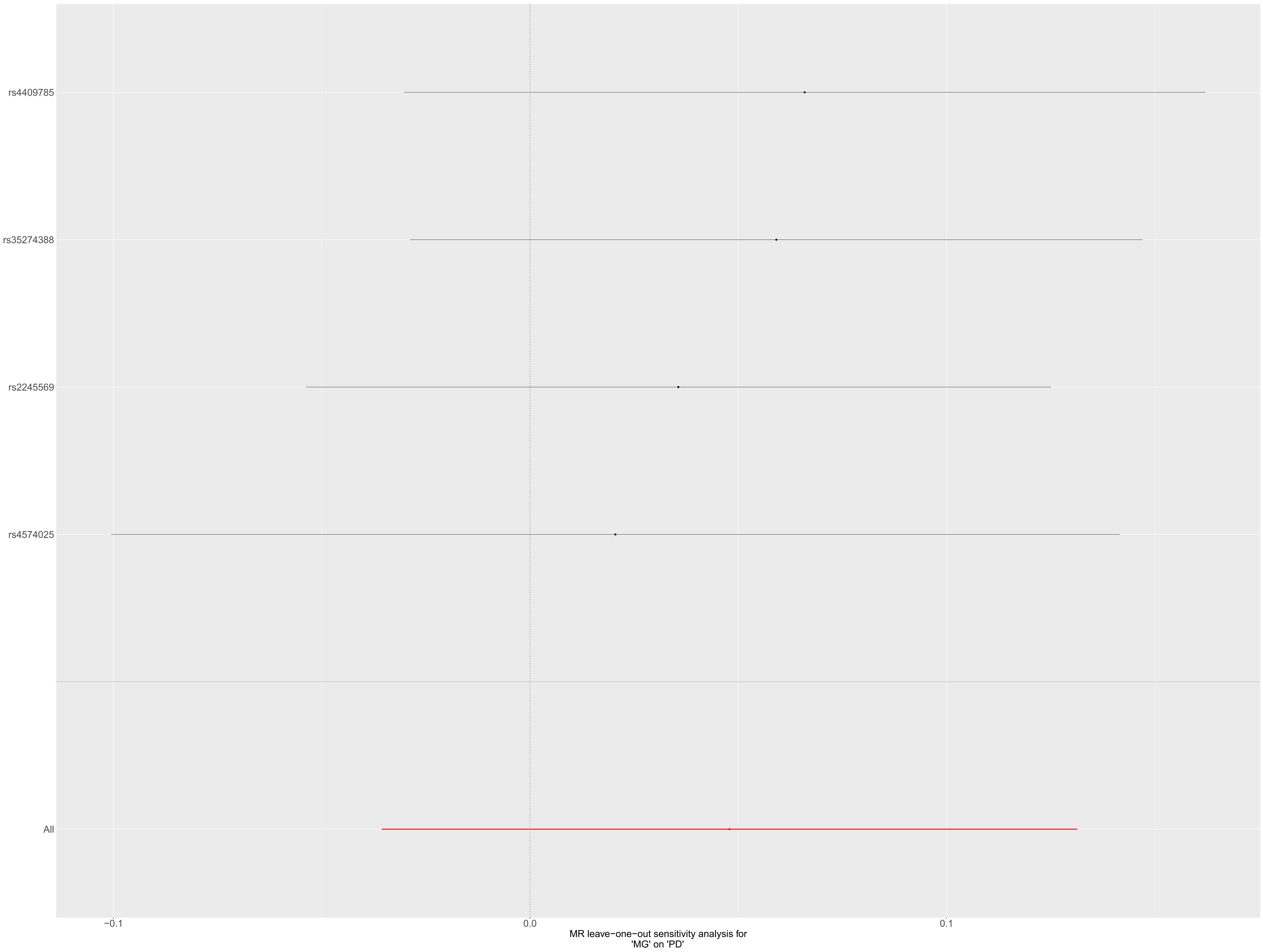
rs4409785
rs35274388
rs2245569
rs4574025
All
−0.1
0.0
0.1
MR leave−one−out sensitivity analysis for
'MG' on 'PD'

C

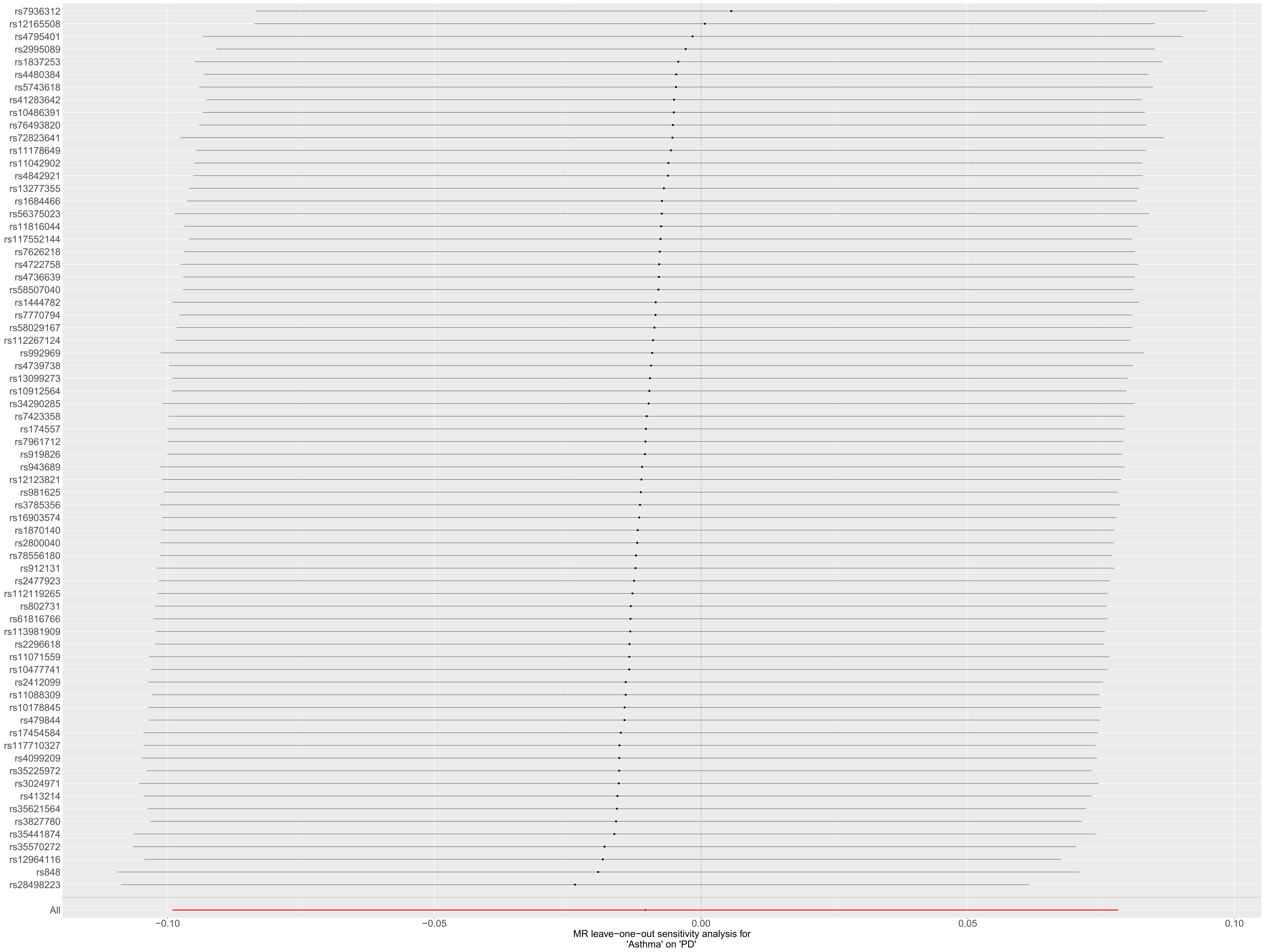
rs7936312
rs12165508
rs4795401
rs2995089
rs1837253
rs4480384
rs5743618
rs41283642
rs10486391
rs76493820
rs72823641
rs11178649
rs11042902
rs4842921
rs13277355
rs1684466
rs56375023
rs11816044
rs117552144
rs7626218
rs4722758
rs4736639
rs58507040
rs1444782
rs7770794
rs58029167
rs112267124
rs992969
rs4739738
rs13099273
rs10912564
rs34290285
rs7423358
rs174557
rs7961712
rs919826
rs943689
rs12123821
rs981625
rs3785356
rs16903574
rs1870140
rs2800040
rs78556180
rs912131
rs2477923
rs112119265
rs802731
rs61816766
rs113981909
rs2296618
rs11071559
rs10477741
rs2412099
rs11088309
rs10178845
rs479844
rs17454584
rs117710327
rs4099209
rs35225972
rs3024971
rs413214
rs35621564
rs3827780
rs35441874
rs35570272
rs12964116
rs848
rs28498223
All
−0.10
−0.05
0.00
0.05
0.10
MR leave−one−out sensitivity analysis for
'Asthma' on 'PD'

D

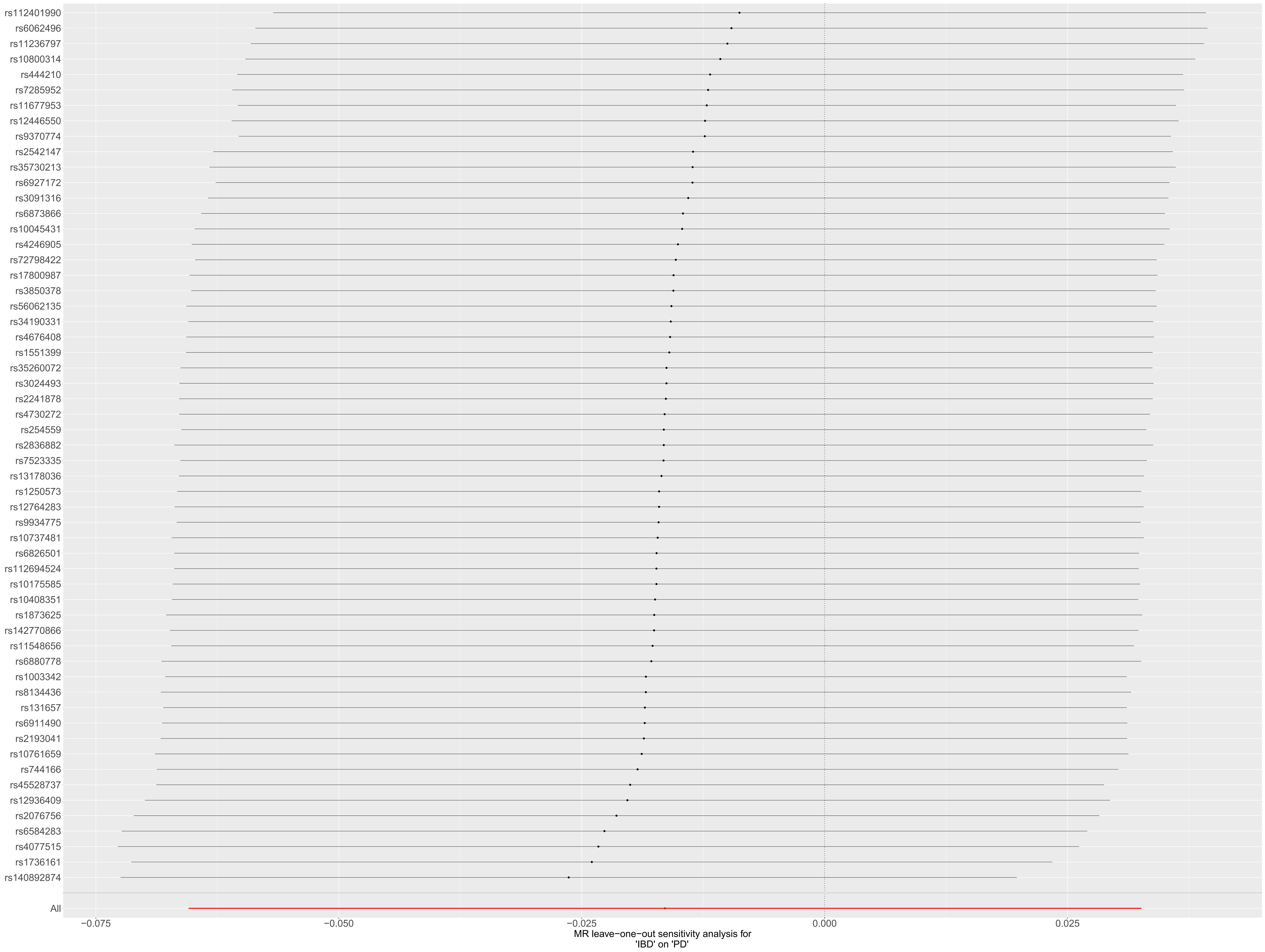
rs112401990
rs6062496
rs11236797
rs10800314
rs444210
rs7285952
rs11677953
rs12446550
rs9370774
rs2542147
rs35730213
rs6927172
rs3091316
rs6873866
rs10045431
rs4246905
rs72798422
rs17800987
rs3850378
rs56062135
rs34190331
rs4676408
rs1551399
rs35260072
rs3024493
rs2241878
rs4730272
rs254559
rs2836882
rs7523335
rs13178036
rs1250573
rs12764283
rs9934775
rs10737481
rs6826501
rs112694524
rs10175585
rs10408351
rs1873625
rs142770866
rs11548656
rs6880778
rs1003342
rs8134436
rs131657
rs6911490
rs2193041
rs10761659
rs744166
rs45528737
rs12936409
rs2076756
rs6584283
rs4077515
rs1736161
rs140892874
All
−0.075
−0.050
−0.025
0.000
0.025
MR leave−one−out sensitivity analysis for
'IBD' on 'PD'

E

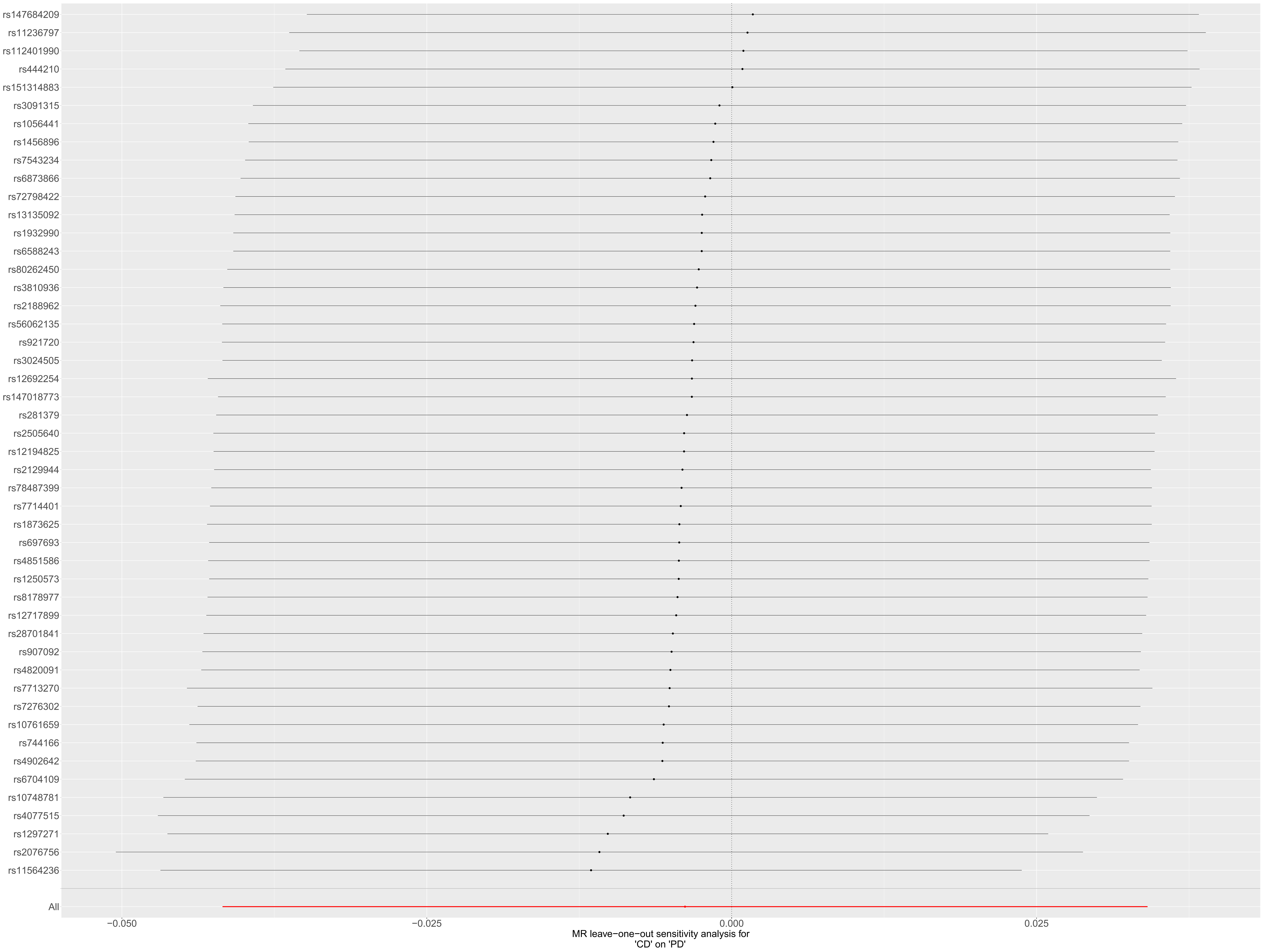
rs147684209
rs11236797
rs112401990
rs444210
rs151314883
rs3091315
rs1056441
rs1456896
rs7543234
rs6873866
rs72798422
rs13135092
rs1932990
rs6588243
rs80262450
rs3810936
rs2188962
rs56062135
rs921720
rs3024505
rs12692254
rs147018773
rs281379
rs2505640
rs12194825
rs2129944
rs78487399
rs7714401
rs1873625
rs697693
rs4851586
rs1250573
rs8178977
rs12717899
rs28701841
rs907092
rs4820091
rs7713270
rs7276302
rs10761659
rs744166
rs4902642
rs6704109
rs10748781
rs4077515
rs1297271
rs2076756
rs11564236
All
−0.050
−0.025
0.000
0.025
MR leave−one−out sensitivity analysis for
'CD' on 'PD'

F

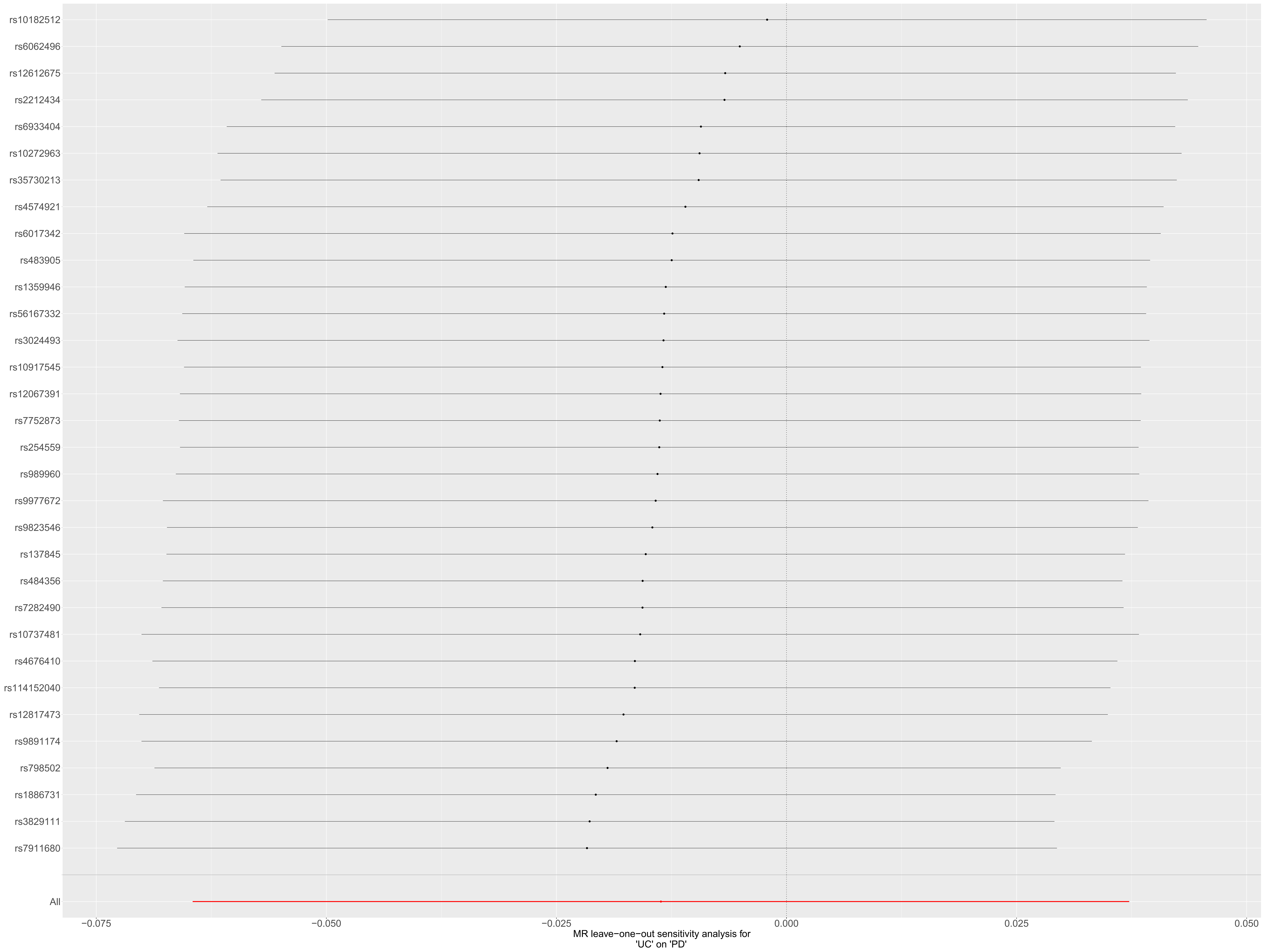
rs10182512
rs6062496
rs12612675
rs2212434
rs6933404
rs10272963
rs35730213
rs4574921
rs6017342
rs483905
rs1359946
rs56167332
rs3024493
rs10917545
rs12067391
rs7752873
rs254559
rs989960
rs9977672
rs9823546
rs137845
rs484356
rs7282490
rs10737481
rs4676410
rs114152040
rs12817473
rs9891174
rs798502
rs1886731
rs3829111
rs7911680
All
−0.075
−0.050
−0.025
0.000
0.025
0.050
MR leave−one−out sensitivity analysis for
'UC' on 'PD'

G

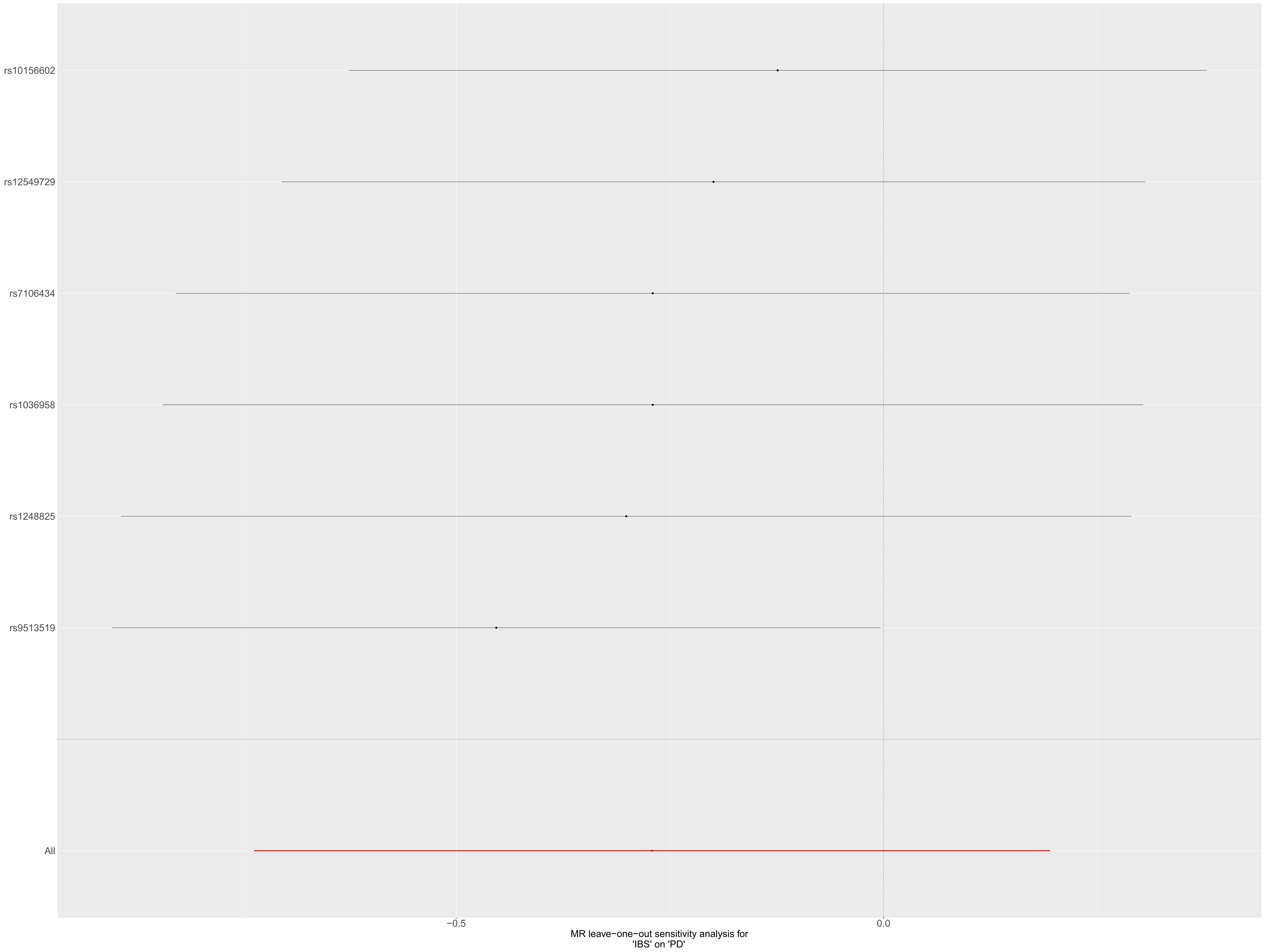
rs10156602
rs12549729
rs7106434
rs1036958
rs1248825
rs9513519
All
−0.5
0.0
MR leave−one−out sensitivity analysis for
'IBS' on 'PD'

H

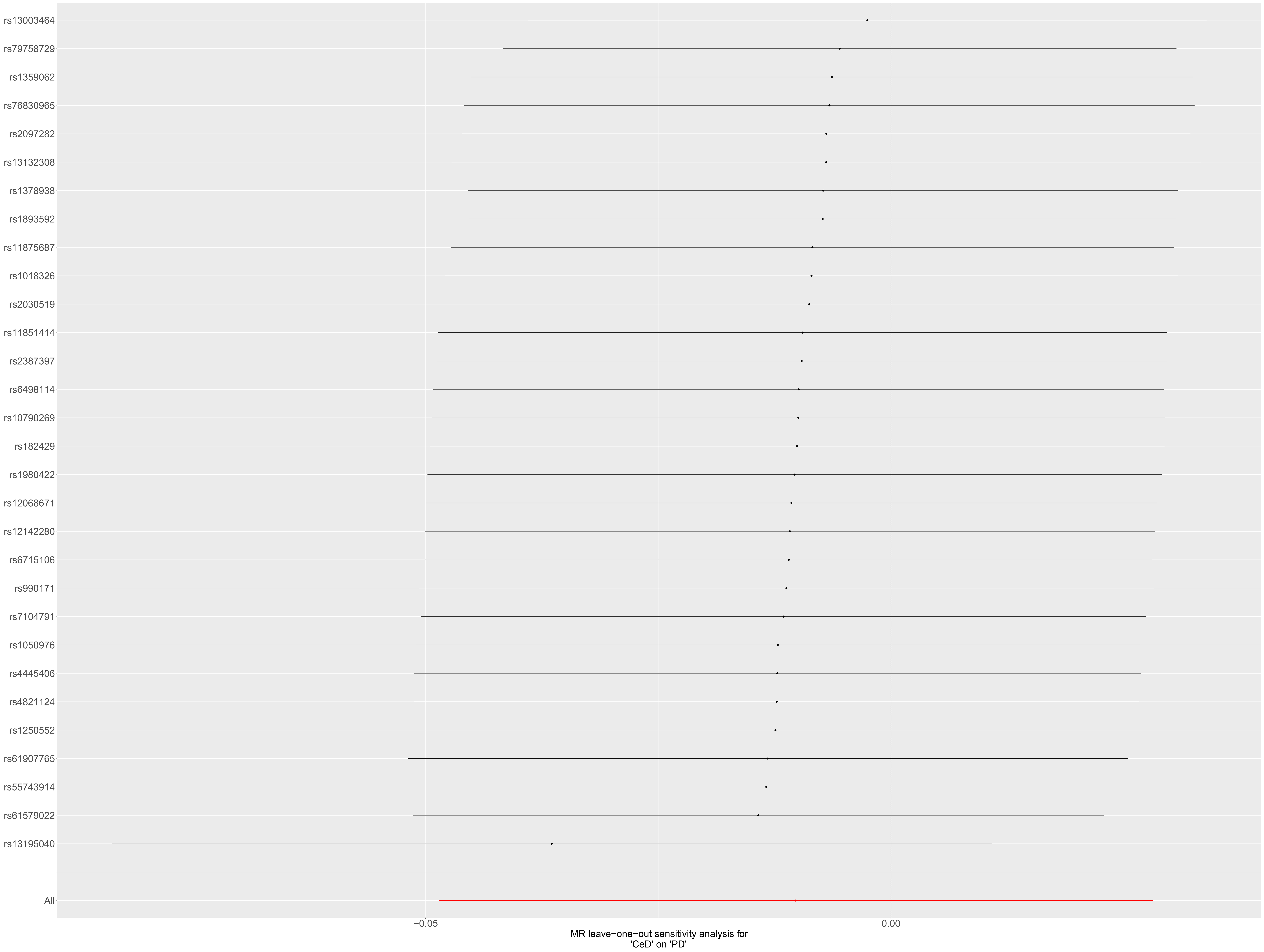

rs13003464
rs79758729
rs1359062
rs76830965
rs2097282
rs13132308
rs1378938
rs1893592
rs11875687
rs1018326
rs2030519
rs11851414
rs2387397
rs6498114
rs10790269
rs182429
rs1980422
rs12068671
rs12142280
rs6715106
rs990171
rs7104791
rs1050976
rs4445406
rs4821124
rs1250552
rs61907765
rs55743914
rs61579022
rs13195040
All
−0.05
0.00
MR leave−one−out sensitivity analysis for
'CeD' on 'PD'

I

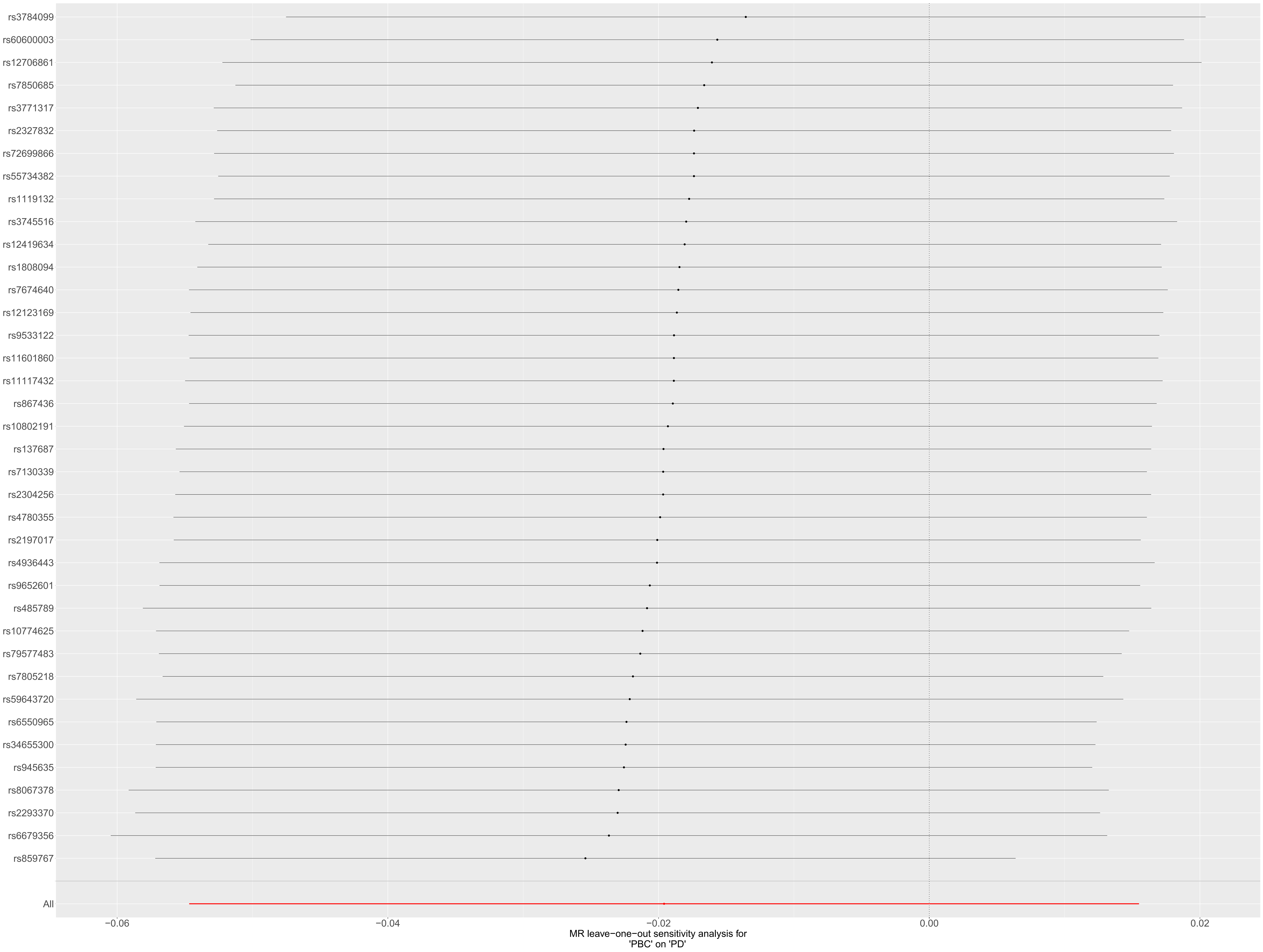

rs3784099
rs60600003
rs12706861
rs7850685
rs3771317
rs2327832
rs72699866
rs55734382
rs1119132
rs3745516
rs12419634
rs1808094
rs7674640
rs12123169
rs9533122
rs11601860
rs11117432
rs867436
rs10802191
rs137687
rs7130339
rs2304256
rs4780355
rs2197017
rs4936443
rs9652601
rs485789
rs10774625
rs79577483
rs7805218
rs59643720
rs6550965
rs34655300
rs945635
rs8067378
rs2293370
rs6679356
rs859767
All
−0.06
−0.04
−0.02
0.00
0.02
MR leave−one−out sensitivity analysis for
'PBC' on 'PD'

J

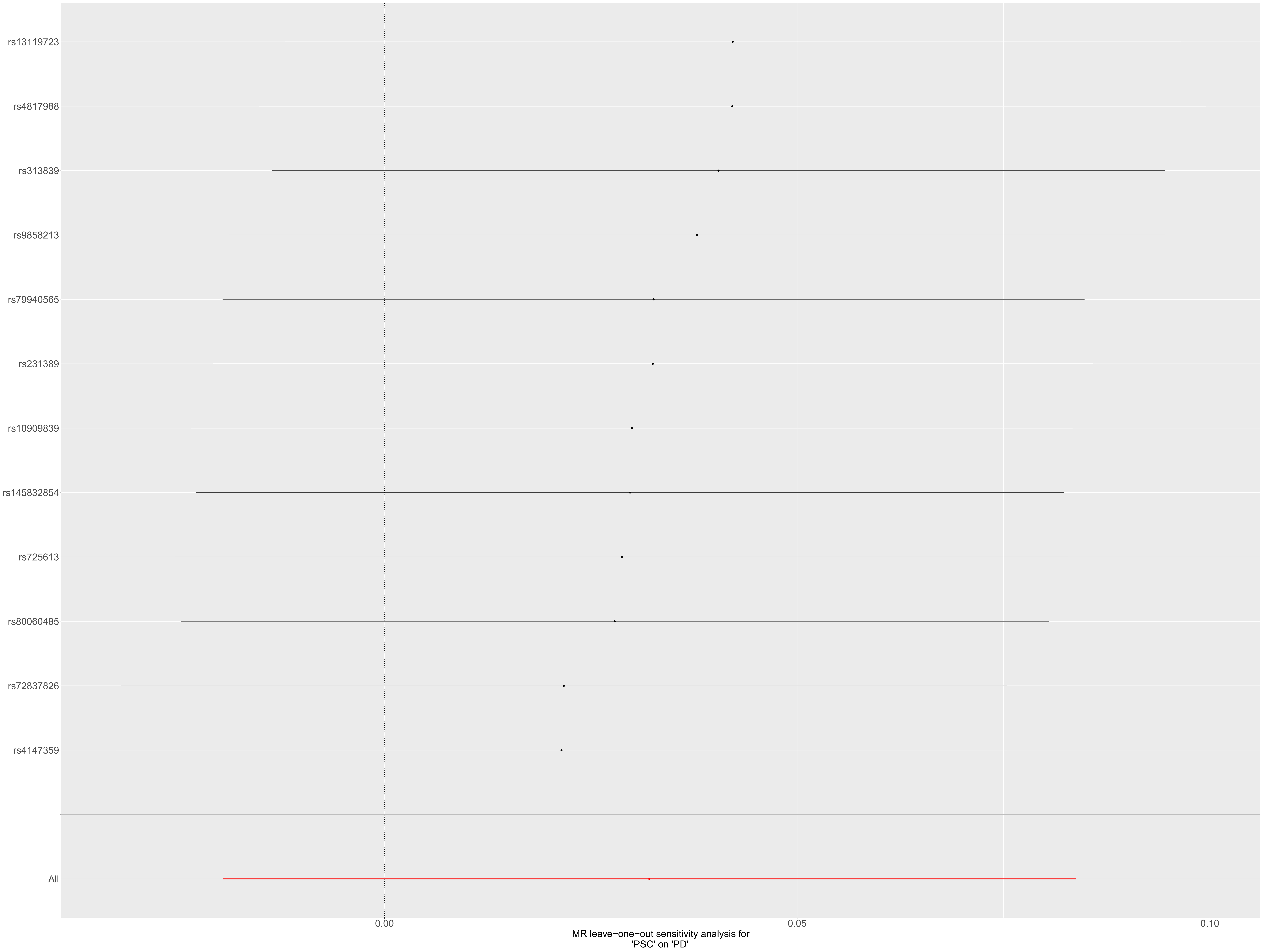
rs13119723
rs4817988
rs313839
rs9858213
rs79940565
rs231389
rs10909839
rs145832854
rs725613
rs80060485
rs72837826
rs4147359
All
0.00
0.05
0.10
MR leave−one−out sensitivity analysis for
'PSC' on 'PD'

K

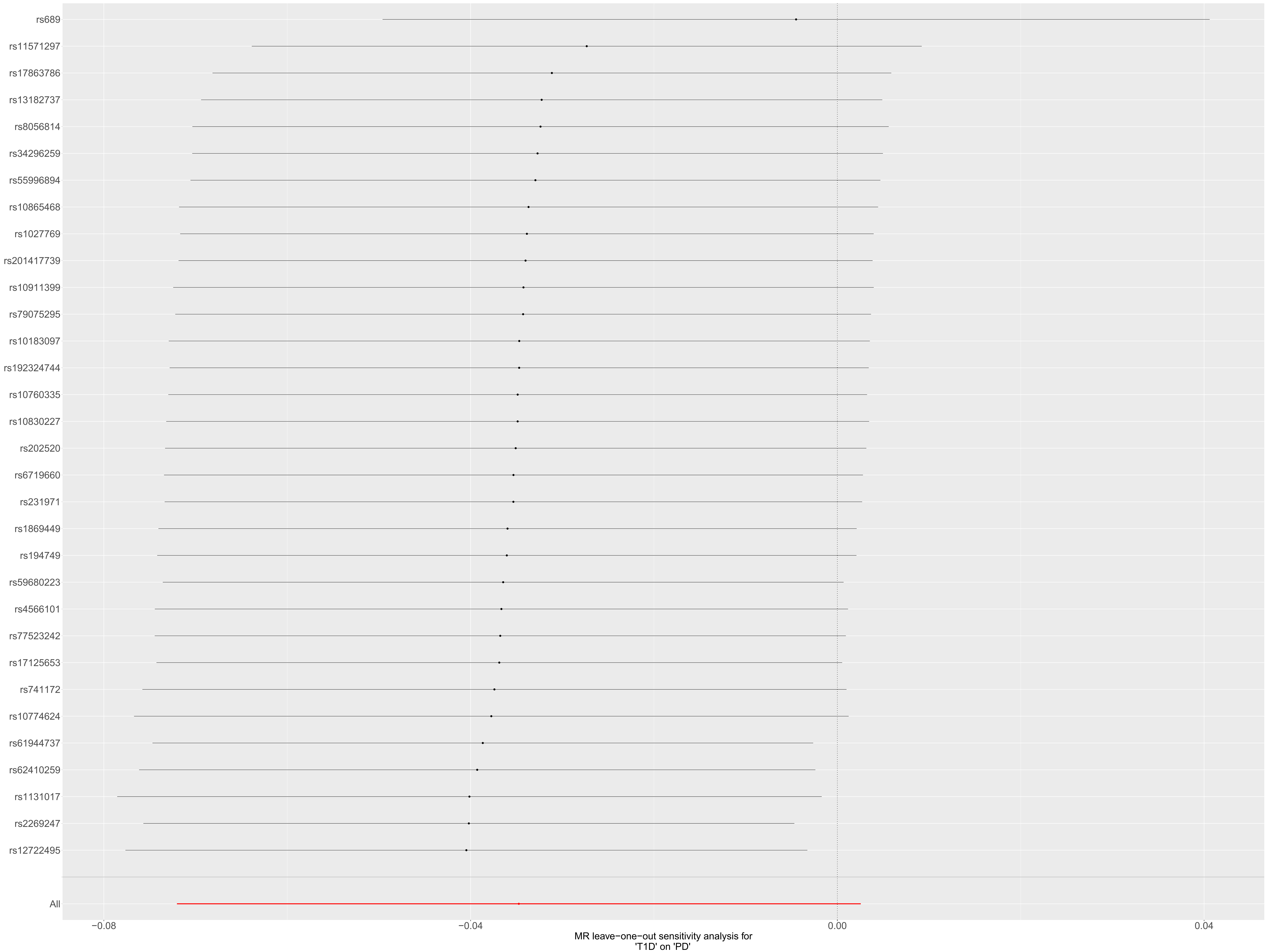
rs689
rs11571297
rs17863786
rs13182737
rs8056814
rs34296259
rs55996894
rs10865468
rs1027769
rs201417739
rs10911399
rs79075295
rs10183097
rs192324744
rs10760335
rs10830227
rs202520
rs6719660
rs231971
rs1869449
rs194749
rs59680223
rs4566101
rs77523242
rs17125653
rs741172
rs10774624
rs61944737
rs62410259
rs1131017
rs2269247
rs12722495
All
−0.08
−0.04
0.00
0.04
MR leave−one−out sensitivity analysis for
'T1D' on 'PD'

L

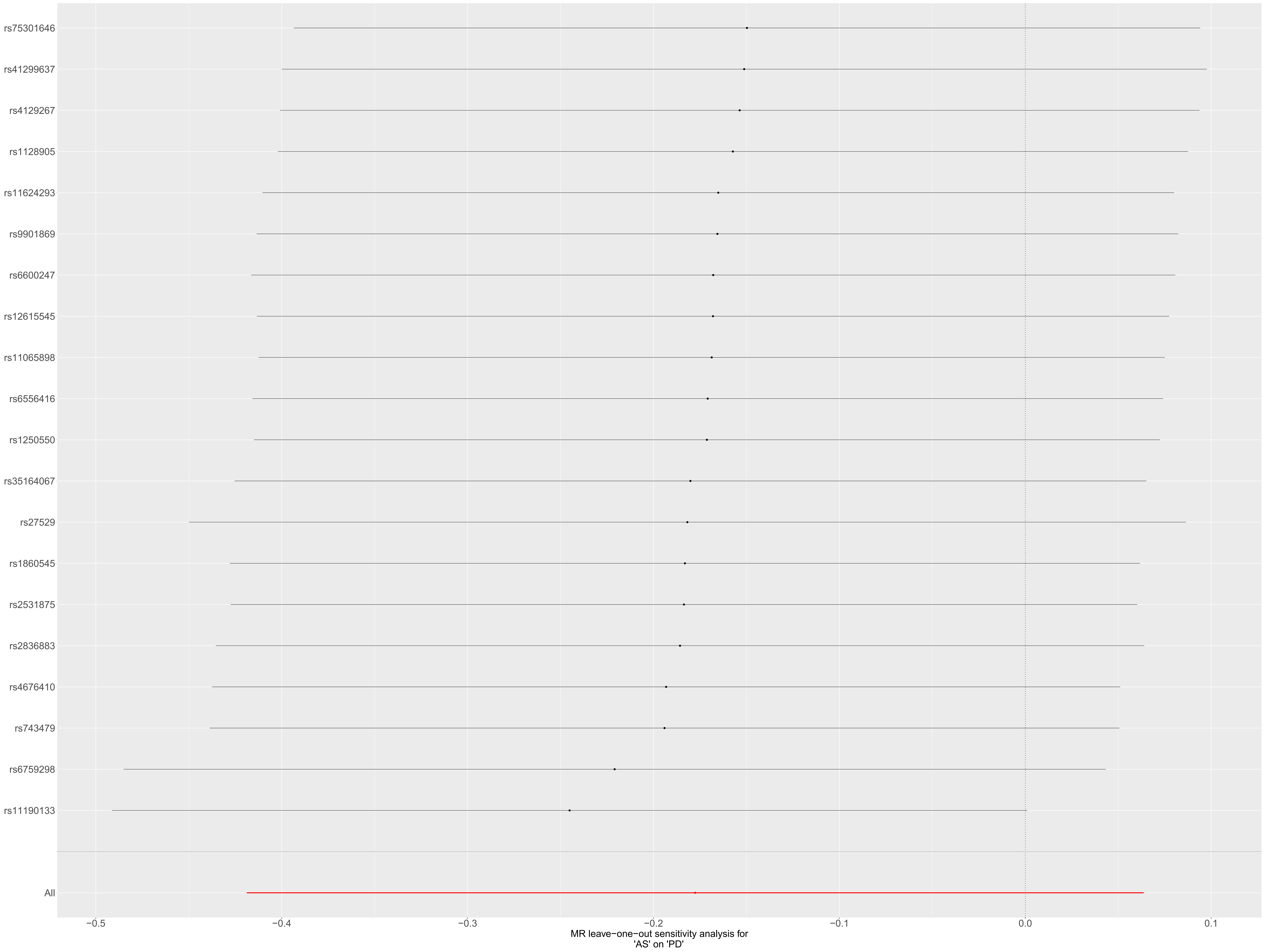
rs75301646
rs41299637
rs4129267
rs1128905
rs11624293
rs9901869
rs6600247
rs12615545
rs11065898
rs6556416
rs1250550
rs35164067
rs27529
rs1860545
rs2531875
rs2836883
rs4676410
rs743479
rs6759298
rs11190133
All
−0.5
−0.4
−0.3
−0.2
−0.1
0.0
0.1
MR leave−one−out sensitivity analysis for
'AS' on 'PD'

M

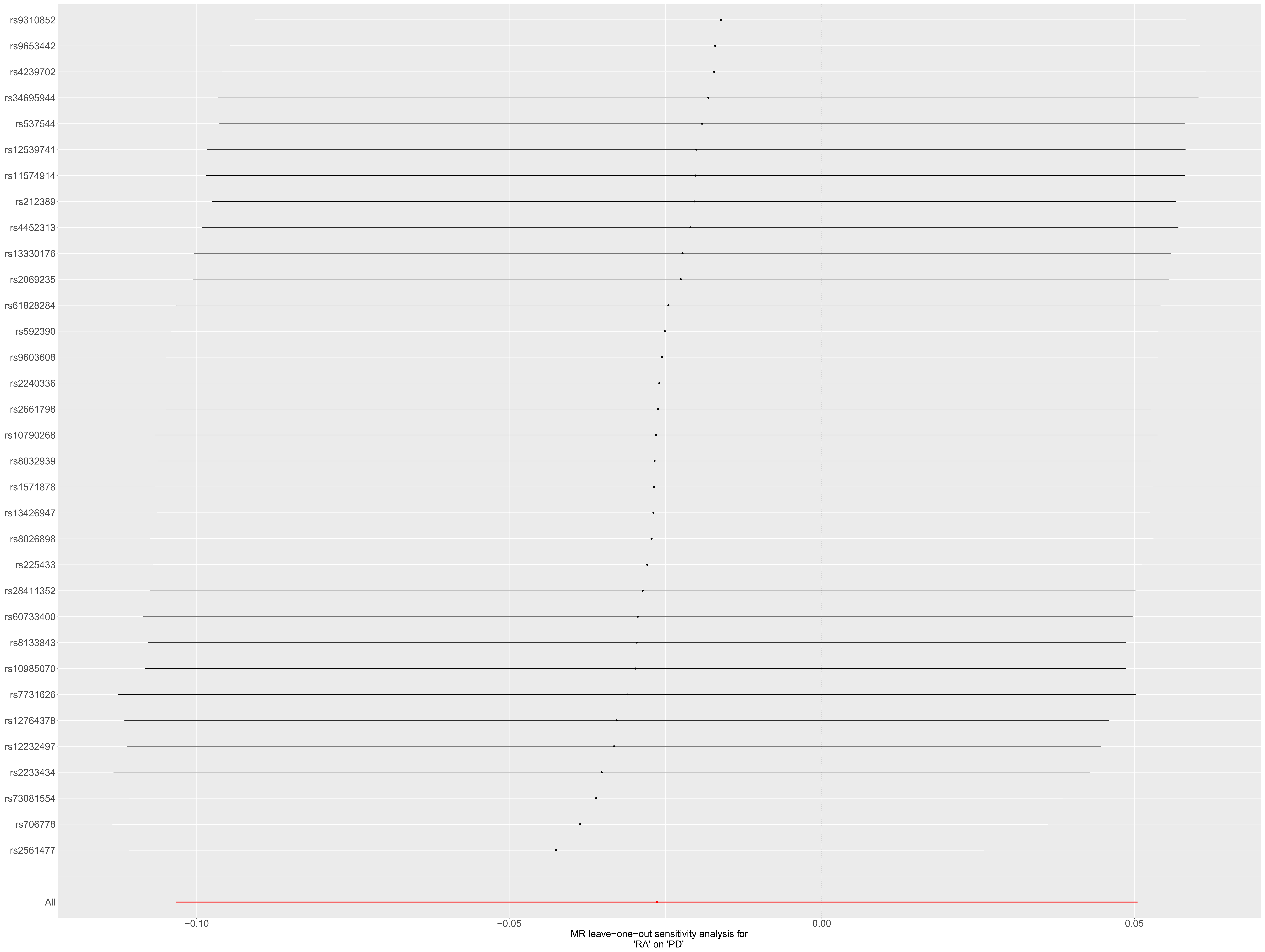
rs9310852
rs9653442
rs4239702
rs34695944
rs537544
rs12539741
rs11574914
rs212389
rs4452313
rs13330176
rs2069235
rs61828284
rs592390
rs9603608
rs2240336
rs2661798
rs10790268
rs8032939
rs1571878
rs13426947
rs8026898
rs225433
rs28411352
rs60733400
rs8133843
rs10985070
rs7731626
rs12764378
rs12232497
rs2233434
rs73081554
rs706778
rs2561477
All
−0.10
−0.05
0.00
0.05
MR leave−one−out sensitivity analysis for
'RA' on 'PD'

N

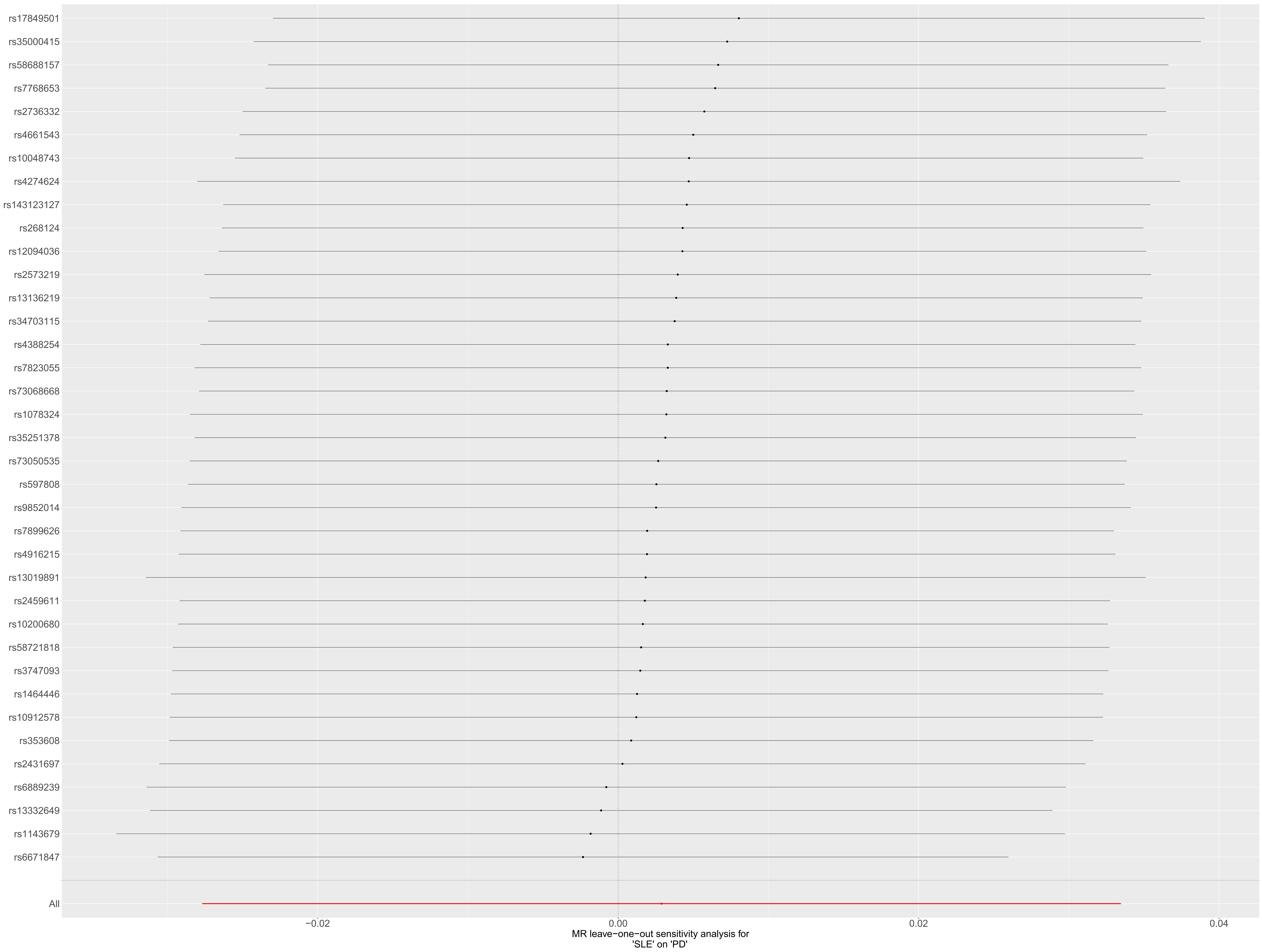
rs17849501
rs35000415
rs58688157
rs7768653
rs2736332
rs4661543
rs10048743
rs4274624
rs143123127
rs268124
rs12094036
rs2573219
rs13136219
rs34703115
rs4388254
rs7823055
rs73068668
rs1078324
rs35251378
rs73050535
rs597808
rs9852014
rs7899626
rs4916215
rs13019891
rs2459611
rs10200680
rs58721818
rs3747093
rs1464446
rs10912578
rs353608
rs2431697
rs6889239
rs13332649
rs1143679
rs6671847
All
−0.02
0.00
0.02
0.04
MR leave−one−out sensitivity analysis for
'SLE' on 'PD'

O

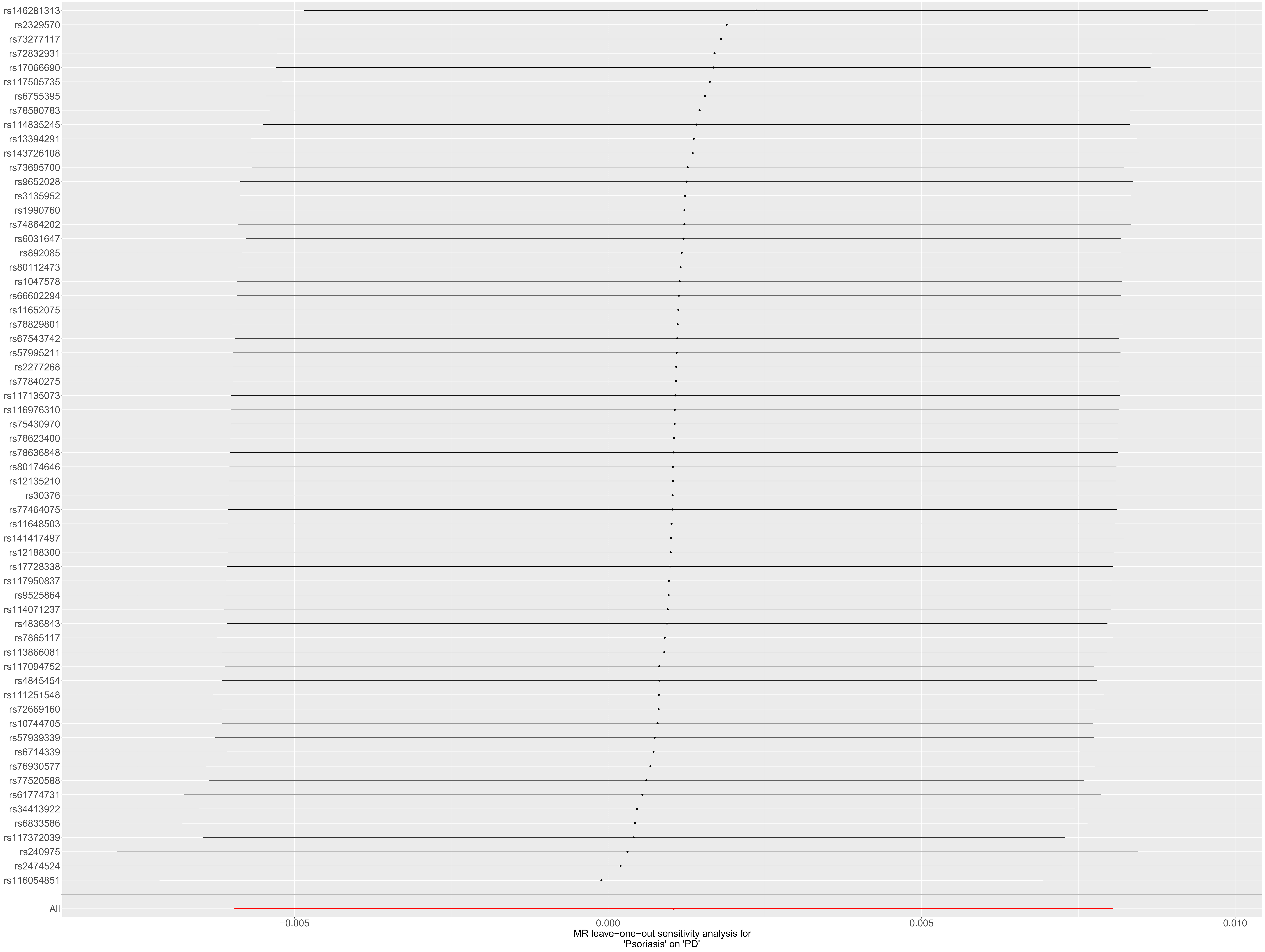

rs146281313
rs2329570
rs73277117
rs72832931
rs17066690
rs117505735
rs6755395
rs78580783
rs114835245
rs13394291
rs143726108
rs73695700
rs9652028
rs3135952
rs1990760
rs74864202
rs6031647
rs892085
rs80112473
rs1047578
rs66602294
rs11652075
rs78829801
rs67543742
rs57995211
rs2277268
rs77840275
rs117135073
rs116976310
rs75430970
rs78623400
rs78636848
rs80174646
rs12135210
rs30376
rs77464075
rs11648503
rs141417497
rs12188300
rs17728338
rs117950837
rs9525864
rs114071237
rs4836843
rs7865117
rs113866081
rs117094752
rs4845454
rs111251548
rs72669160
rs10744705
rs57939339
rs6714339
rs76930577
rs77520588
rs61774731
rs34413922
rs6833586
rs117372039
rs240975
rs2474524
rs116054851
All
−0.005
0.000
0.005
0.010
MR leave−one−out sensitivity analysis for
'Psoriasis' on 'PD'

P

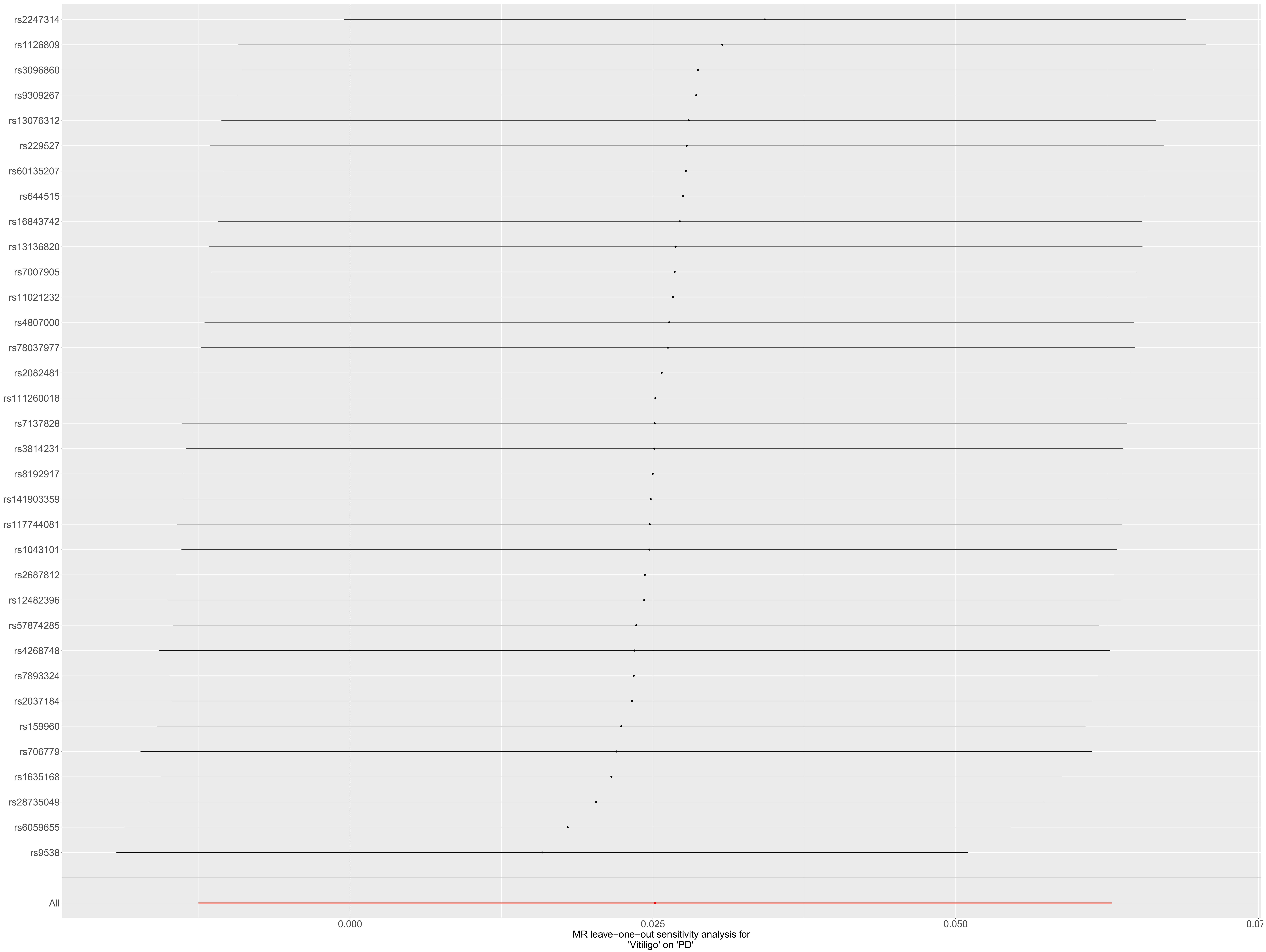
rs2247314
rs1126809
rs3096860
rs9309267
rs13076312
rs229527
rs60135207
rs644515
rs16843742
rs13136820
rs7007905
rs11021232
rs4807000
rs78037977
rs2082481
rs111260018
rs7137828
rs3814231
rs8192917
rs141903359
rs117744081
rs1043101
rs2687812
rs12482396
rs57874285
rs4268748
rs7893324
rs2037184
rs159960
rs706779
rs1635168
rs28735049
rs6059655
rs9538
All
0.000
0.025
0.050
0.07
MR leave−one−out sensitivity analysis for
'Vitiligo' on 'PD'
