# Supplementary material for: Investigation of the causal association between Parkinson’s disease and autoimmune disorders: a bidirectional Mendelian randomization study
Source: Front Immunol. 2024 May 7;15:1370831. doi: 10.3389/fimmu.2024.1370831 (PMC11106379; doi:10.3389/fimmu.2024.1370831)
Supplement: Supplementary file 5 [file Table_1.docx]

# Supplementary Table 1. STROBE-MR Checklist.

| Item | Relevant paragraph from manuscript |
| --- | --- |
| **1. Title and Abstract:** Indicate Mendelian randomization (MR) as the study’s design in the title and/or the abstract if that is a main purpose of the study. | Title and Abstract sections. |
| **Introduction** |  |
| 1. **Background:** Explain the scientific background and rationale for the reported study. What is the exposure? Is a potential causal relationship between exposure and outcome plausible? Justify why MR is a helpful method to address the study question | Described in the 1st and 2nd paragraphs of the Introduction section. |
| 1. **Objectives:** State specific objectives clearly, including prespecified causal hypotheses (if any). State that MR is a method that, under specific assumptions, intends to estimate causal effects | Described in the 3rd paragraph of the Introduction section. |
| **Methods** |  |
| 1. **Study design and data sources:** Present key elements of the study design early in the article. Consider including a table listing sources of data for all phases of the study. For each data source contributing to the analysis, describe the following:   a) Setting: Describe the setting, locations, and relevant dates, including periods of recruitment, exposure, follow-up, and data collection, when available  b) Participants: Give the eligibility criteria, and the sources and methods of selection of participants. Report the sample size, and whether any power or sample size calculations were carried out prior to the main analysis  c) Describe measurement, quality control and selection of genetic variants  d) For each exposure, outcome, and other relevant variables, describe methods of assessment and diagnostic criteria for diseases  e) Provide details of ethics committee approval and participant informed consent, if relevant | Described in the 1st-3rd and 5th paragraphs of the Methods section, Table 1 and Figure 1. |
| 1. **Assumptions:** Explicitly state the three core IV assumptions for the main analysis (relevance, independence and exclusion restriction) as well assumptions for any additional or sensitivity analysis | Described in the 1st paragraph of the Methods section. |
| 1. **Statistical methods main analysis:**   Describe statistical methods and statistics used  a) Describe how quantitative variables were handled in the analyses (i.e., scale, units, model)  b) Describe how genetic variants were handled in the analyses and, if applicable, how their weights were selected  c) Describe the MR estimator (e.g., two stage least squares, Wald ratio) and related statistics. Detail the included covariates and, in case of two sample MR, whether the same covariate set was used for adjustment in the two samples  d) Explain how missing data were addressed.  e) If applicable, indicate how multiple testing was addressed | Described in the 4th-7th paragraphs of the Methods section. |
| 1. **Assessment of assumptions:** Describe any methods or prior knowledge used to assess the assumptions or justify their validity | Described in the 4th and 5th paragraphs of the Methods section. |
| 1. **Sensitivity analyses:** Describe any sensitivity analyses or additional analyses performed (e.g., comparison of effect estimates from different approaches, independent replication, bias analytic techniques, validation of instruments, simulations) | Described in the 4th and 5th paragraphs of the Methods section. |
| 1. **Software and pre-registration**   a) Name statistical software and package(s), including version and settings used  b) State whether the study protocol and details were pre-registered (as well as when and where) | Described in the 7th paragraph of the Methods section. |
| **Results** |  |
| 1. **Descriptive data**   a) Report the numbers of individuals at each stage of included studies and reasons for exclusion. Consider use of a flow-diagram  b) Report summary statistics for phenotypic exposure(s), outcome(s) and other relevant variables (e.g. means, standard deviations, proportions)  c) If the data sources include meta-analyses of previous studies, provide the assessments of heterogeneity across these studies  d) For two-sample MR:  i. Provide justification of the similarity of the genetic variant-exposure associations between the exposure and outcome samples  ii. Provide information on the number of individuals who overlap between the exposure and outcome studies | Described in the 1st and 5th paragraph of the Results section, Table 1, Table 2, Table 3, and Figure 1. Additionally, there is no significant overlap between the exposure and outcome samples. |
| 1. **Main results**   a) Report the associations between genetic variant and exposure, and between genetic variant and outcome, preferably on an interpretable scale  b) Report MR estimates of the relationship between exposure and outcome, and the measures of uncertainty from the MR analysis, on an interpretable scale, such as odds ratio or relative risk per SD difference  c) If relevant, consider translating estimates of relative risk into absolute risk for a meaningful time-period  d) Consider any plots to visualize results (e.g. forest plot, scatterplot of associations between genetic variants and outcome versus between genetic variants and exposure) | Reported in the 2nd, 3rd, 4th, and 6th paragraphs of the Results section, Table 2, Table 3, Figure 2, Figure 3, Figure 4, Figure 5, Supplementary Figure 1 and Supplementary Figure 3. |
| 1. **Assessment of assumptions**   a) Report the assessment of the validity of the assumptions  b) Report any additional statistics (e.g., assessments of heterogeneity across genetic variants, such as I2, Q statistic or E-value) | Reported in the 2nd, 3rd, 4th, and 6th paragraphs of the Results section, Table 2, Table 3, Figure 2, Figure 3, Figure 4, Figure 5, Supplementary Table 1, Supplementary Table 2, Supplementary Figure 1 and Supplementary Figure 3. |
| 1. **Sensitivity and additional analyses**   a) Report any sensitivity analyses to assess the robustness of the main results to violations of the assumptions  b) Report results from other sensitivity analyses or additional analyses  c) Report any assessment of direction of causal relationship (e.g., bidirectional MR)  d) When relevant, report and compare with estimates from non-MR analyses  e) Consider additional plots to visualize results (e.g., leave-one-out analyses) | Reported in the 2nd, 3rd, 4th, and 6th paragraphs of the Results section, Table 2, Table 3, Supplementary Table 1 and Supplementary Table 2, Supplementary Figure 2 and Supplementary Figure 4. |
| **Discussion** |  |
| 1. **Key results**   Summarize key results with reference to study objectives | Described in the 1st paragraph of the Discussion section. |
| 1. **Limitations**   Discuss limitations of the study, taking into account the validity of the MR assumptions, other sources of potential bias, and imprecision. Discuss both direction and magnitude of any potential bias, and any efforts to address them | Described in the 5th paragraph of the Discussion section. |
| 1. **Interpretations**   a) Meaning: Give a cautious overall interpretation of results in the context of their limitations and in comparison with other studies  b) Mechanism: Discuss underlying biological mechanisms that could drive a potential causal relationship between the investigated exposure and the outcome, and whether the gene-environment equivalence assumption is reasonable. Use causal language carefully, clarifying that IV estimates may provide causal effects only under certain assumptions  c) Clinical relevance: Discuss whether the results have clinical or public policy relevance, and to what extent they inform effect sizes of possible interventions | Described in the 1st, 2nd, 3rd, and 4th paragraph of the Discussion and Conclusion section. |
| 1. **Generalizability:**   Discuss the generalizability of the study results (a) to other populations, (b) across other exposure periods/timings, and (c) across other levels of exposure | Described in the 5th paragraph of the Discussion section. |
| 1. **Funding:**   Describe sources of funding and the role of funders in the present study and, if applicable, sources of funding for the databases and original study or studies on which the present study is based | Described in the Funding section. |
| 1. **Data and data sharing:**   Provide the data used to perform all analyses or report where and how the data can be accessed, and reference these sources in the article. Provide the statistical code needed to reproduce the results in the article, or report whether the code is publicly accessible and if so, where | Described in the Data availability statement section. |
| 1. **Conflict of Interest:** All authors should declare all potential conflicts of interest | Described in the Conflict of interest section. |

This checklist is copyrighted by the Equator Network under the Creative Commons Attribution 3.0 Unported (CC BY 3.0) license.

1. Skrivankova VW, Richmond RC, Woolf BAR, Yarmolinsky J, Davies NM, Swanson SA, et al. Strengthening the Reporting of Observational Studies in Epidemiology using Mendelian Randomization (STROBE-MR) Statement. JAMA. 2021;under review.

2. Skrivankova VW, Richmond RC, Woolf BAR, Davies NM, Swanson SA, VanderWeele TJ, et al. Strengthening the Reporting of Observational Studies in Epidemiology using Mendelian Randomisation (STROBE-MR): Explanation and Elaboration. BMJ. 2021;375:n2233.
