# Supplementary material for: Investigation of the causal association between Parkinson’s disease and autoimmune disorders: a bidirectional Mendelian randomization study
Source: Front Immunol. 2024 May 7;15:1370831. doi: 10.3389/fimmu.2024.1370831 (PMC11106379; doi:10.3389/fimmu.2024.1370831)
Supplement: Supplementary file 6 [file Table_2.docx]

| Outcome | Heterogeneity test | | Tests for directional horizontal pleiotropy | | | | | | | | Test for causal direction |
| --- | --- | --- | --- | --- | --- | --- | --- | --- | --- | --- | --- |
|  | Inverse variance weighted | | MR Egger intercept | | MR-PRESSO global test | MR-PRESSO distortion test | MR-PRESSO  Outlier test | | Outlier-corrected | | Steiger test |
|  | Q | Q_pval | Intercept | *P*-value | *P*-value | *P*-value | *P*-value | Outlier SNPs | OR (95%CI) | *P*-value | Correct causal direction |
| MS | 129.34 | 9e-04 | 0.009 | 0.112 | < 0.001 | 0.704 | < 0.084 | rs7134559 | 1.01  (0.94-1.04) | 0.576 | TRUE |
| NMOSD | 87.49 | 0.376 | -0.023 | 0.442 | 0.359 | NA | NA | NA | NA | NA | FALSE |
| MG | 107.75 | 0.049 | 0.014 | 0.186 | 0.039 | NA | NA | NA | NA | NA | TRUE |
| Asthma | 121.97 | 0.007 | -0.001 | 0.515 | 0.008 | 0.531 | < 0.087 | rs11150601 | 0.99  (0.97-1.00) | 0.183 | TRUE |
| IBD | 153.71 | 0.000 | -0.002 | 0.764 | < 0.001 | 0.974 | < 0.082 | rs2904880  rs12951632 | 1.04  (0.99-1.10) | 0.133 | TRUE |
| CD | 155.21 | 0.000 | 0.003 | 0.777 | < 0.001 | 0.871 | < 0.083 | rs2904880 | 1.07  (0.99-1.16) | 0.099 | TRUE |
| UC | 123.66 | 0.003 | -0.004 | 0.566 | 0.006 | 0.541 | < 0.084 | rs76904798 | 1.03  (0.96-1.10) | 0.358 | TRUE |
| IBS | 123.66 | 0.003 | 0.001 | 0.690 | 0.006 | NA | NA | NA | NA | NA | TRUE |
| T1D | 129.84 | 0.001 | -0.005 | 0.549 | < 0.001 | 0.805 | < 0.086 | rs2904880 | 1.09  (1.02-1.17) | 0.015 | TRUE |
| RA | 112.11 | 0.004 | -0.008 | 0.202 | 0.006 | 0.991 | < 0.076 | rs26431  rs2904880 | 0.98  (0.94-1.03) | 0.430 | TRUE |
| SLE | 141.41 | 0.000 | -0.010 | 0.364 | < 0.001 | 0.206 | < 0.075 | rs62053943 | 0.98  (0.89-1.09) | 0.712 | TRUE |
| Vitiligo | 147.35 | 0.000 | 0.015 | 0.151 | < 0.001 | 0.866 | < 0.084 | rs6497339 | 0.93  (0.85-1.02) | 0.116 | TRUE |

**Supplementary Table 2. Test results of heterogeneity, directional horizontal pleiotropy and causal direction in forward MR analyses.**

MR, Mendelian Randomization; SNP, single-nucleotide polymorphism; MR-PRESSO, Mendelian randomization pleiotropy residual sum and outlier; Q, Cochran’s Q statistic; OR, odds ratio; CI, confidence interval; MS, multiple sclerosis; NMOSD, neuromyelitis optica spectrum disorder; MG, myasthenia gravis; IBD, inflammatory bowel disease; CD, Crohn’s disease; UC, ulcerative colitis; IBS, irritable bowel syndrome; T1D, type 1 diabetes; RA, rheumatoid arthritis; SLE, systemic lupus erythematosus; NA, not available.
