# Supplementary material for: Investigation of the causal association between Parkinson’s disease and autoimmune disorders: a bidirectional Mendelian randomization study
Source: Front Immunol. 2024 May 7;15:1370831. doi: 10.3389/fimmu.2024.1370831 (PMC11106379; doi:10.3389/fimmu.2024.1370831)
Supplement: Supplementary file 7 [file Table_3.docx]

**Supplementary Table 3. Test results of heterogeneity, directional horizontal pleiotropy and causal direction in reverse MR analyses.**

| Exposure | Heterogeneity test | | Tests for directional horizontal pleiotropy | | | | | | | | Test for causal direction |
| --- | --- | --- | --- | --- | --- | --- | --- | --- | --- | --- | --- |
|  | Inverse variance weighted | | MR Egger intercept | | MR-PRESSO global test | MR-PRESSO distortion test | MR-PRESSO  Outlier test | | Outlier-corrected | | Steiger test |
|  | Q | Q_*p*val | Intercept | *P*-value | *P*-value | *P*-value | *P*-value | Outlier SNPs | OR (95%CI) | *P*-value | Correct causal direction |
| MS | 72.88 | 0.106 | -0.005 | 0.594 | 0.107 | NA | NA | NA | NA | NA | TRUE |
| MG | 1.65 | 0.648 | 0.054 | 0.512 | 0.676 | NA | NA | NA | NA | NA | TRUE |
| Asthma | 83.63 | 0.111 | -0.002 | 0.813 | 0.122 | NA | NA | NA | NA | NA | TRUE |
| IBD | 107.91 | 1E-04 | -0.034 | 0.025 | < 0.001 | 0.732 | < 0.058 | rs140892874  rs9370774  rs4712528 | 0.97  (0.93-1.02) | 0.242 | TRUE |
| CD | 82.69 | 0.001 | -0.023 | 0.057 | 0.003 | 0.699 | 0.048 | rs11564236  rs1056441 | 0.98  (0.95-1.01) | 0.287 | TRUE |
| UC | 50.23 | 0.016 | -0.011 | 0.599 | 0.021 | NA | NA | NA | NA | NA | TRUE |
| IBS | 6.22 | 0.286 | 0.131 | 0.317 | 0.334 | NA | NA | NA | NA | NA | TRUE |
| CeD | 33.01 | 0.277 | -0.007 | 0.340 | 0.264 | NA | NA | NA | NA | NA | TRUE |
| PBC | 54.83 | 0.030 | 0.003 | 0.787 | 0.033 | 0.989 | 0.038 | rs3784099  rs859767 | 0.98  (0.95-1.01) | 0.217 | TRUE |
| PSC | 7.51 | 0.756 | 0.013 | 0.646 | 0.738 | NA | NA | NA | NA | NA | TRUE |
| T1D | 35.34 | 0.270 | 0.017 | 0.101 | 0.219 | NA | NA | NA | NA | NA | TRUE |
| AS | 14.91 | 0.728 | -0.011 | 0.427 | 0.759 | NA | NA | NA | NA | NA | TRUE |
| RA | 55.14 | 0.007 | -0.016 | 0.428 | 0.009 | 0.707 | < 0.033 | rs2561477 | 0.96  (0.90-1.03) | 0.232 | TRUE |
| SLE | 64.40 | 0.003 | 0.011 | 0.360 | 0.001 | NA | NA | NA | NA | NA | TRUE |
| Psoriasis | 79.75 | 0.199 | 0.000 | 0.950 | 0.199 | NA | NA | NA | NA | NA | TRUE |
| Vitiligo | 56.52 | 0.007 | -0.008 | 0.622 | 0.005 | 0.989 | 0.034 | rs9538  rs2247314 | 1.03  (0.99-1.06) | 0.134 | TRUE |

MR, Mendelian Randomization; SNP, single-nucleotide polymorphism; MR-PRESSO, Mendelian randomization pleiotropy residual sum and outlier; Q, Cochran’s Q statistic; OR, odds ratio; CI, confidence interval; MS, multiple sclerosis; MG, myasthenia gravis; IBD, inflammatory bowel disease; CD, Crohn’s disease; UC, ulcerative colitis; IBS, irritable bowel syndrome; CeD, celiac disease; PBC, primary biliary cirrhosis; PSC, primary sclerosing cholangitis; T1D, type 1 diabetes; AS, ankylosing spondylitis; RA, rheumatoid arthritis; SLE, systemic lupus erythematosus; NA, not available.
